# Supplementary material for: A novel immunogenic cell death signature for the prediction of prognosis and therapies in glioma
Source: PeerJ. 2023 Jul 11;11:e15615. doi: 10.7717/peerj.15615 (PMC10348309; doi:10.7717/peerj.15615)
Supplement: Supplemental Information 1 [file peerj-11-15615-s001.zip › rawa data/invasion.docx]

LN-229 Number of invaded cells (% of count)

| sample | 1 | 2 | 3 | mean±s.d. | t-test |
| --- | --- | --- | --- | --- | --- |
| scr | 110 | 96 | 98 | 101.33±7.57 |  |
| siMYD88 | 34 | 25 | 39 | 32.66±7.09 | 0.001 |

U-89 Number of invaded cells (% of count)

| sample | 1 | 2 | 3 | mean±s.d. | t-test |
| --- | --- | --- | --- | --- | --- |
| scr | 93 | 94 | 116 | 101±13 |  |
| siMYD88 | 20 | 24 | 32 | 25.33±6.11 | 0.001 |
