# Supplementary figures and images for: A novel immunogenic cell death signature for the prediction of prognosis and therapies in glioma
Source: PeerJ. 2023 Jul 11;11:e15615. doi: 10.7717/peerj.15615 (PMC10348309; doi:10.7717/peerj.15615)

A

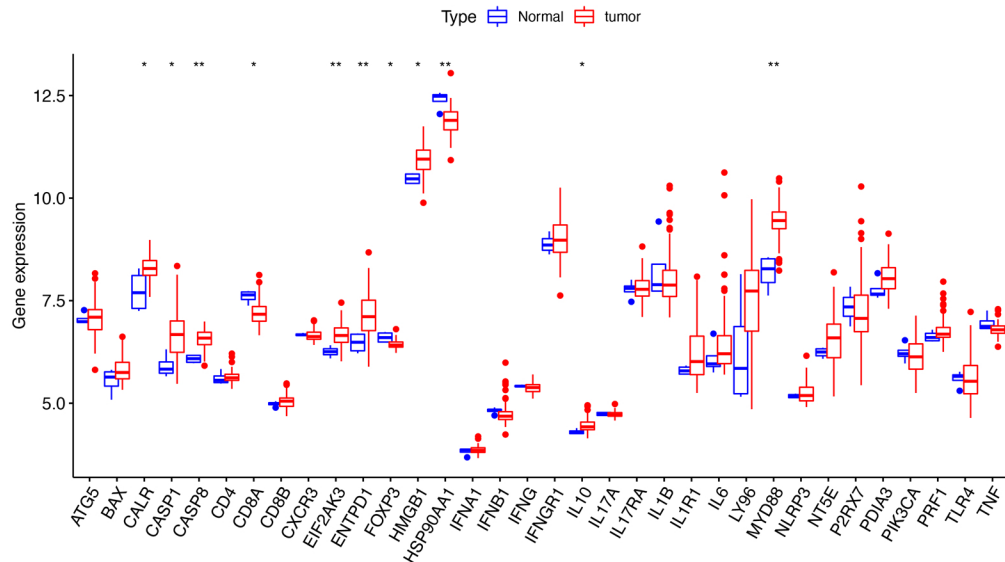

B

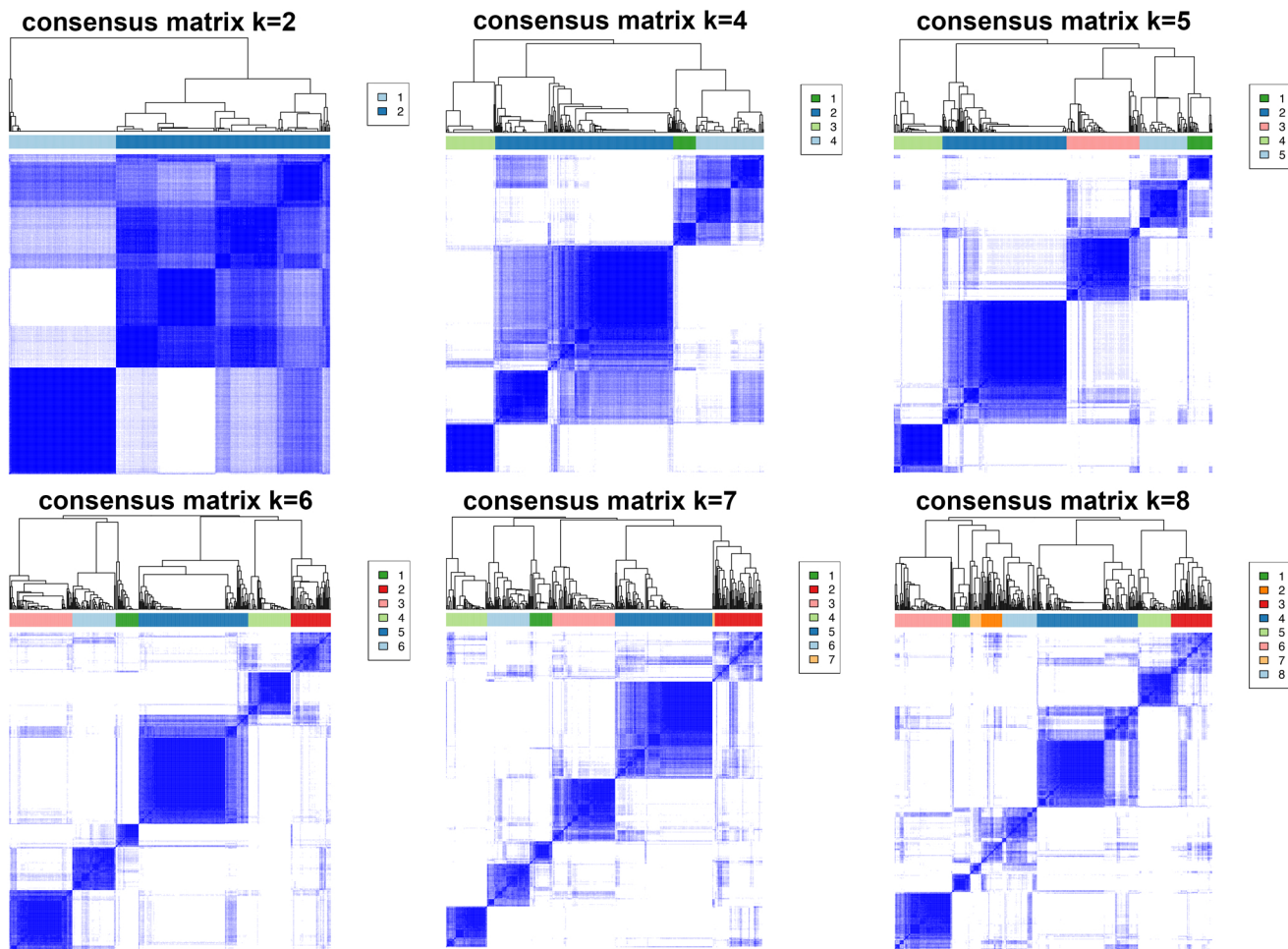

Supplement: Supplemental Information 2 — (A) Levels of ICD genes from GSE7696. (B) Consensus matrices of TCGA cohort for k = 2 to 8. [file peerj-11-15615-s002.pdf]

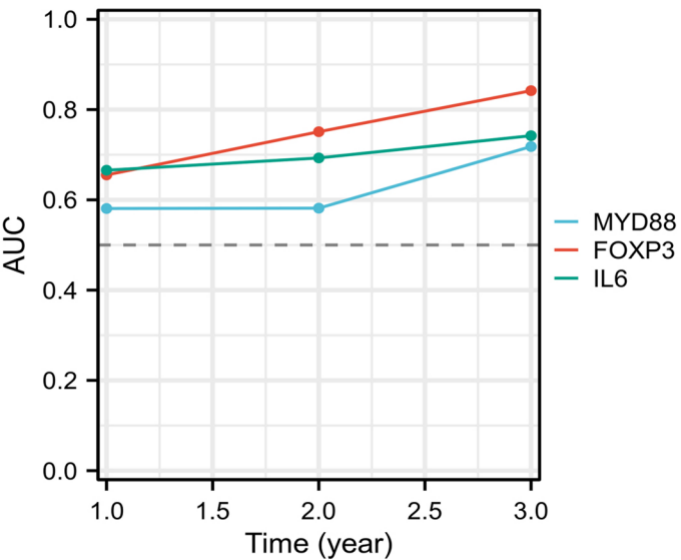

Supplement: Supplemental Information 3 [file peerj-11-15615-s003.pdf]

Type Normal tumor

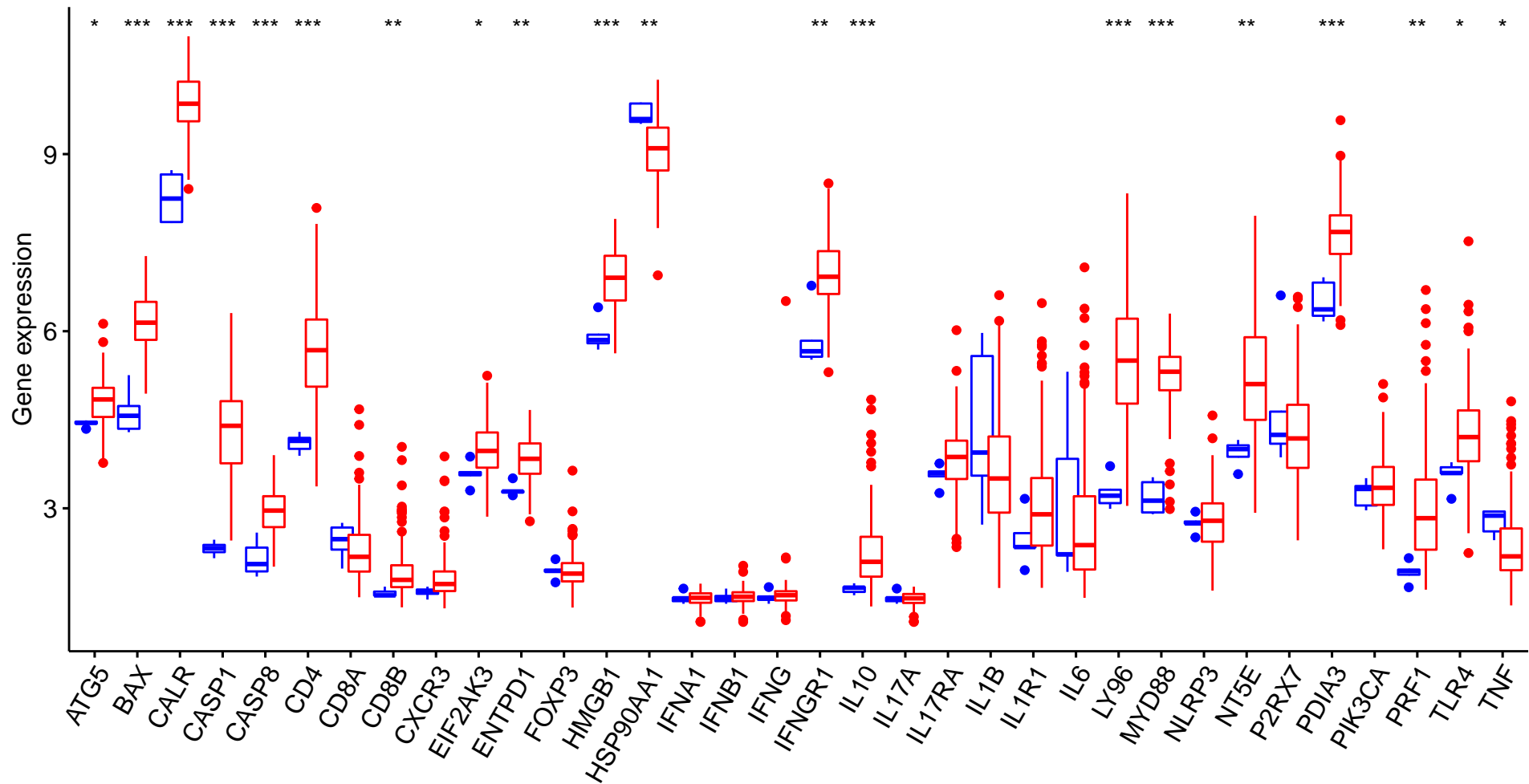

Supplement: Supplemental Information 4 [file peerj-11-15615-s004.zip › Figure 1/Figure 1E.pdf]

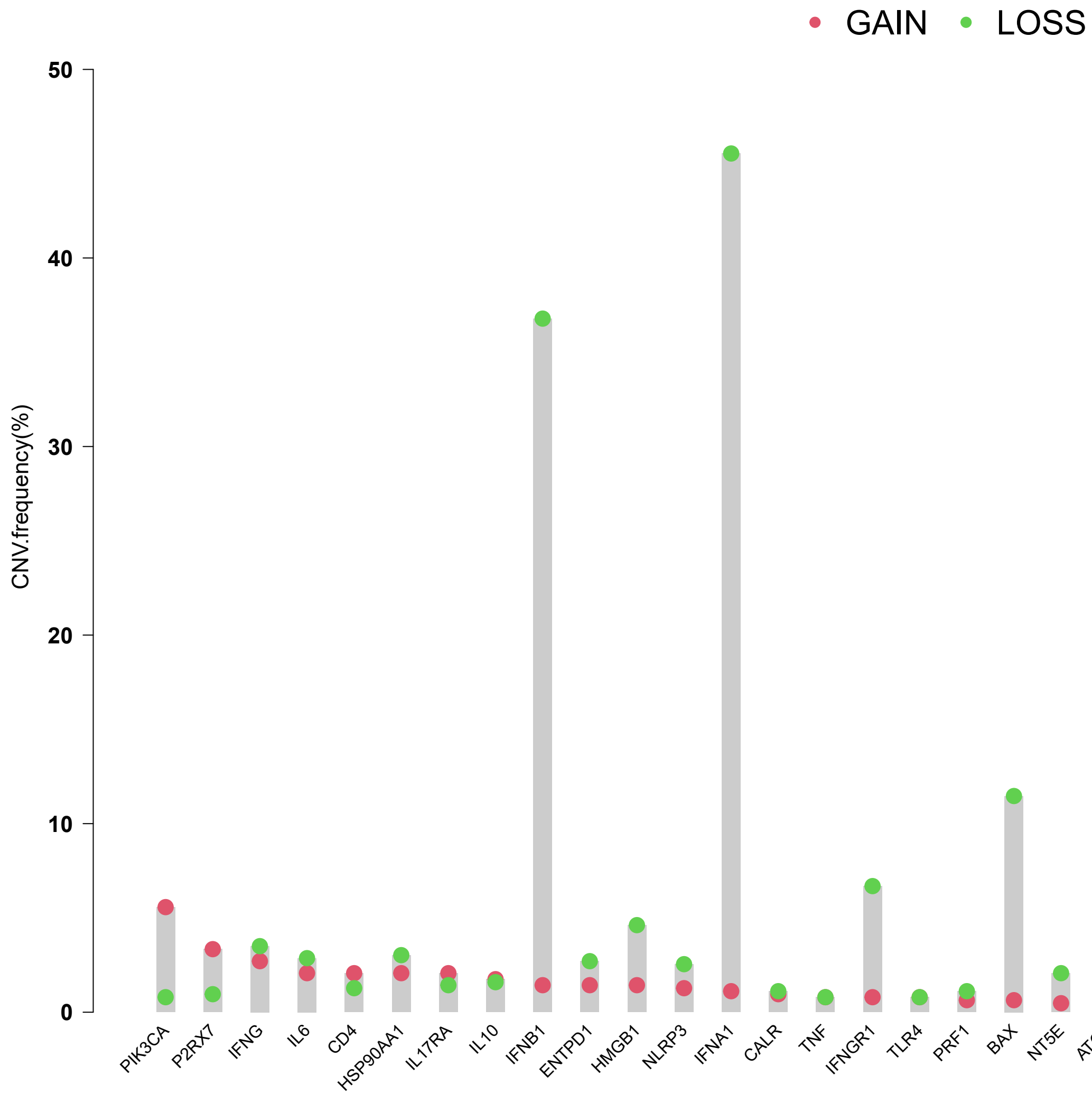

Supplement: Supplemental Information 4 [file peerj-11-15615-s004.zip › Figure 1/Figure 1D.pdf]

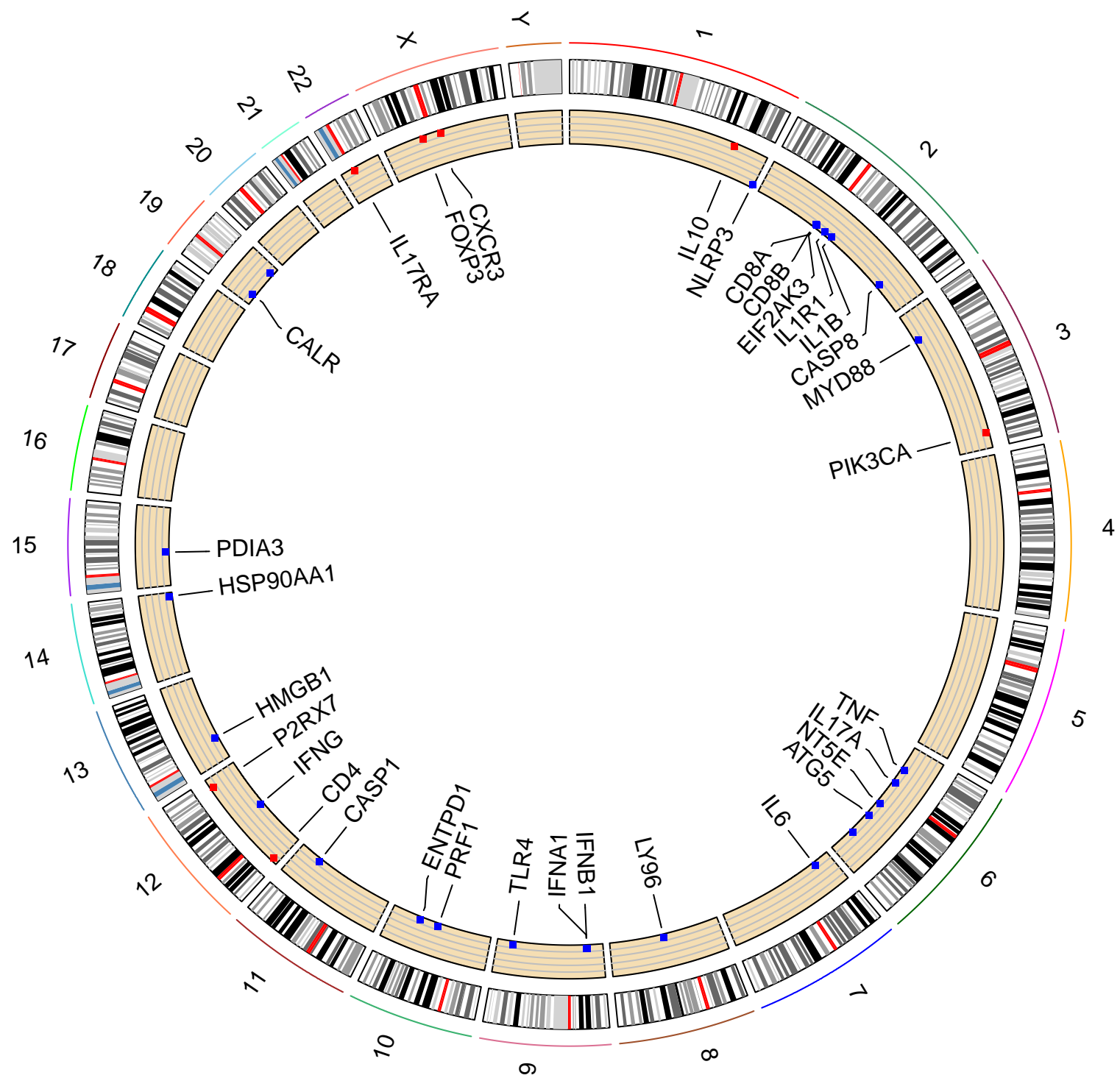

Supplement: Supplemental Information 4 [file peerj-11-15615-s004.zip › Figure 1/Figure 1C.pdf]

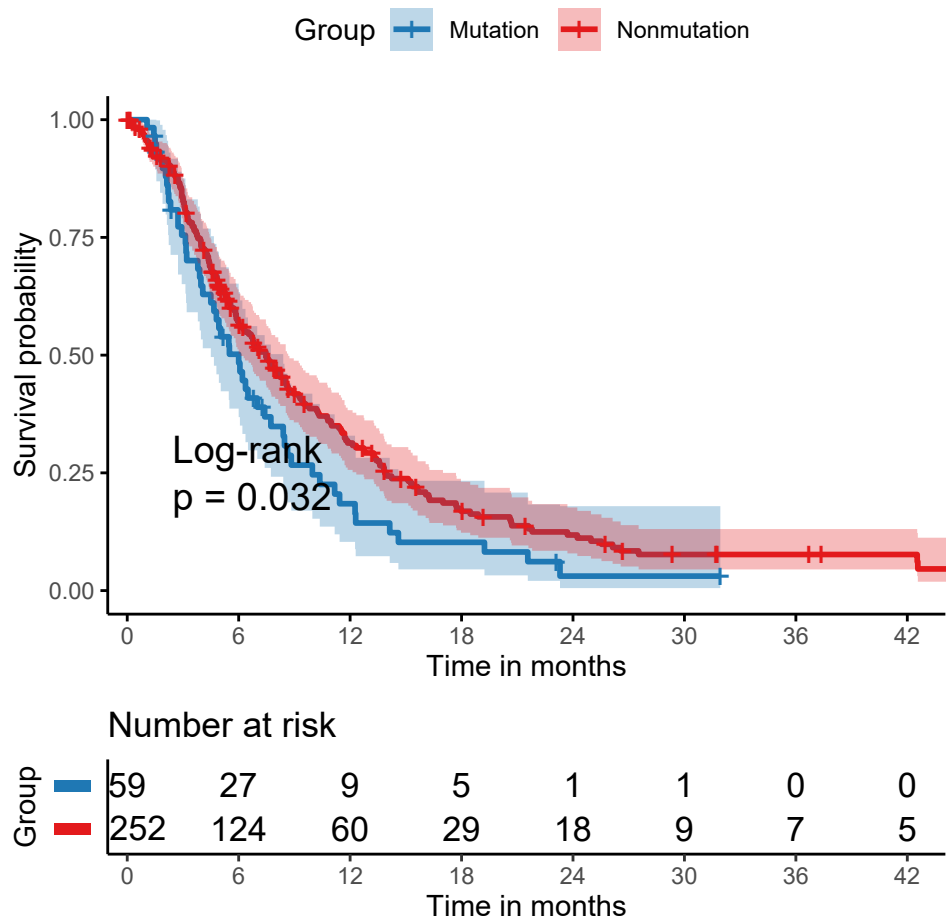

Supplement: Supplemental Information 4 [file peerj-11-15615-s004.zip › Figure 1/Figure 1B.pdf]

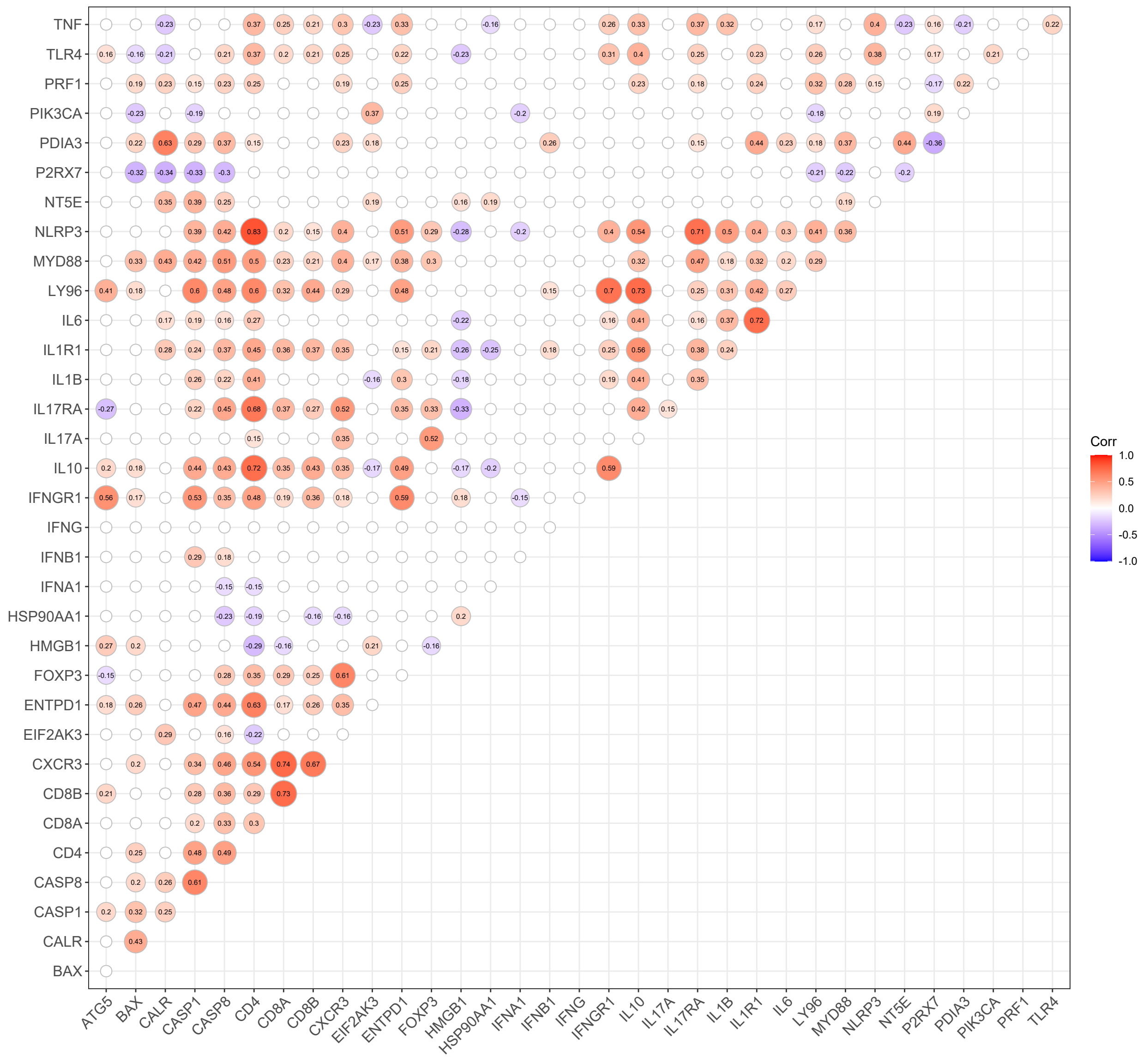

Supplement: Supplemental Information 5 [file peerj-11-15615-s005.zip › Figure 2/Figure 2A.pdf]

consensus matrix k=3

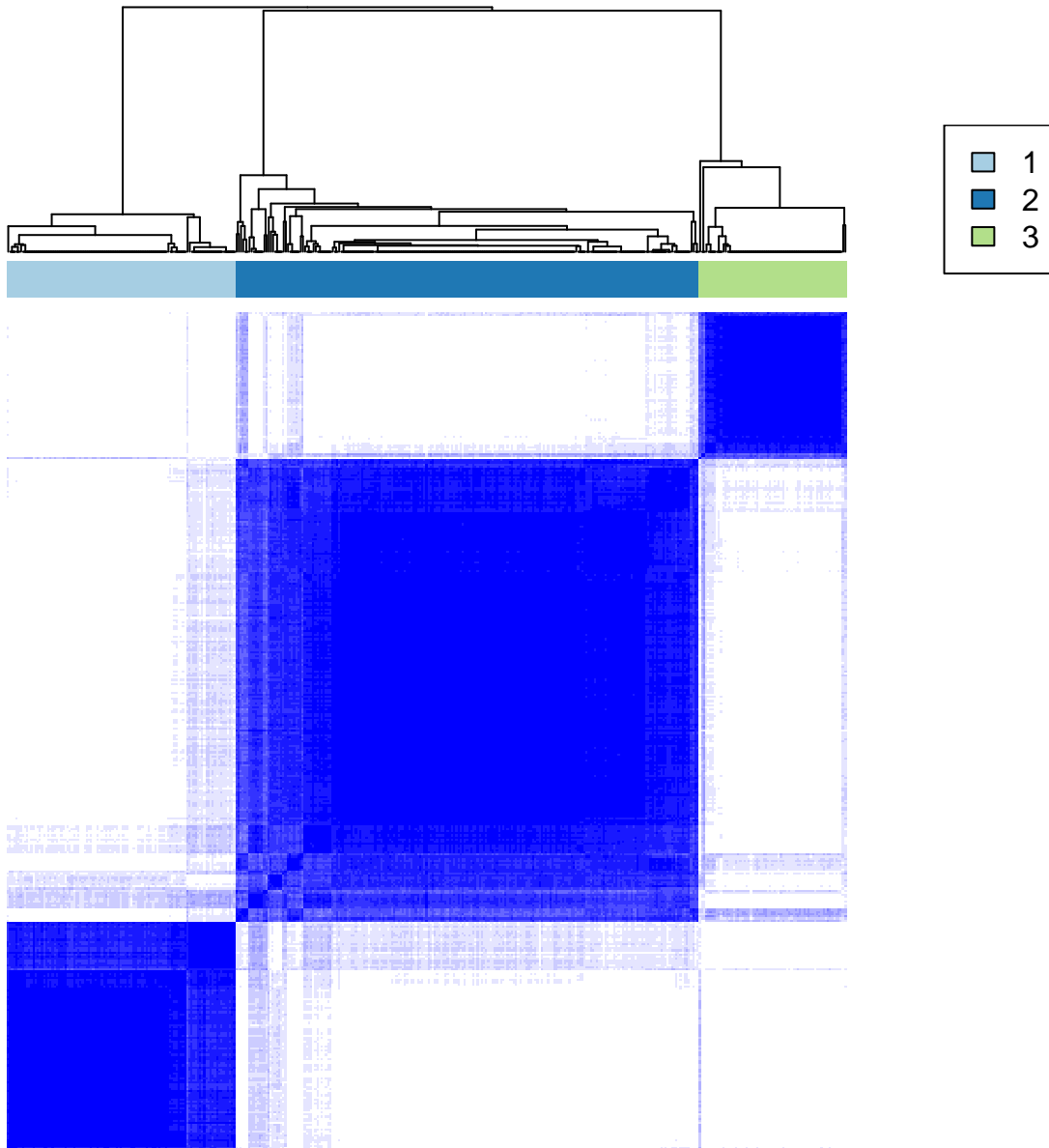

Supplement: Supplemental Information 5 [file peerj-11-15615-s005.zip › Figure 2/Figure 2C.pdf]

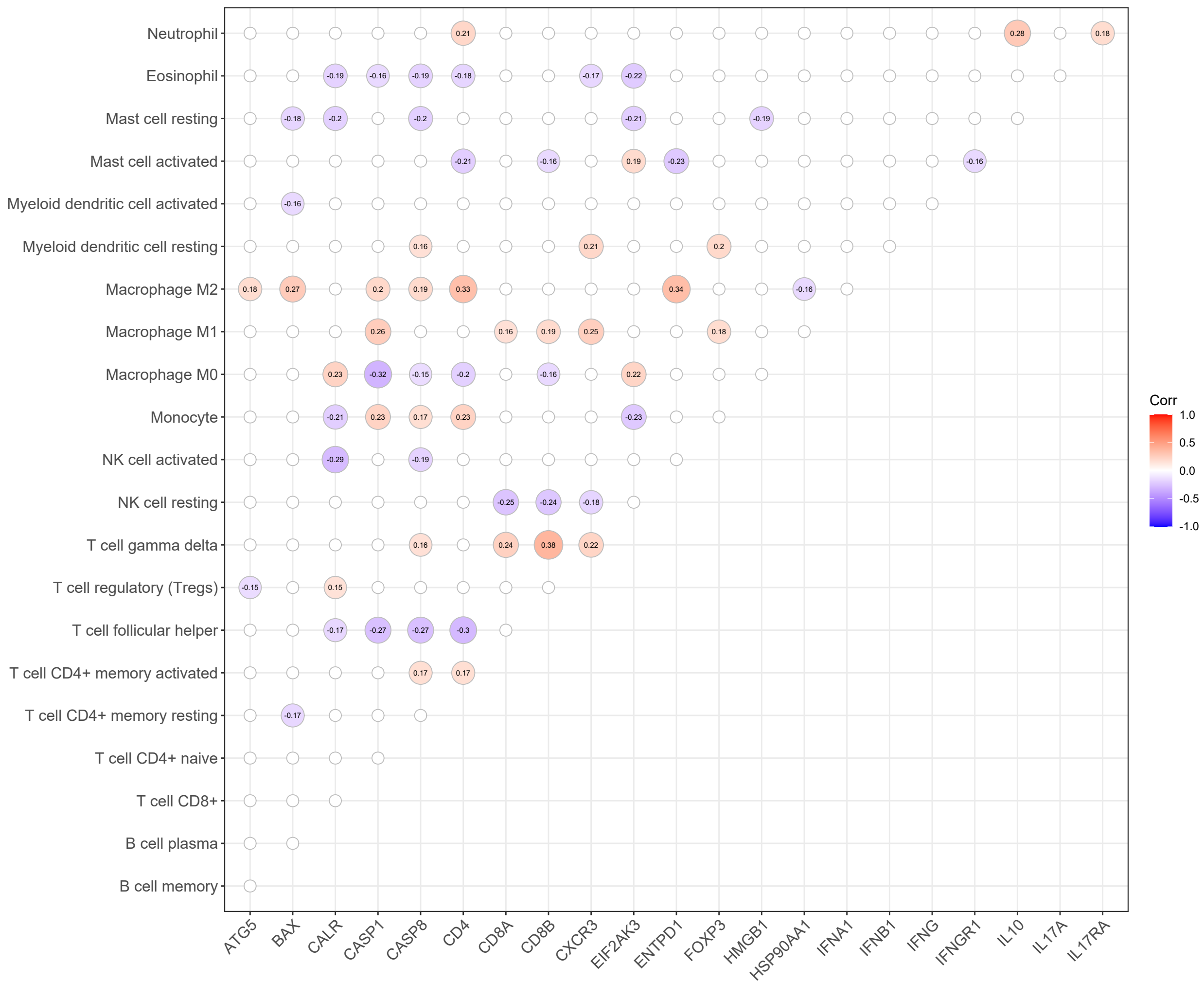

Supplement: Supplemental Information 5 [file peerj-11-15615-s005.zip › Figure 2/Figure 2B.pdf]

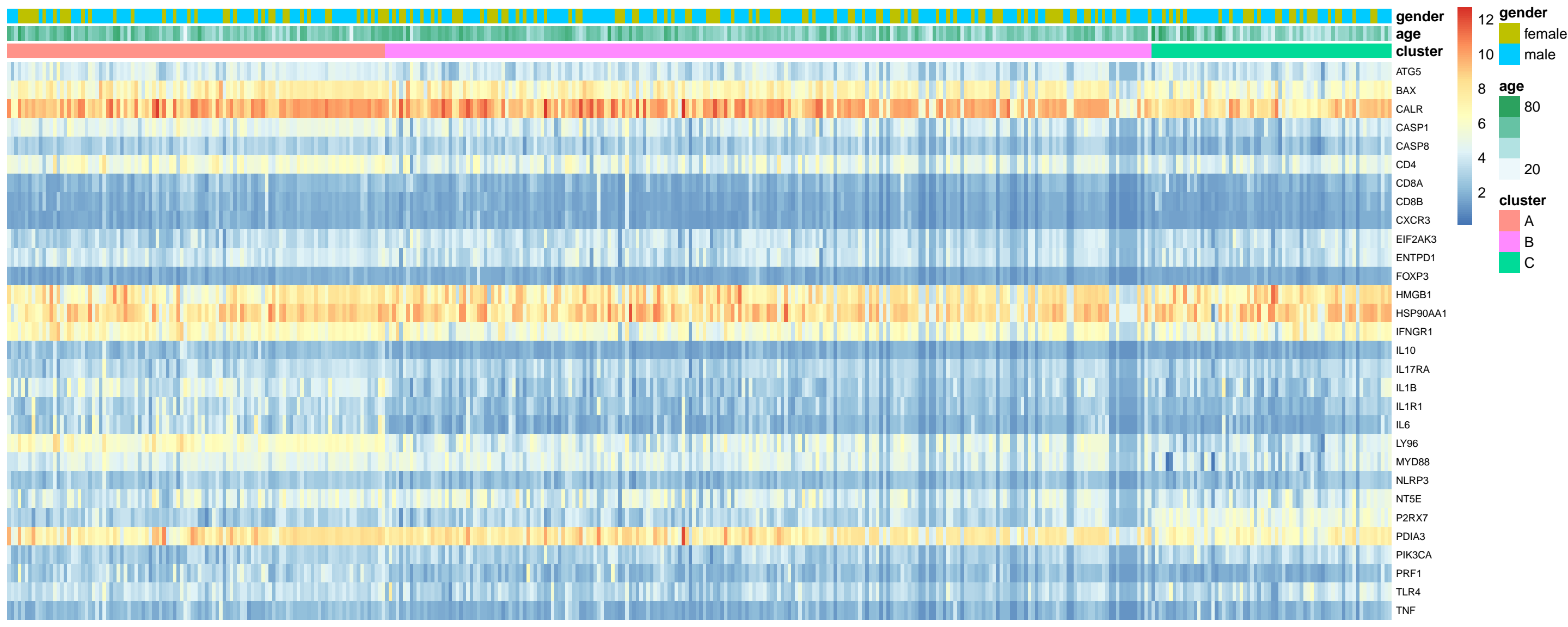

Supplement: Supplemental Information 5 [file peerj-11-15615-s005.zip › Figure 2/Figure 2F.pdf]

## Delta area

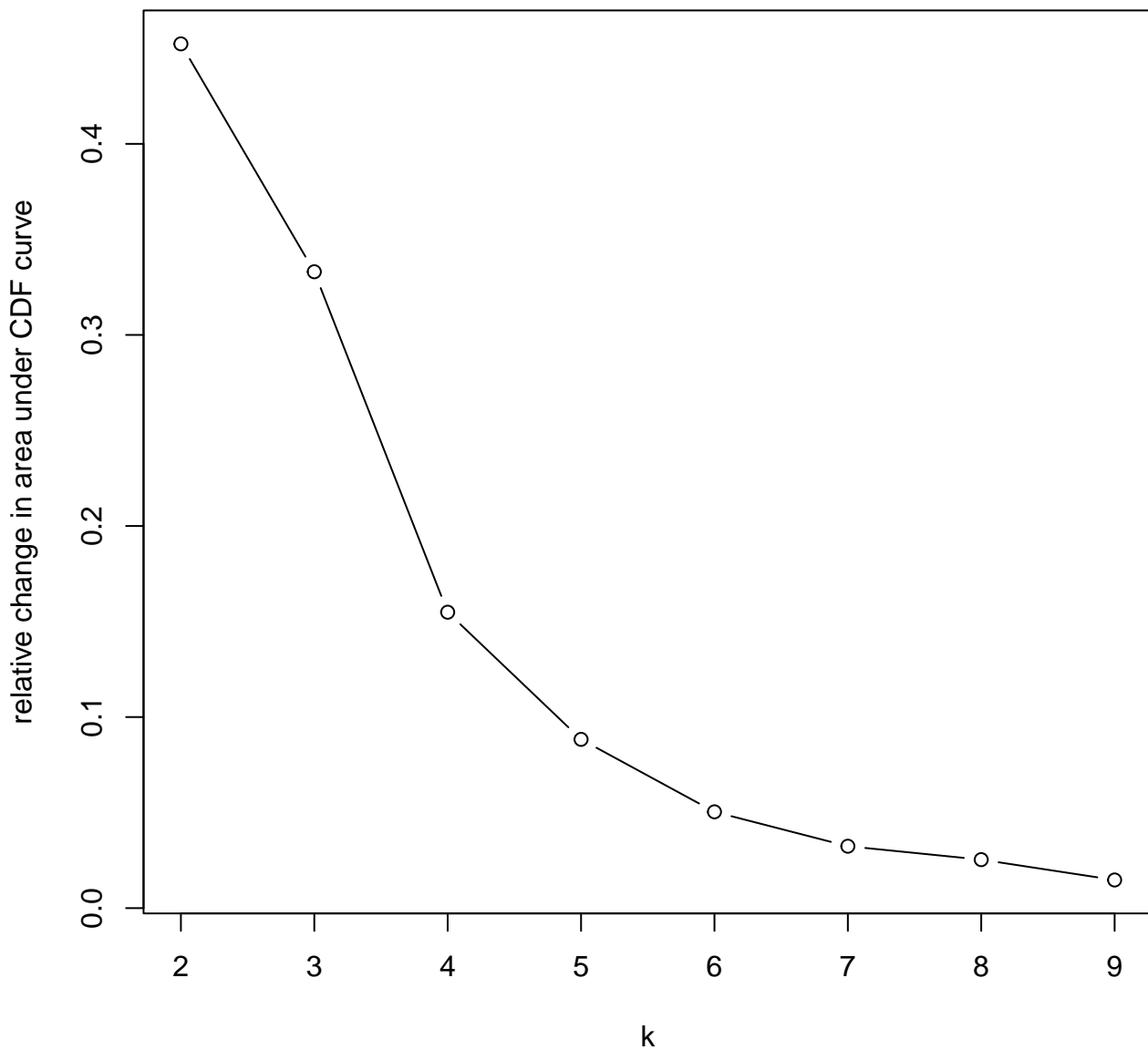

Supplement: Supplemental Information 5 [file peerj-11-15615-s005.zip › Figure 2/Figure 2E.pdf]

### consensus CDF

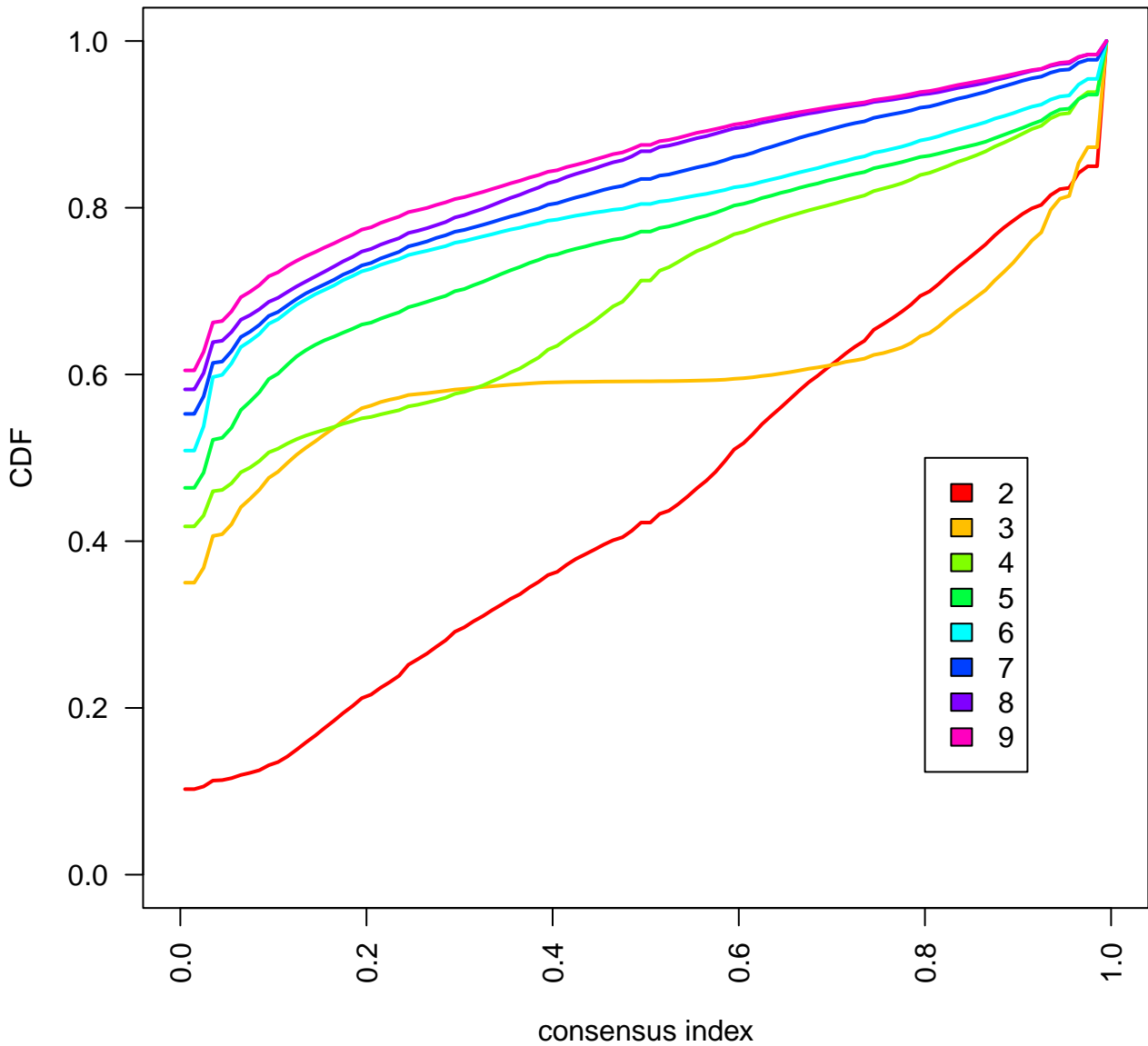

Supplement: Supplemental Information 5 [file peerj-11-15615-s005.zip › Figure 2/Figure 2D.pdf]

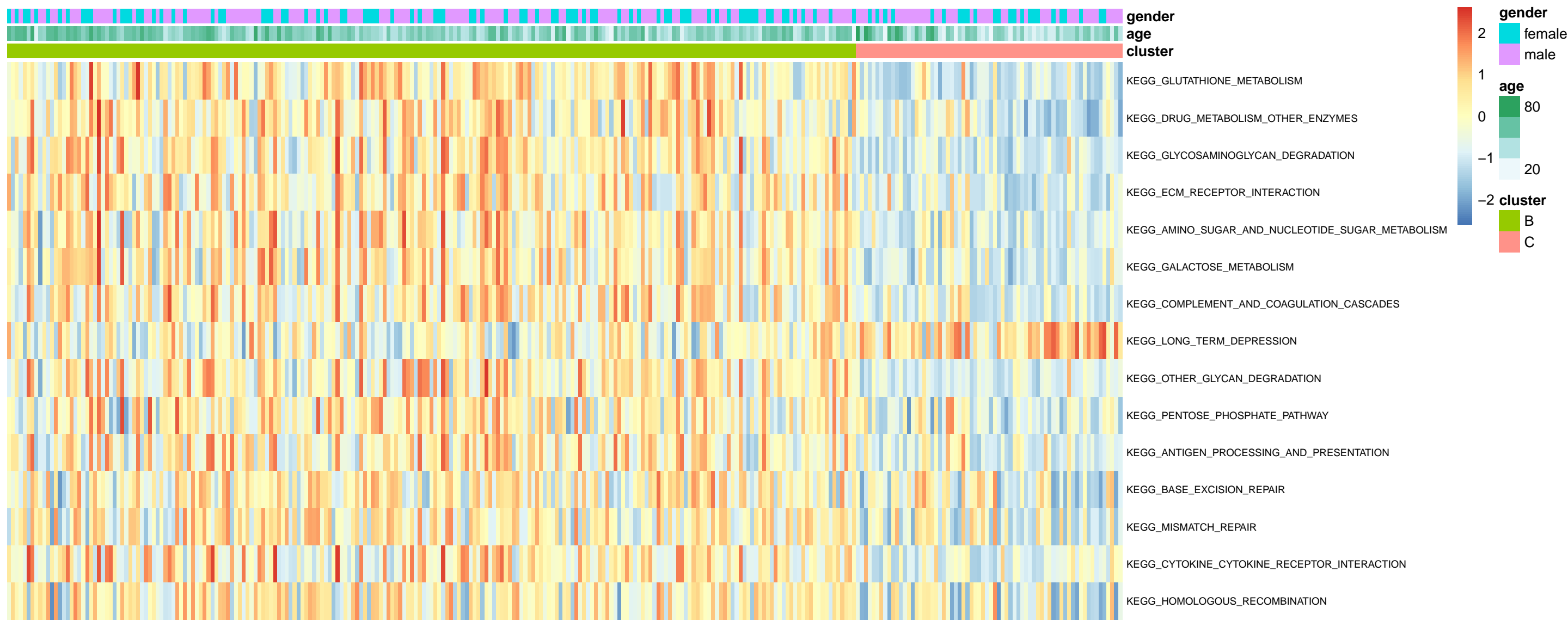

Supplement: Supplemental Information 6 [file peerj-11-15615-s006.zip › Figure 3/Figure 3D.pdf]

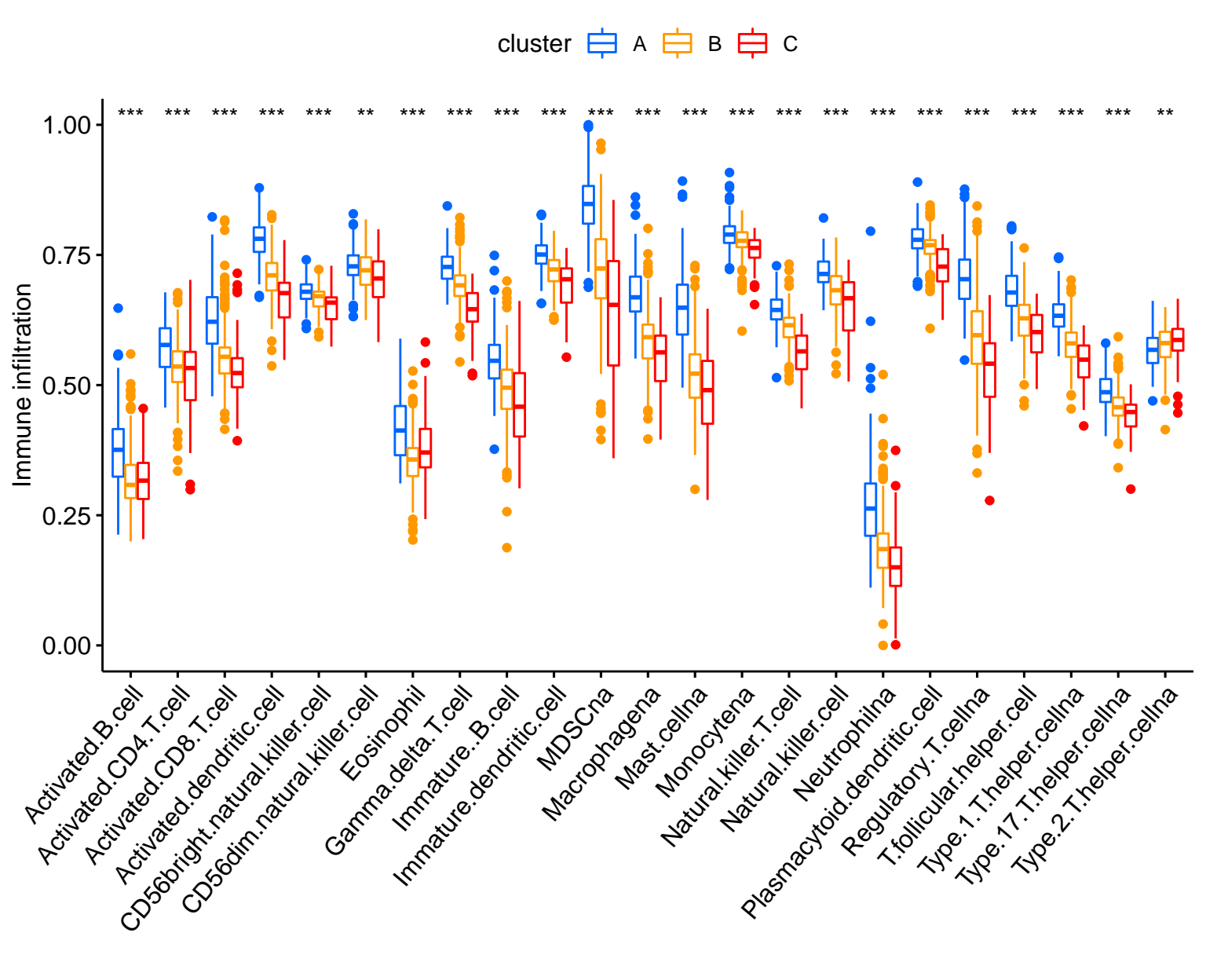

Supplement: Supplemental Information 6 [file peerj-11-15615-s006.zip › Figure 3/Figure 3E.pdf]

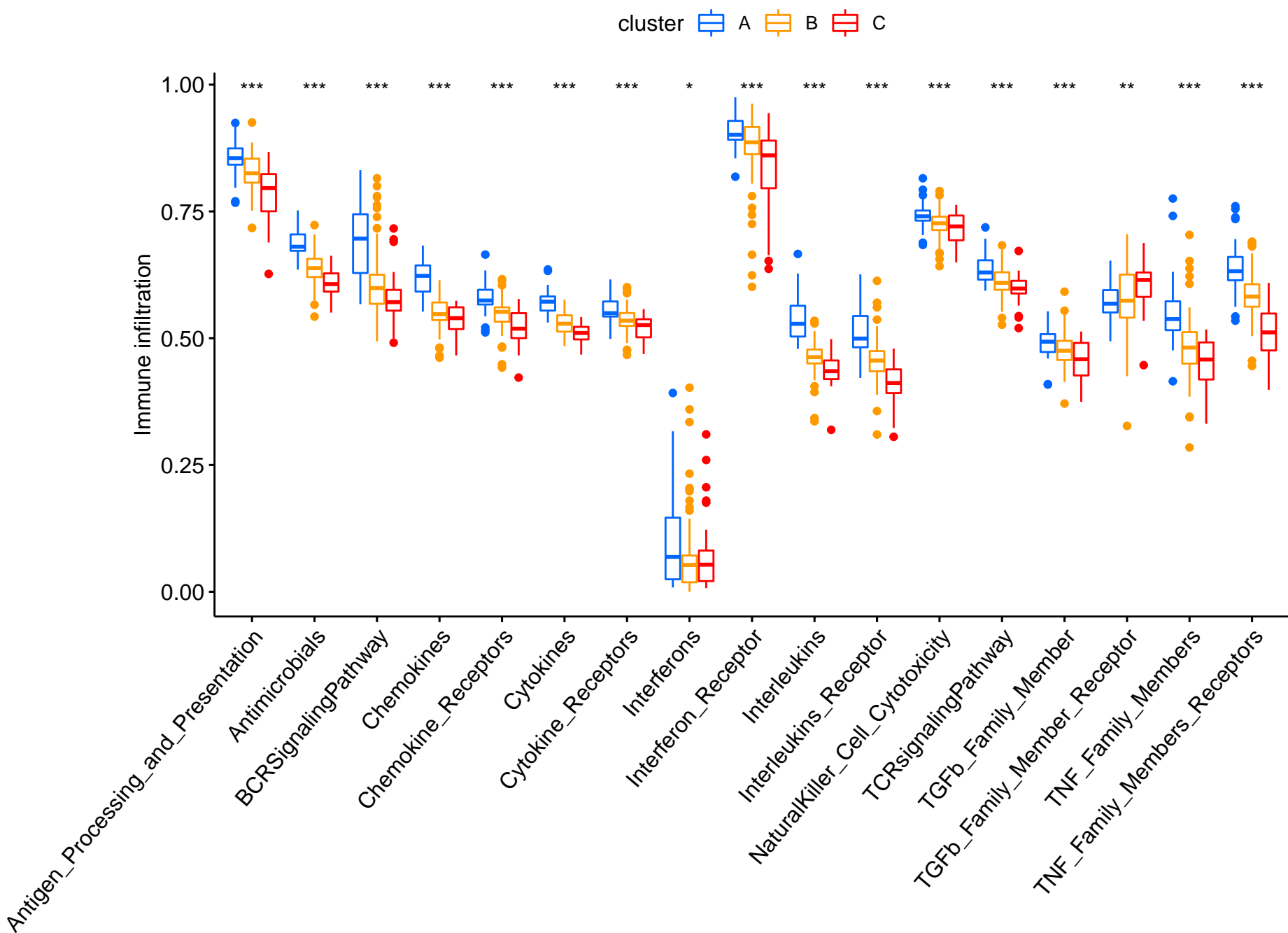

Supplement: Supplemental Information 6 [file peerj-11-15615-s006.zip › Figure 3/Figure 3F.pdf]

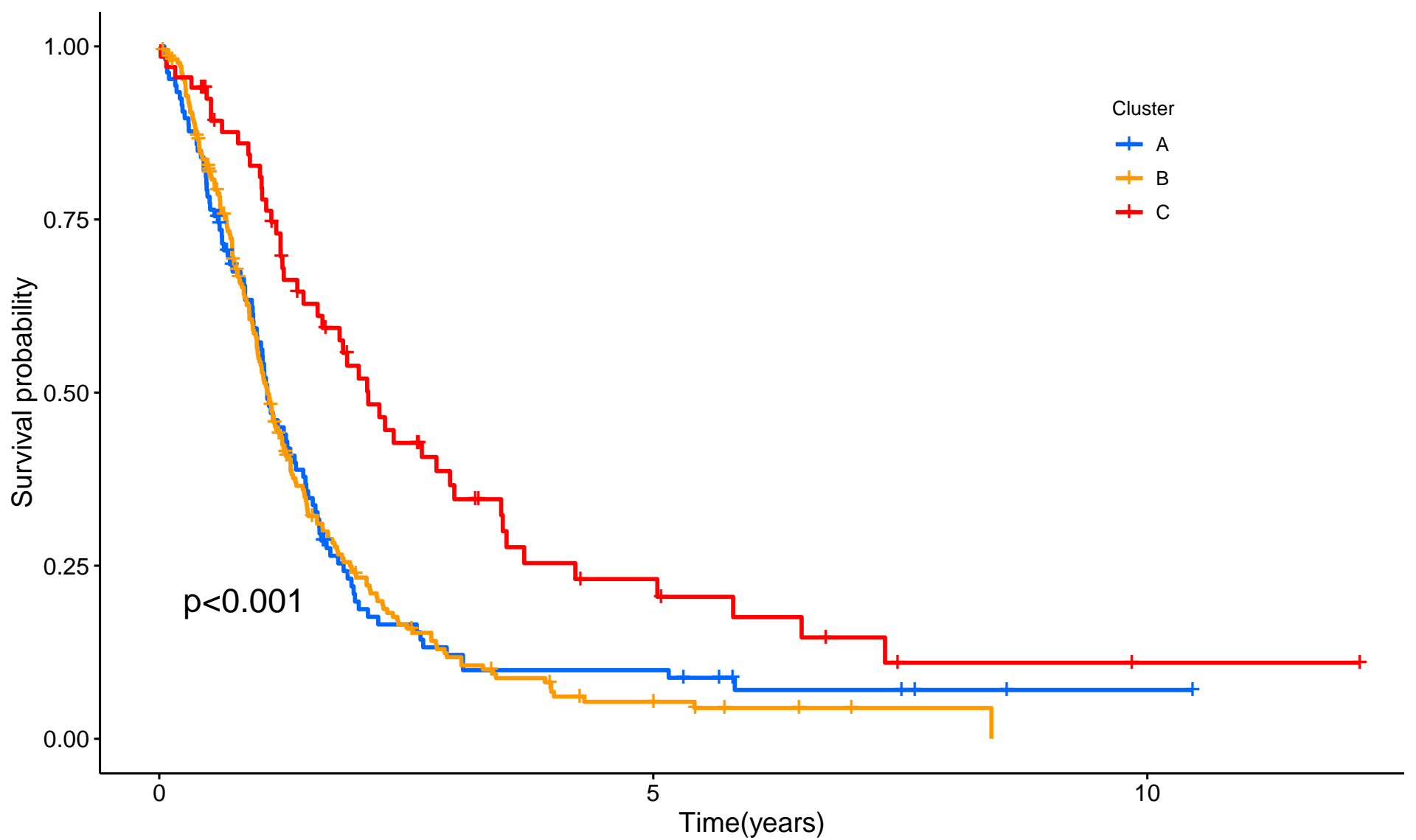

| Number at risk |     |   |    |
|----------------|-----|---|----|
| Cluster        | 0   | 5 | 10 |
| A              | 106 | 9 | 1  |
| B              | 212 | 7 | 0  |
| C              | 67  | 9 | 1  |

Supplement: Supplemental Information 6 [file peerj-11-15615-s006.zip › Figure 3/Figure 3B.pdf]

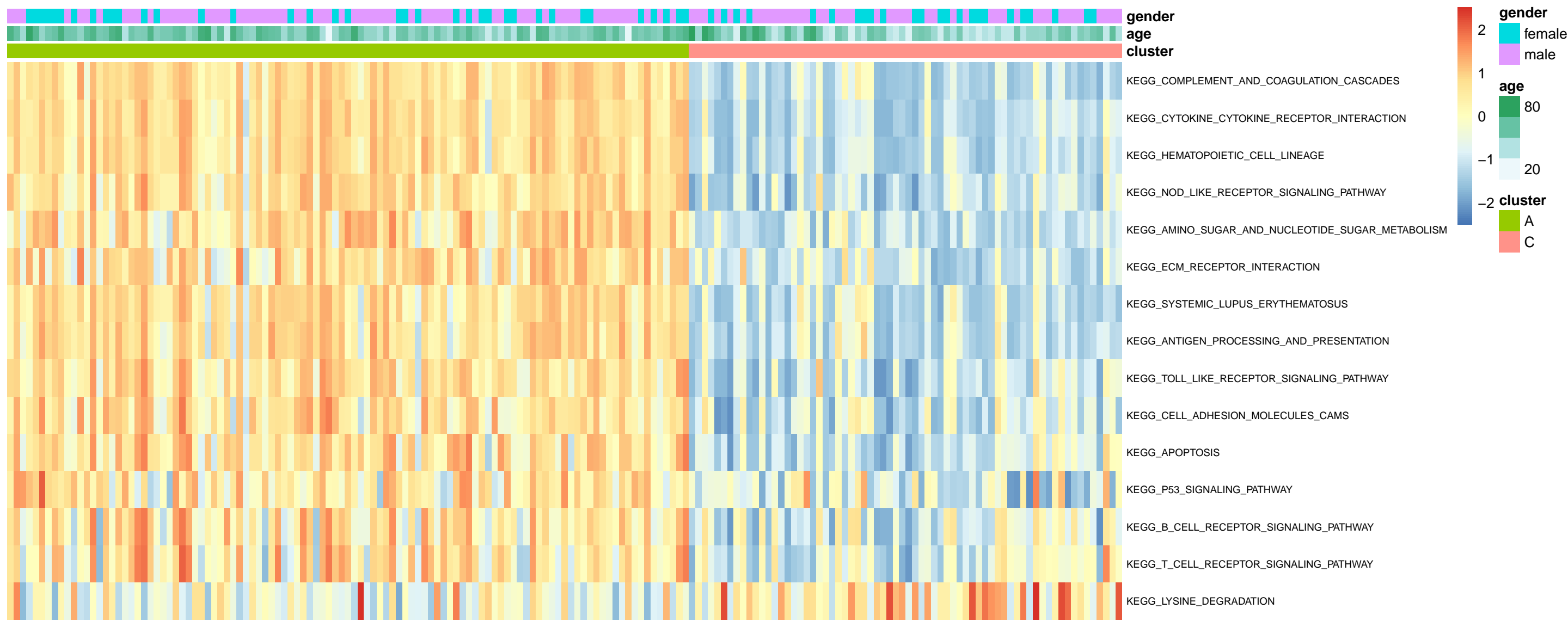

Supplement: Supplemental Information 6 [file peerj-11-15615-s006.zip › Figure 3/Figure 3C.pdf]

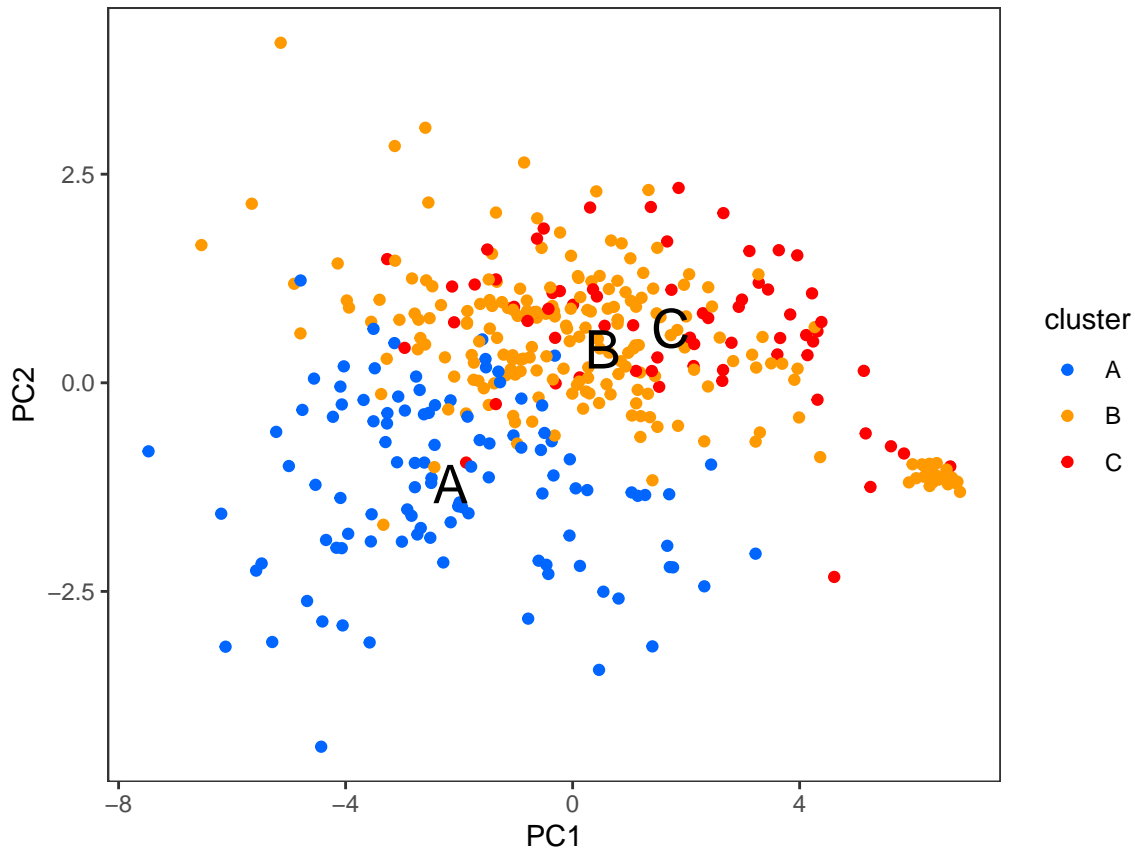

Supplement: Supplemental Information 6 [file peerj-11-15615-s006.zip › Figure 3/Figure 3A.pdf]

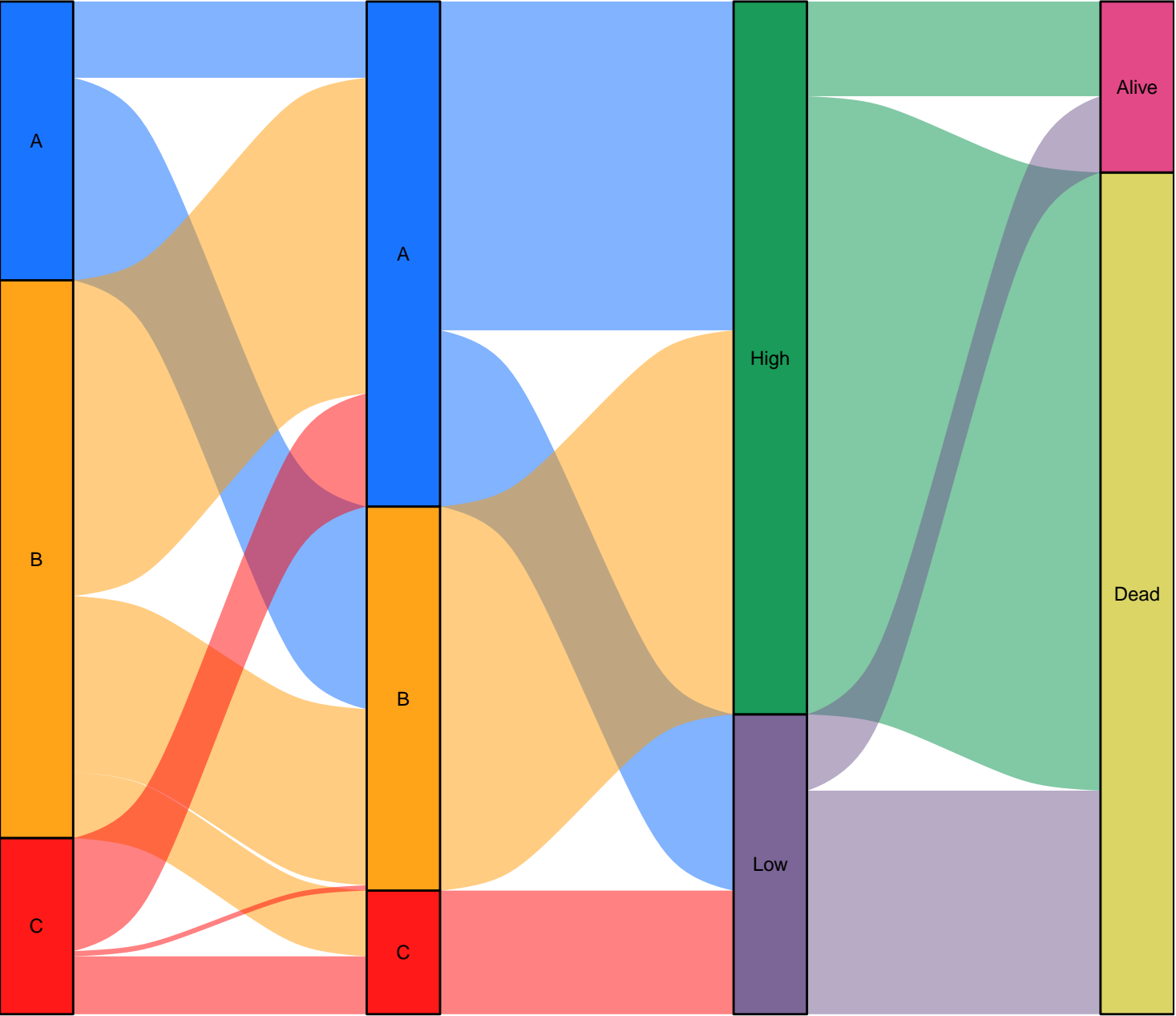

Cluster

geneCluster

Score

fustat

Supplement: Supplemental Information 7 [file peerj-11-15615-s007.zip › Figure 4/Figure 4C.pdf]

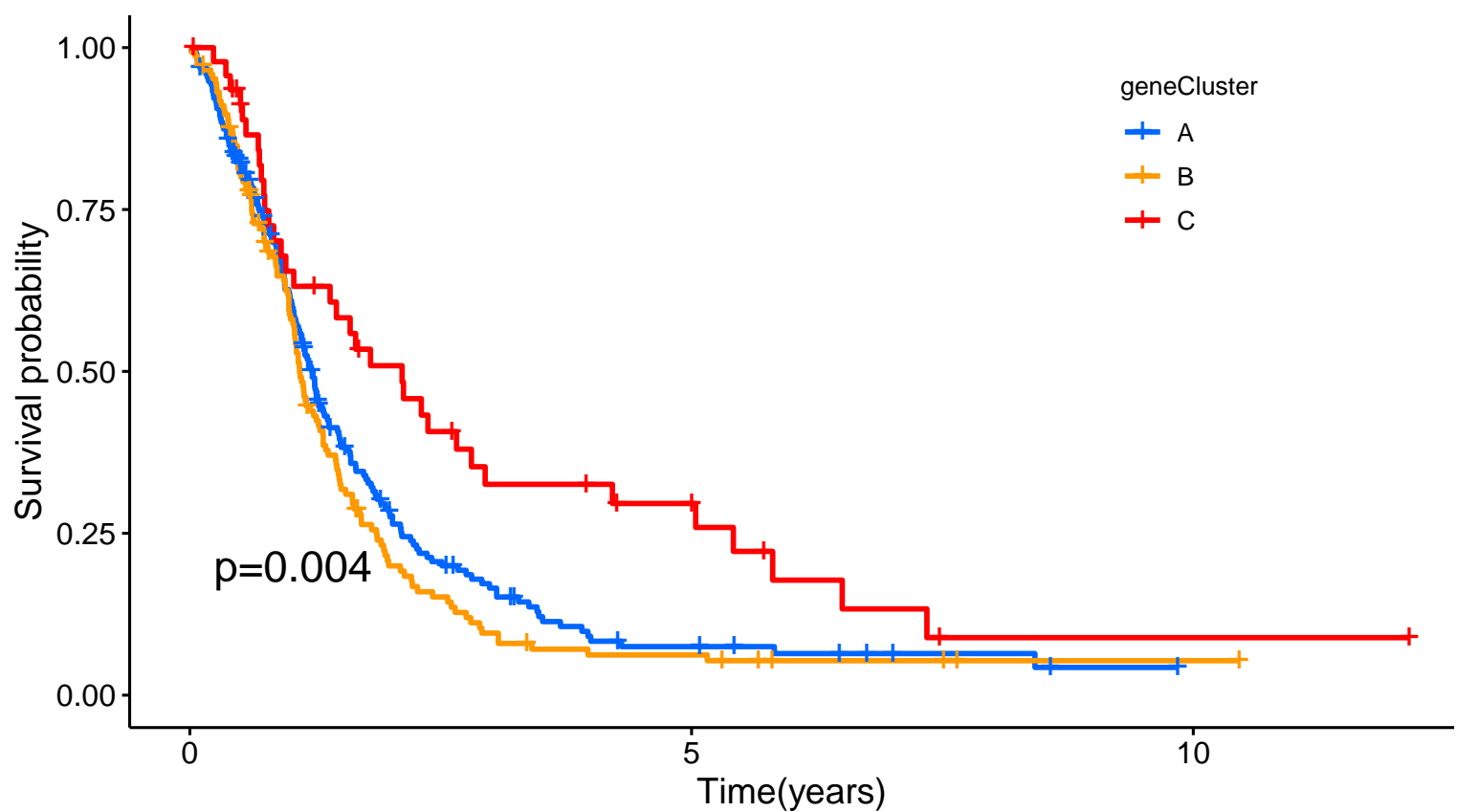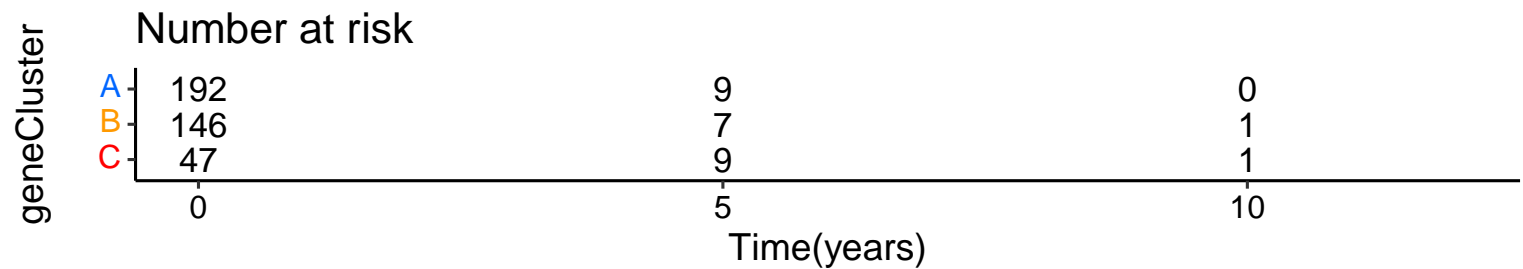

Supplement: Supplemental Information 7 [file peerj-11-15615-s007.zip › Figure 4/Figure 4B.pdf]

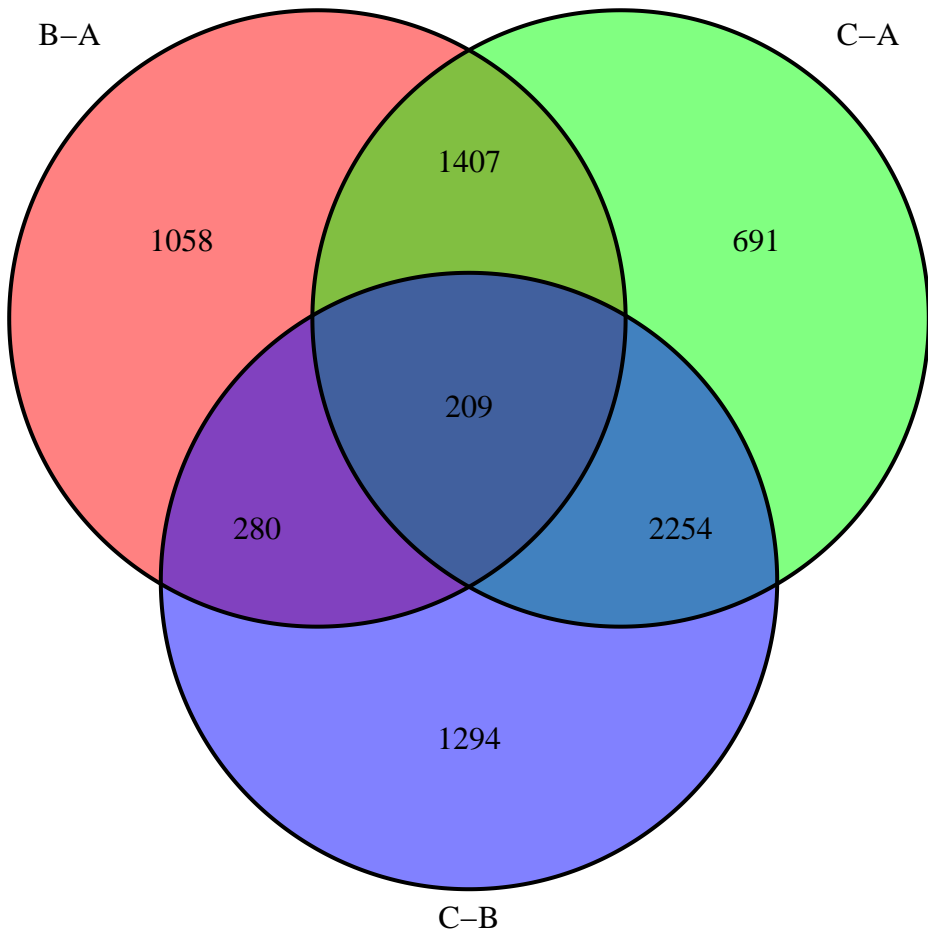

Supplement: Supplemental Information 7 [file peerj-11-15615-s007.zip › Figure 4/Figure 4A.pdf]

geneCluster    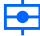 A    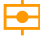 B    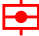 C

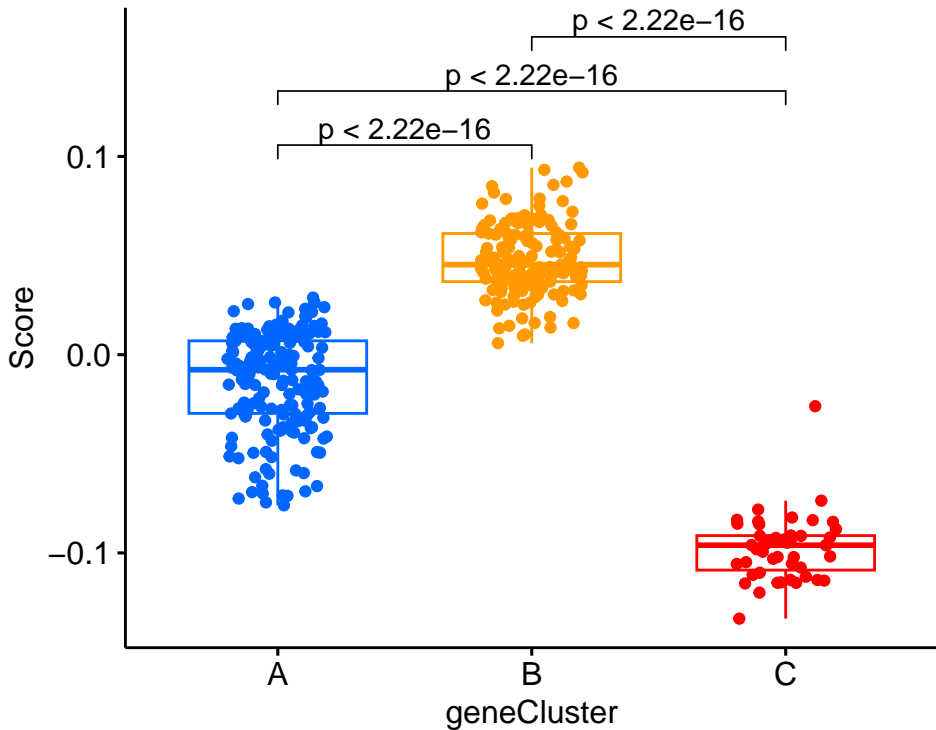

Supplement: Supplemental Information 7 [file peerj-11-15615-s007.zip › Figure 4/Figure 4E.pdf]

Cluster 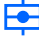 A 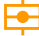 B 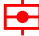 C

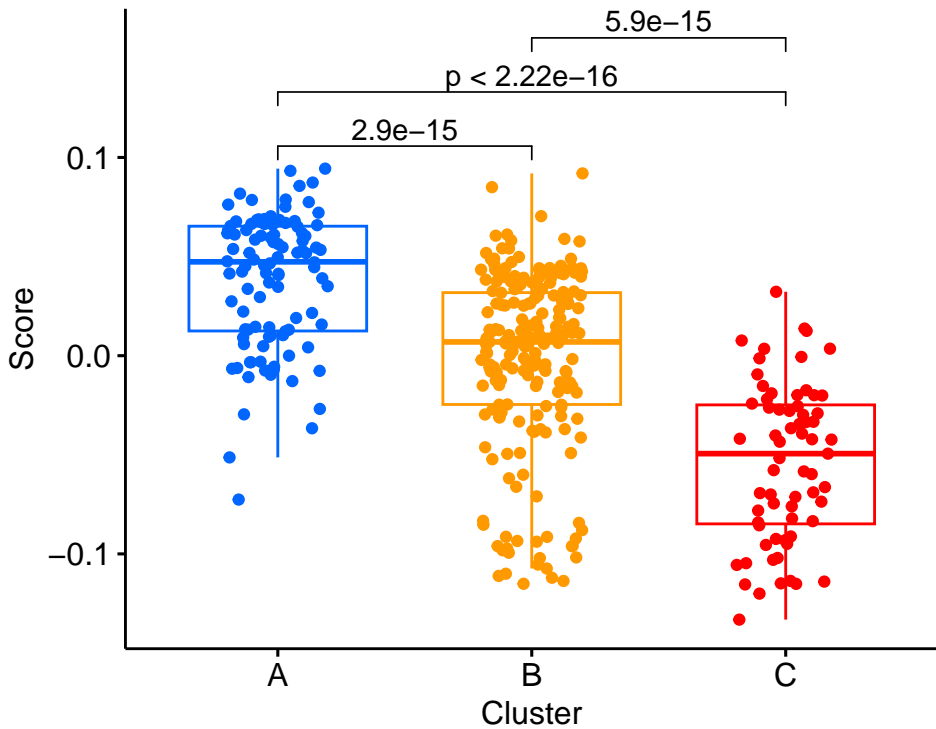

Supplement: Supplemental Information 7 [file peerj-11-15615-s007.zip › Figure 4/Figure 4D.pdf]

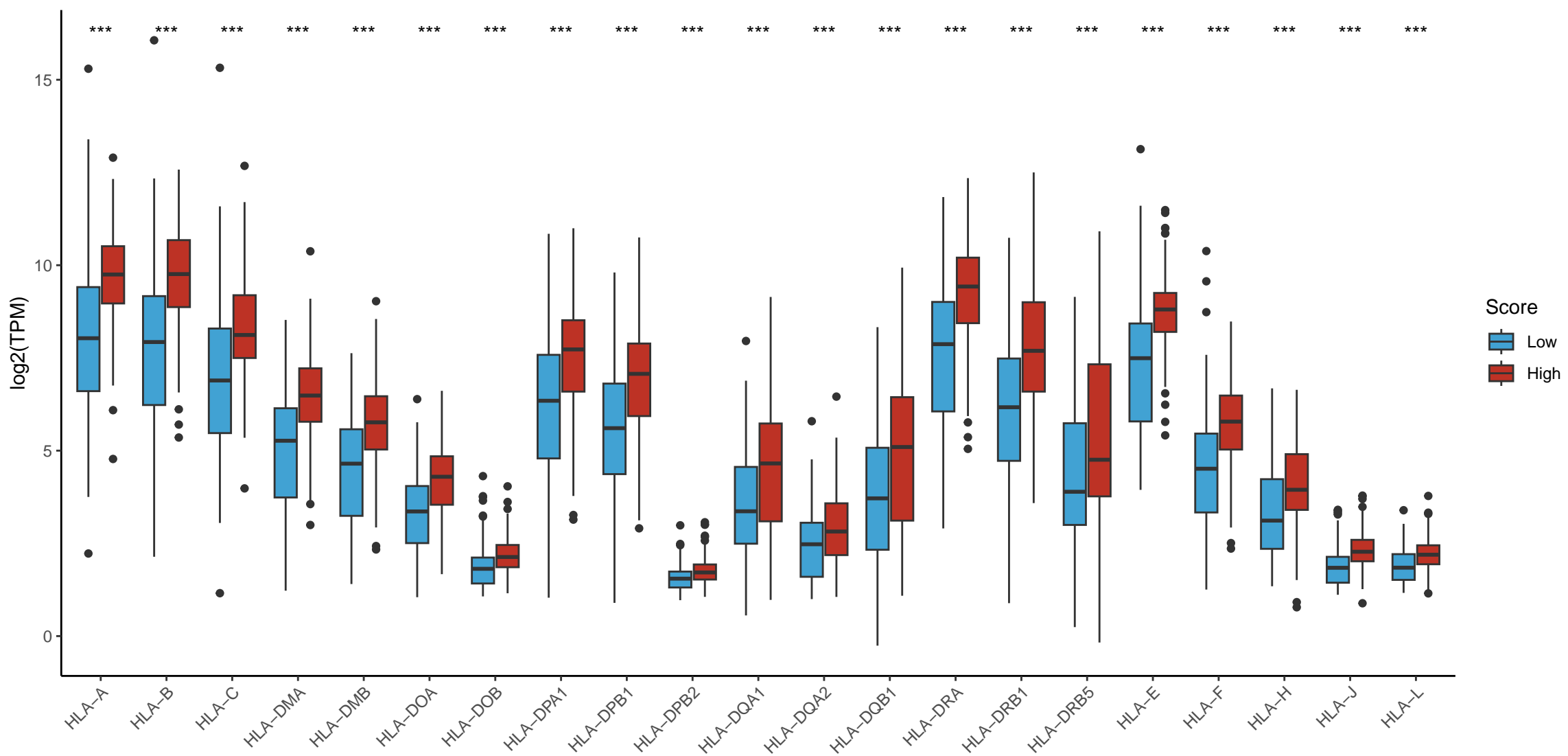

Supplement: Supplemental Information 7 [file peerj-11-15615-s007.zip › Figure 4/Figure 4F.pdf]

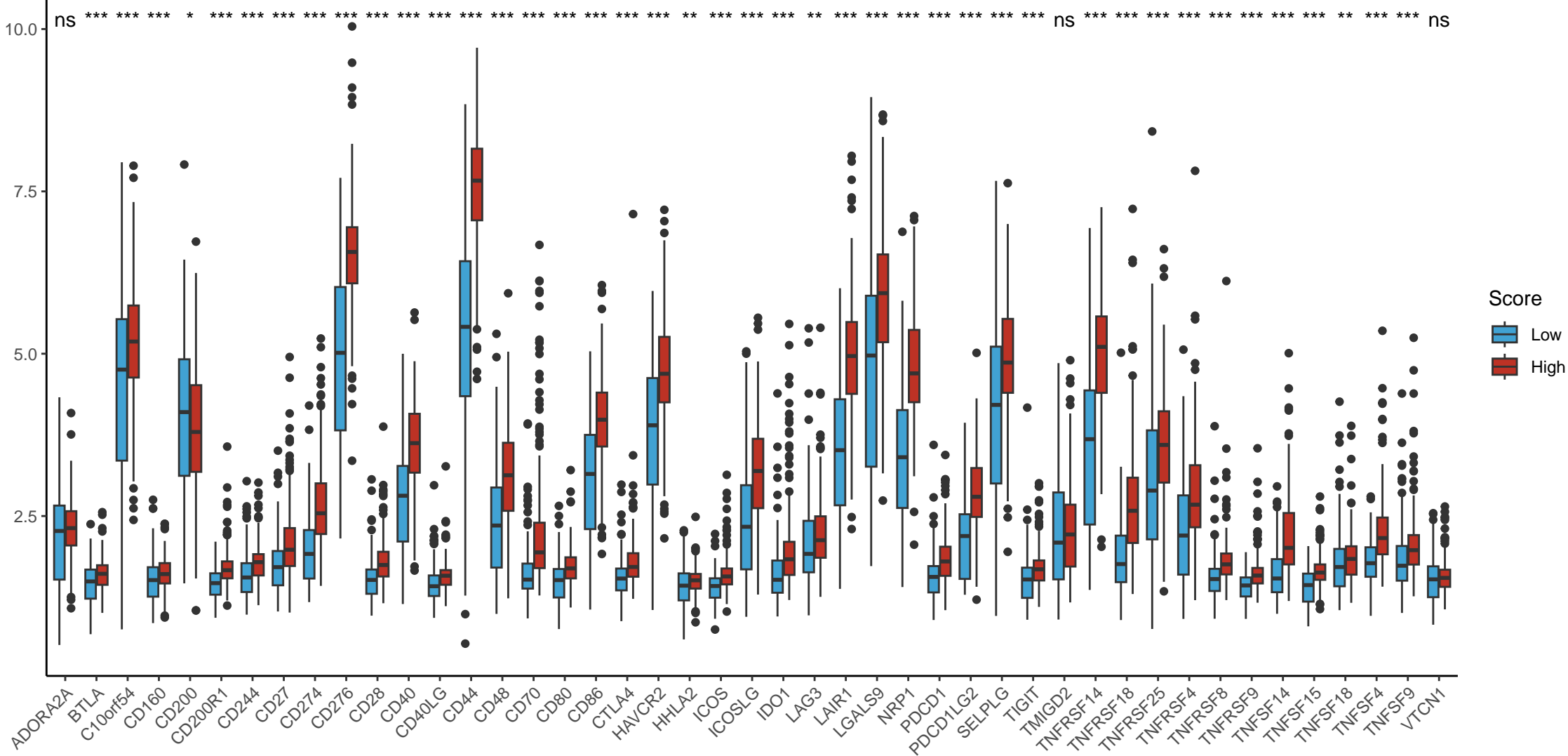

Supplement: Supplemental Information 7 [file peerj-11-15615-s007.zip › Figure 4/Figure 4G.pdf]

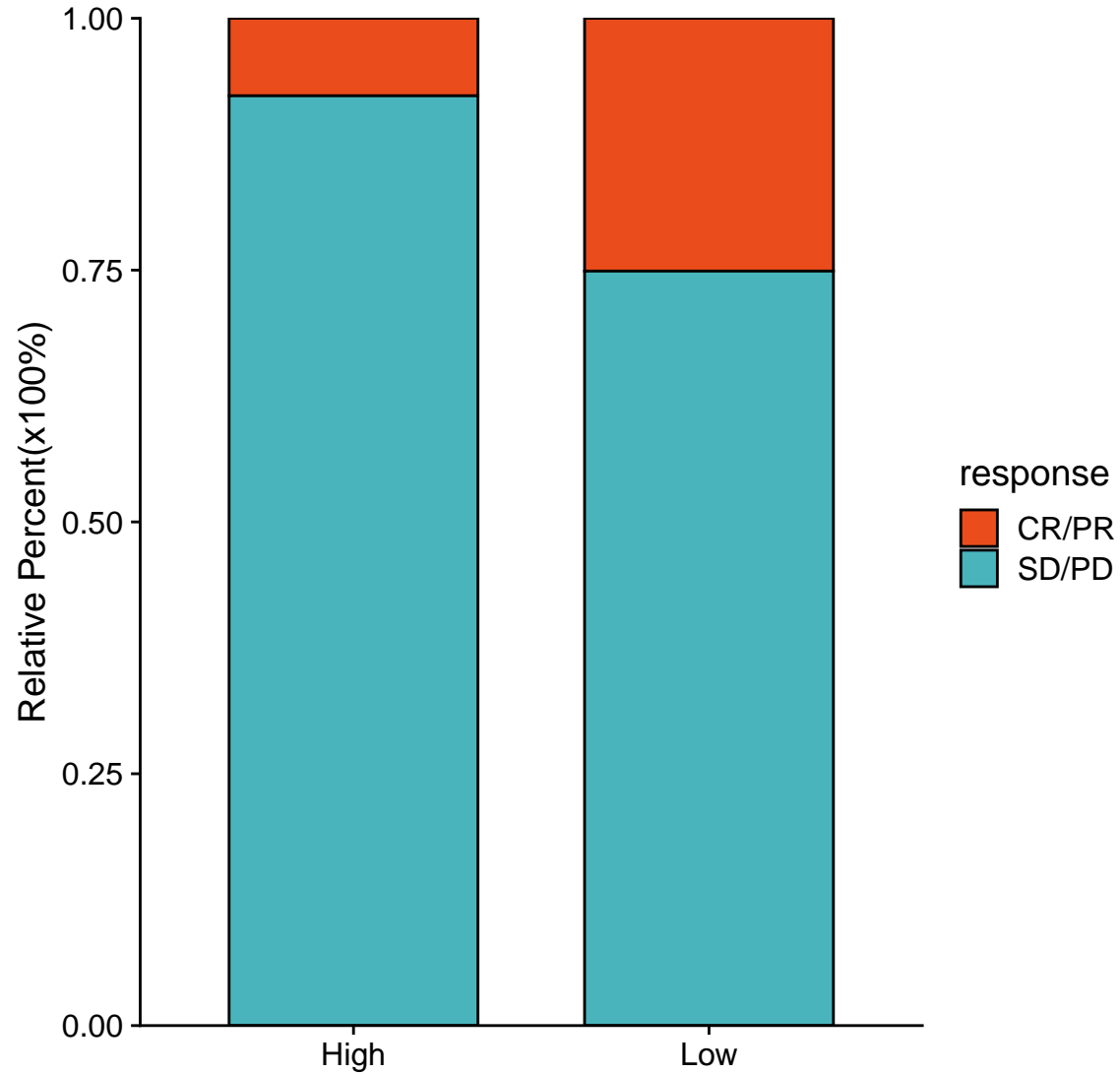

Supplement: Supplemental Information 8 [file peerj-11-15615-s008.zip › Figure 5/Figure 5G.pdf]

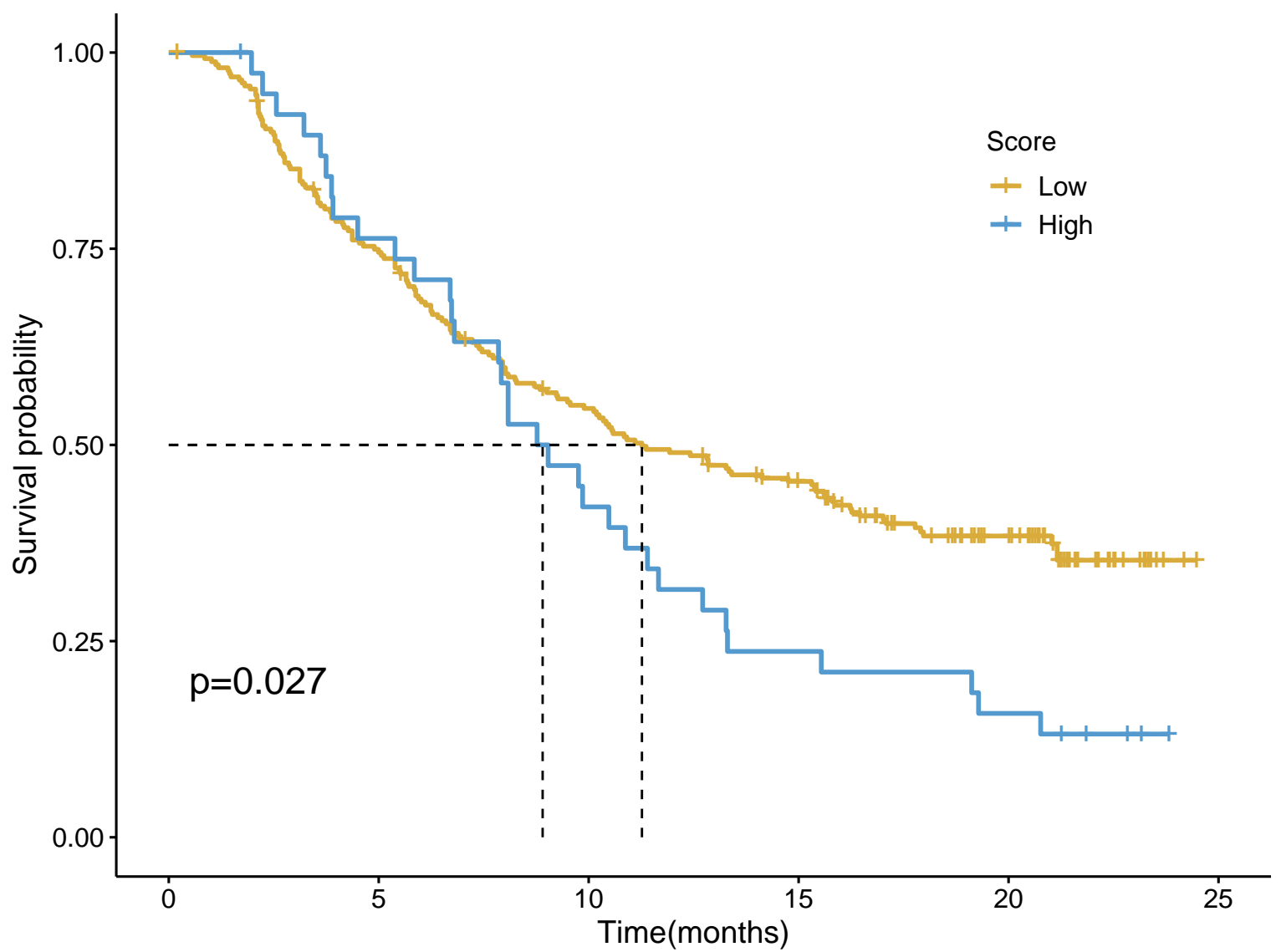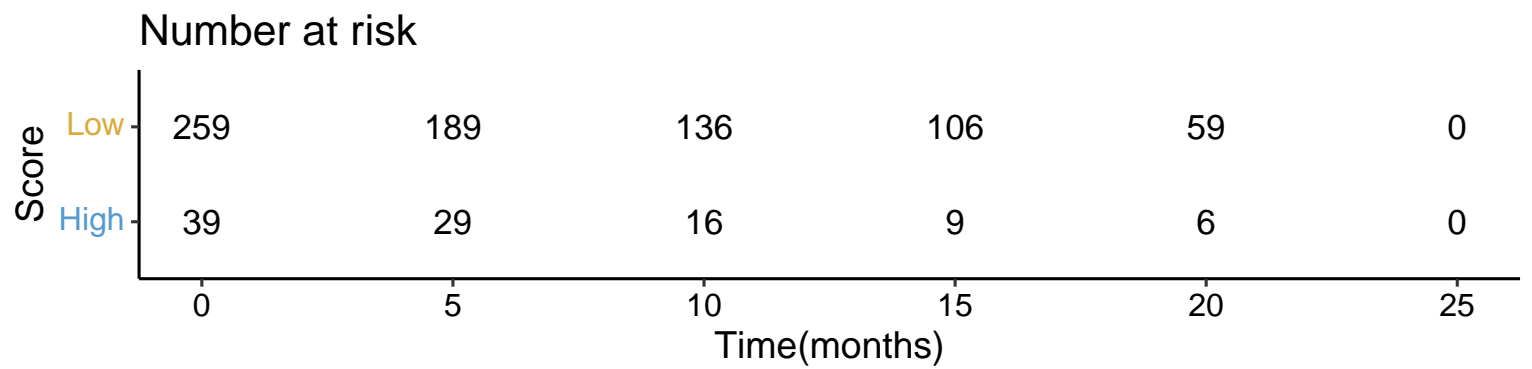

Supplement: Supplemental Information 8 [file peerj-11-15615-s008.zip › Figure 5/Figure 5F.pdf]

BX.795

Low score

High score

p = 0.0025

0

1

2

3

4

IC50

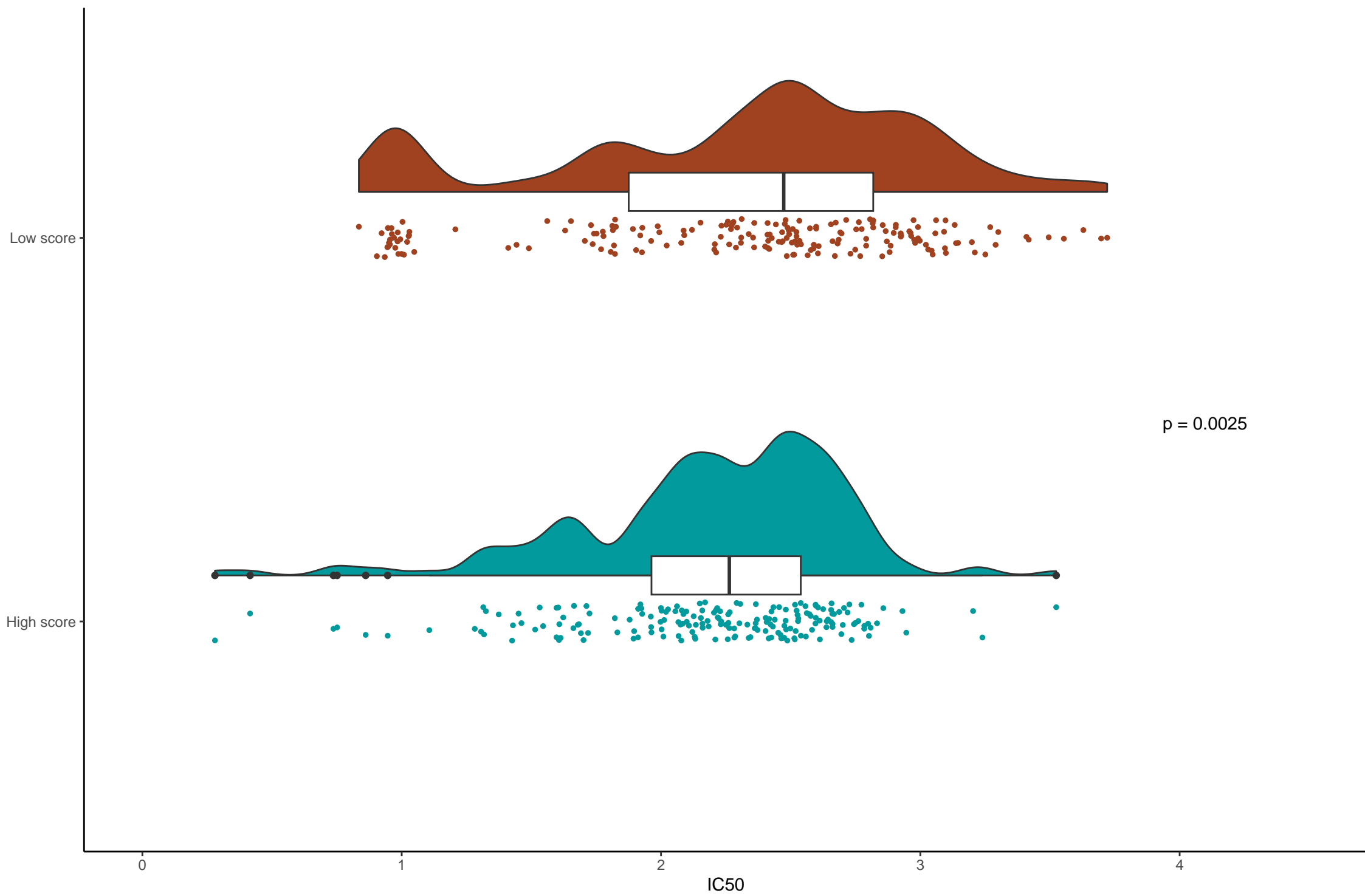

Supplement: Supplemental Information 8 [file peerj-11-15615-s008.zip › Figure 5/Figure 5D.pdf]

BAY.61.3606

Low score

High score

p = 0.0034

IC50

1

2

3

4

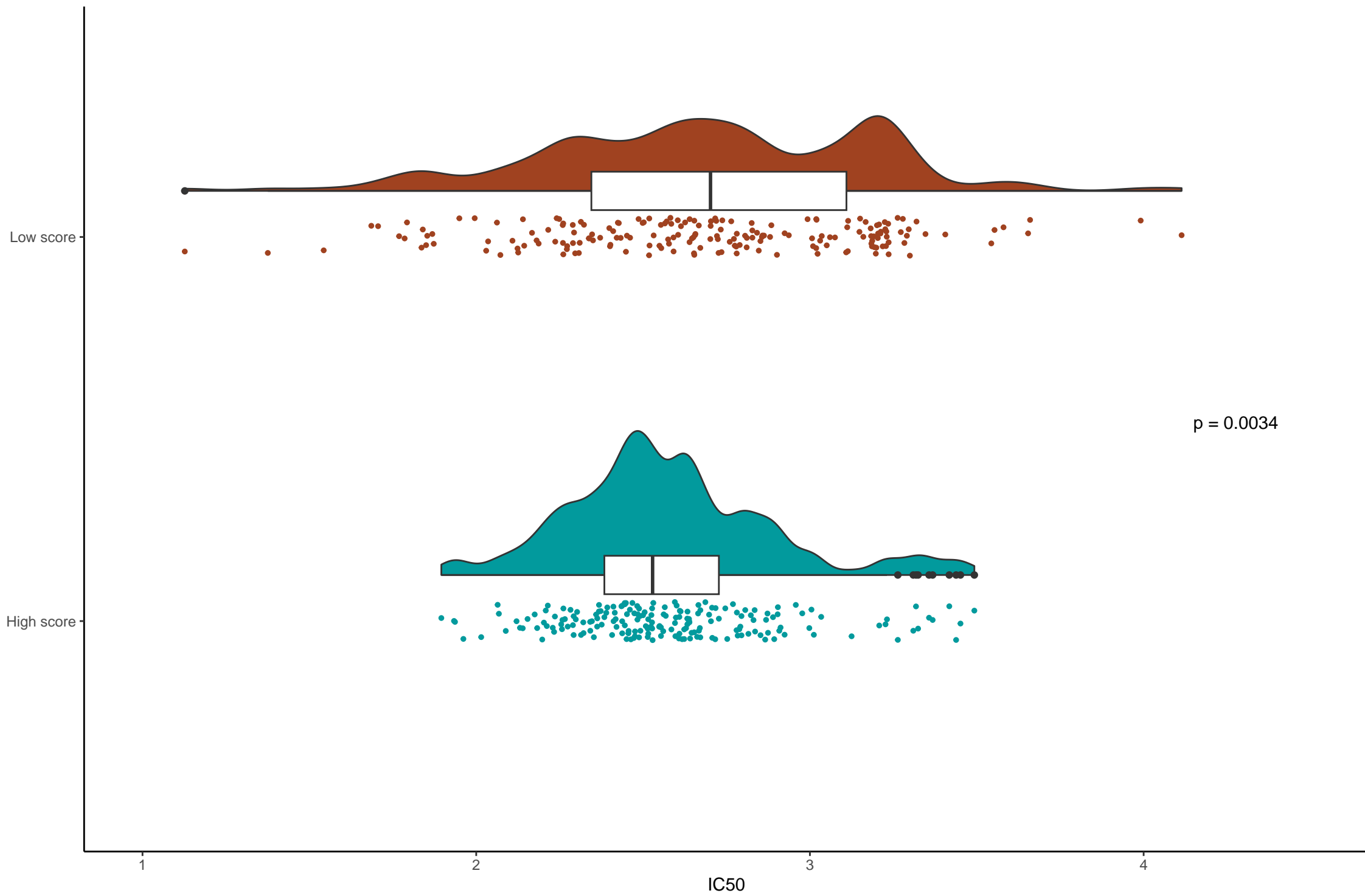

Supplement: Supplemental Information 8 [file peerj-11-15615-s008.zip › Figure 5/Figure 5E.pdf]

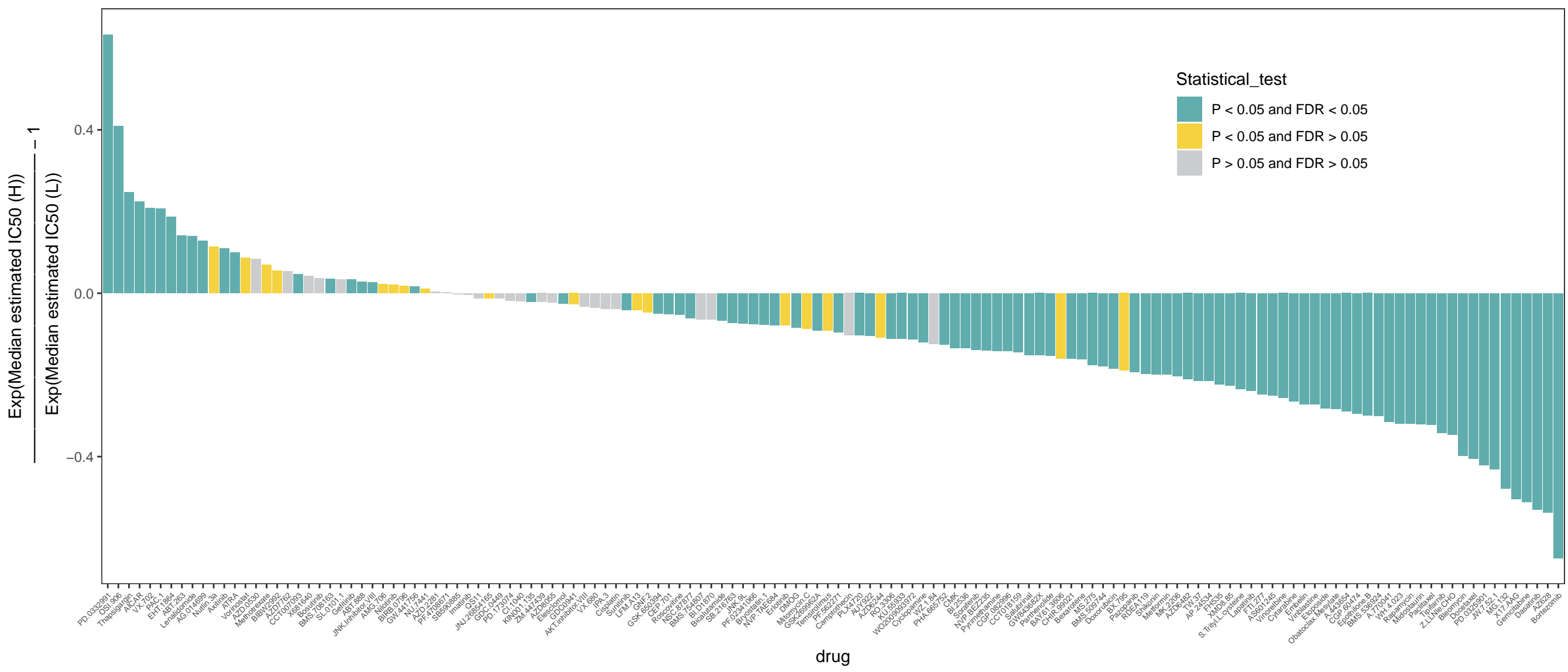

Supplement: Supplemental Information 8 [file peerj-11-15615-s008.zip › Figure 5/Figure 5A.pdf]

Nutlin.3a

Low score

High score

$p = 0.0045$

2

3

4

IC50

5

6

7

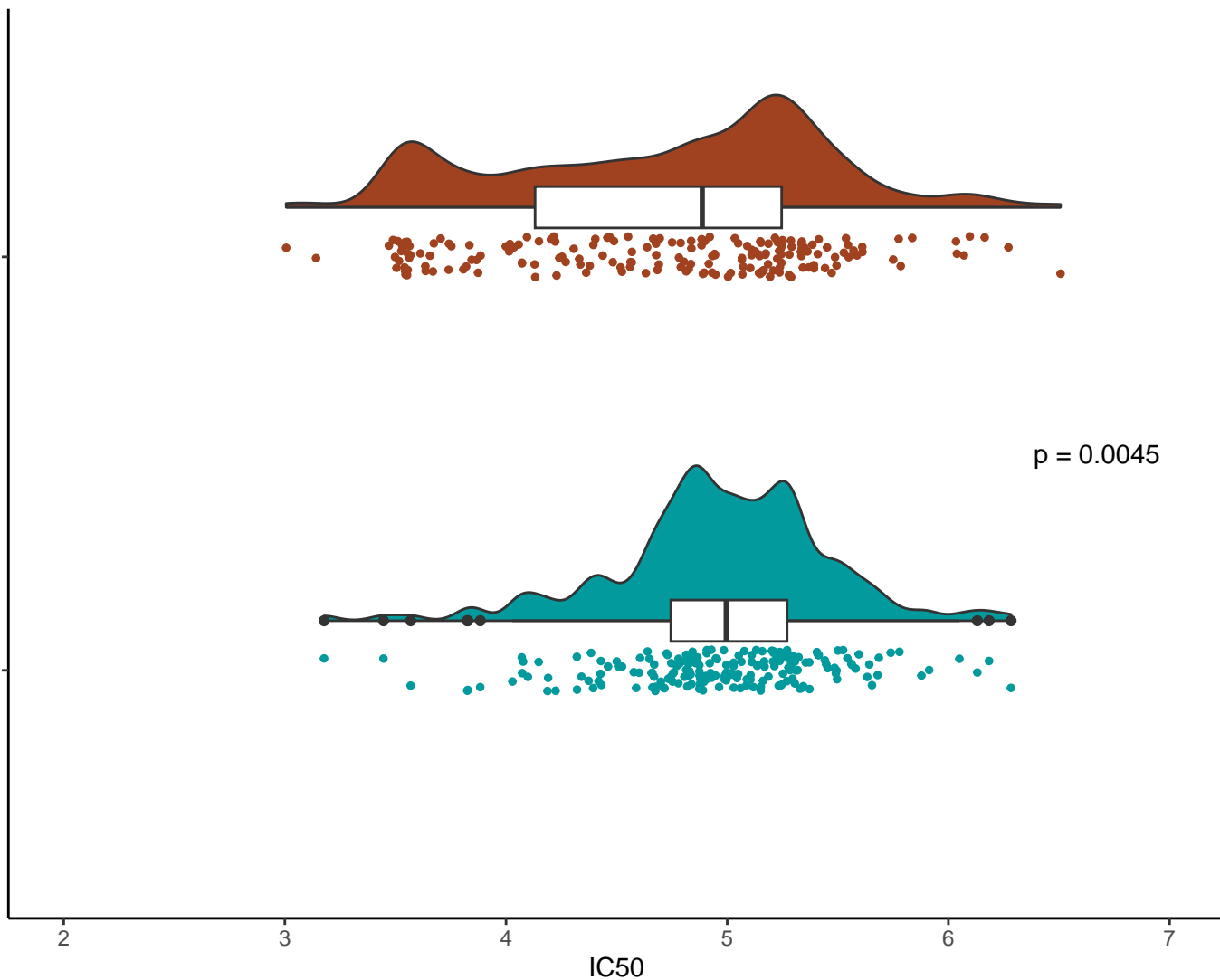

Supplement: Supplemental Information 8 [file peerj-11-15615-s008.zip › Figure 5/Figure 5B.pdf]

# Vorinostat

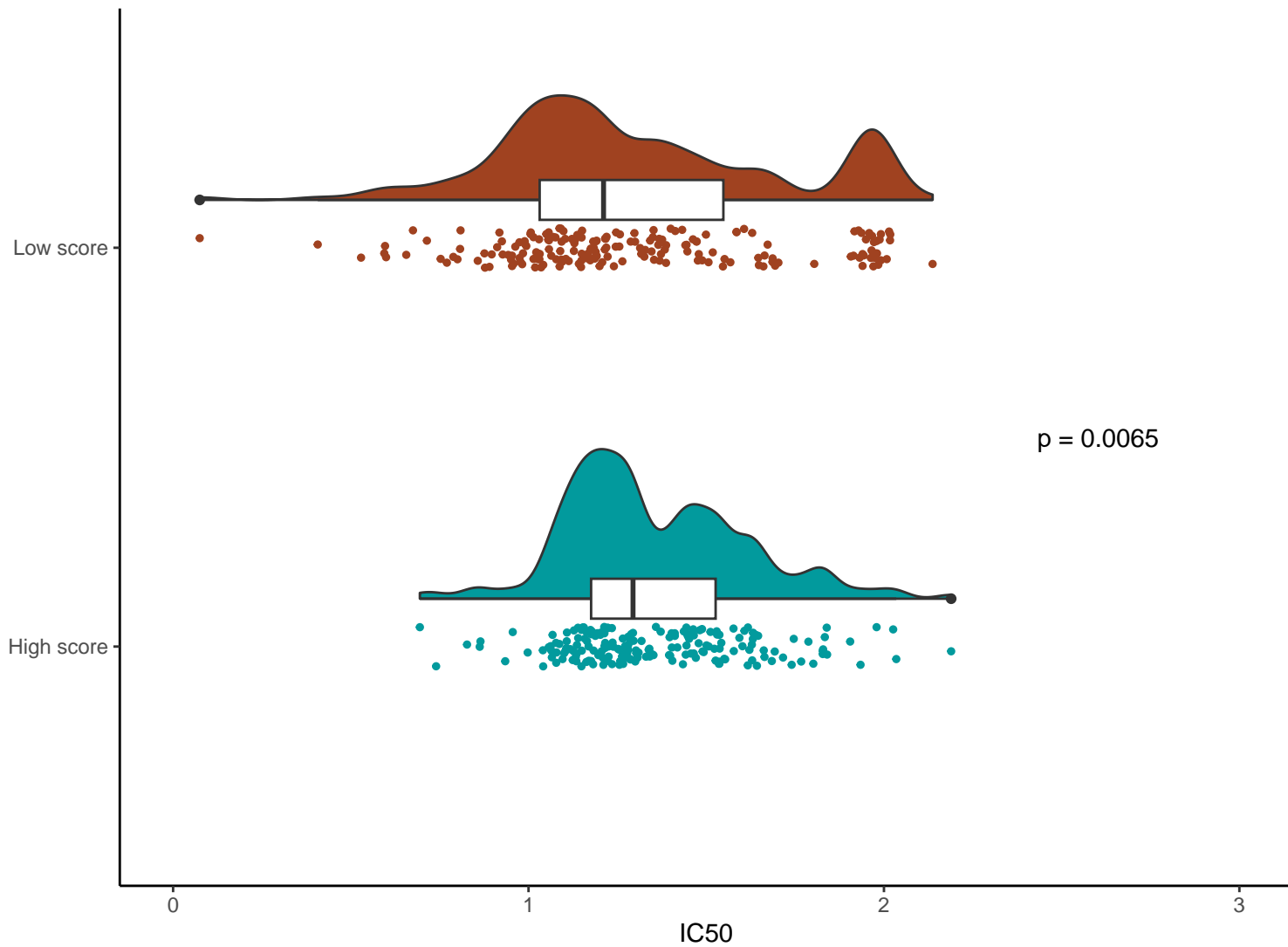

Supplement: Supplemental Information 8 [file peerj-11-15615-s008.zip › Figure 5/Figure 5C.pdf]

Kruskal-Wallis,  $p = 0.033$

CuFescore

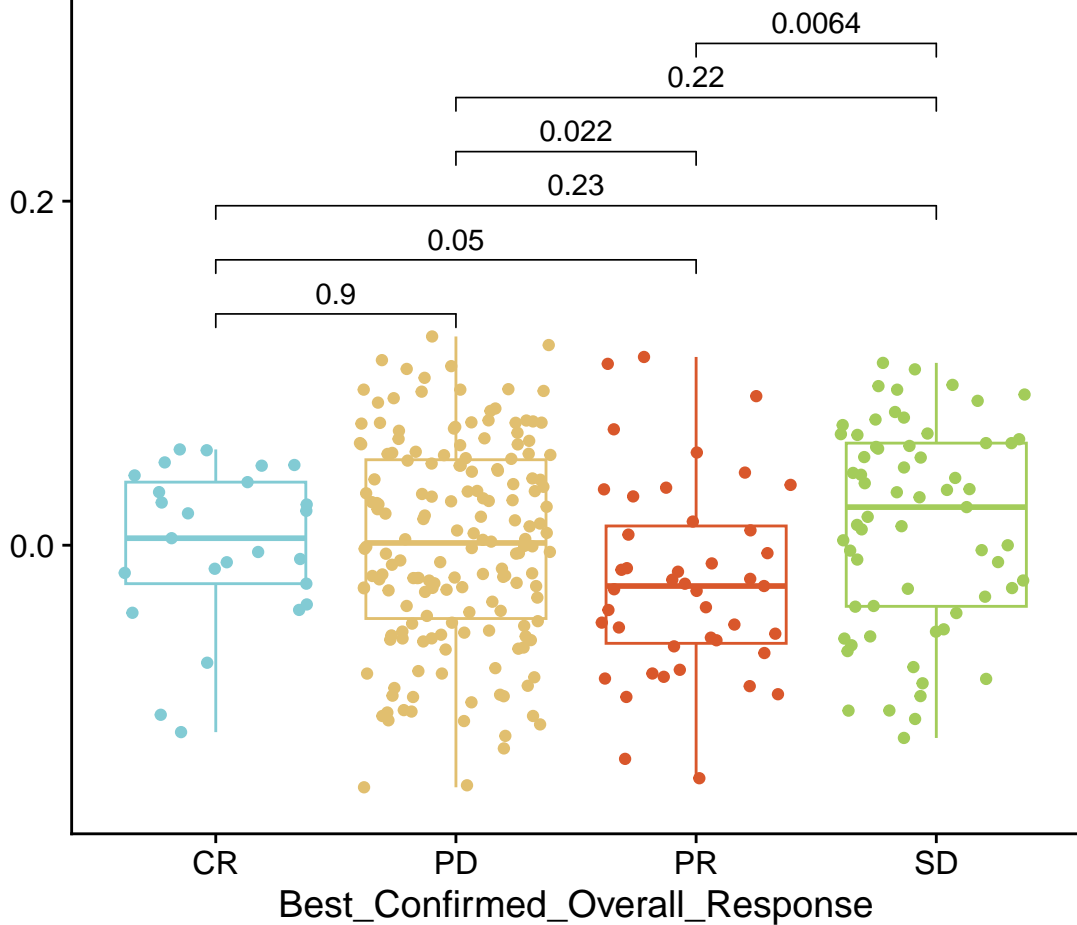

Supplement: Supplemental Information 8 [file peerj-11-15615-s008.zip › Figure 5/Figure 5H.pdf]

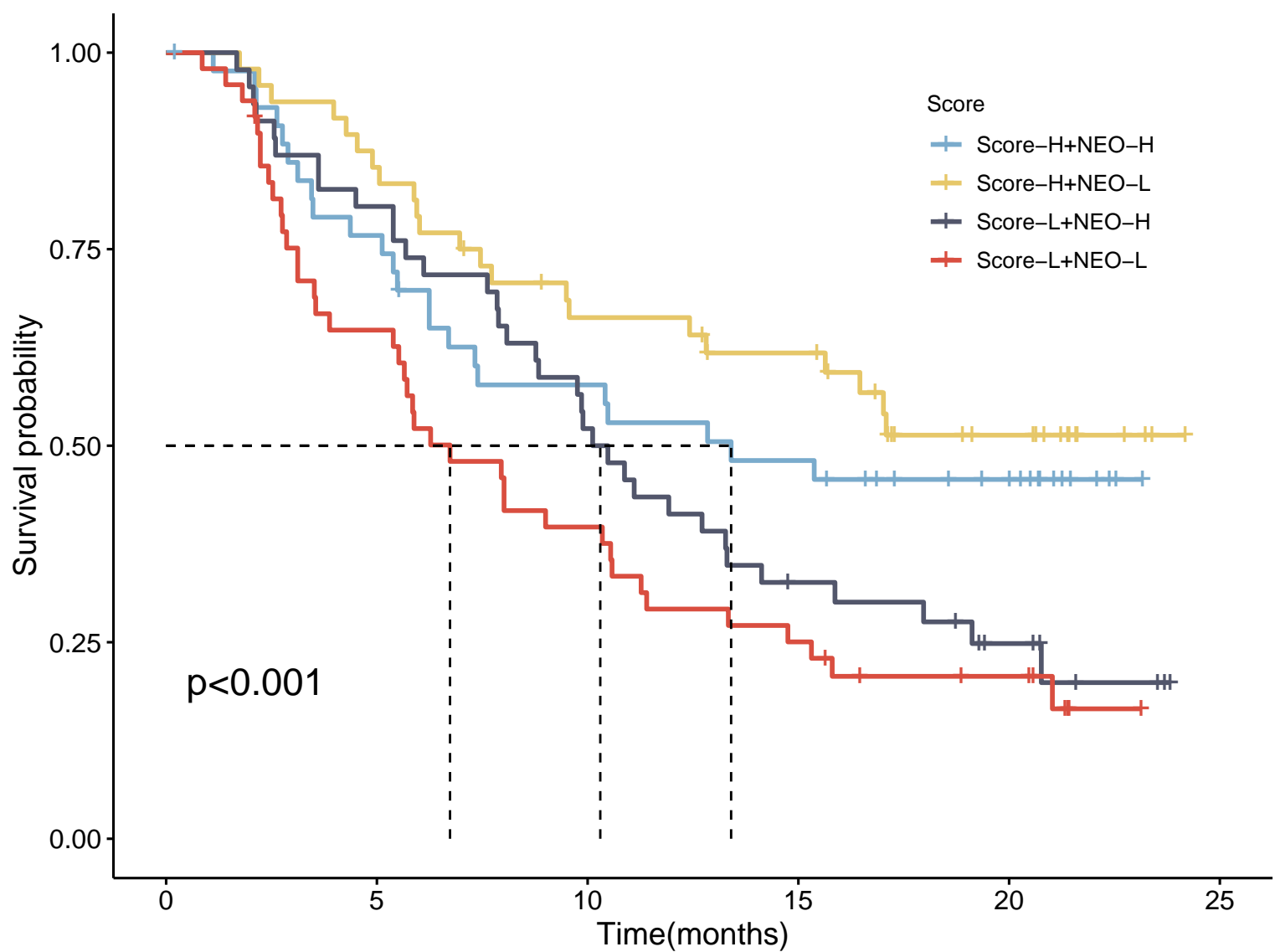

Supplement: Supplemental Information 8 [file peerj-11-15615-s008.zip › Figure 5/Figure 5I.pdf]

Kruskal–Wallis,  $p = 0.00039$

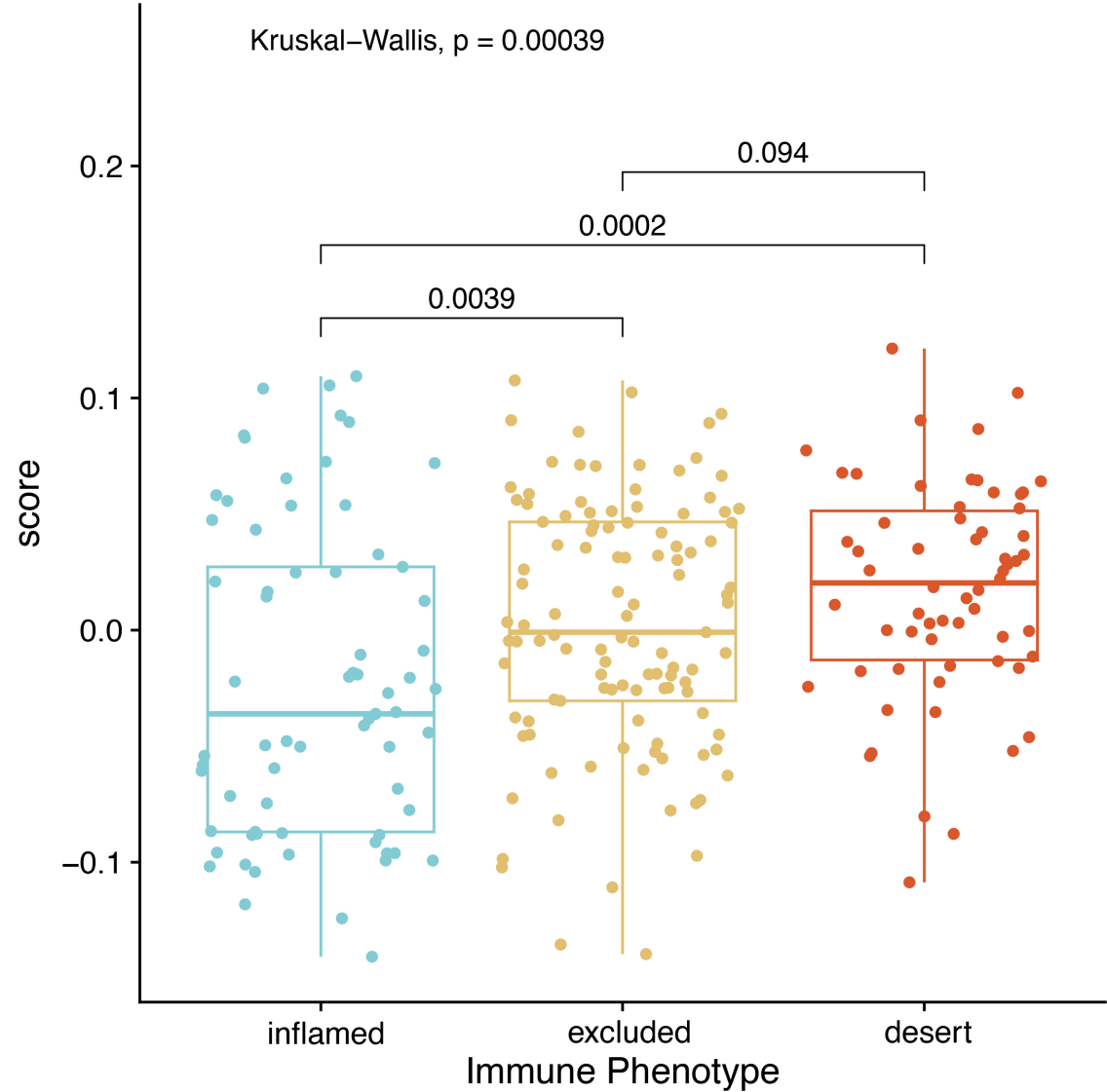

Supplement: Supplemental Information 8 [file peerj-11-15615-s008.zip › Figure 5/Figure 5J.pdf]

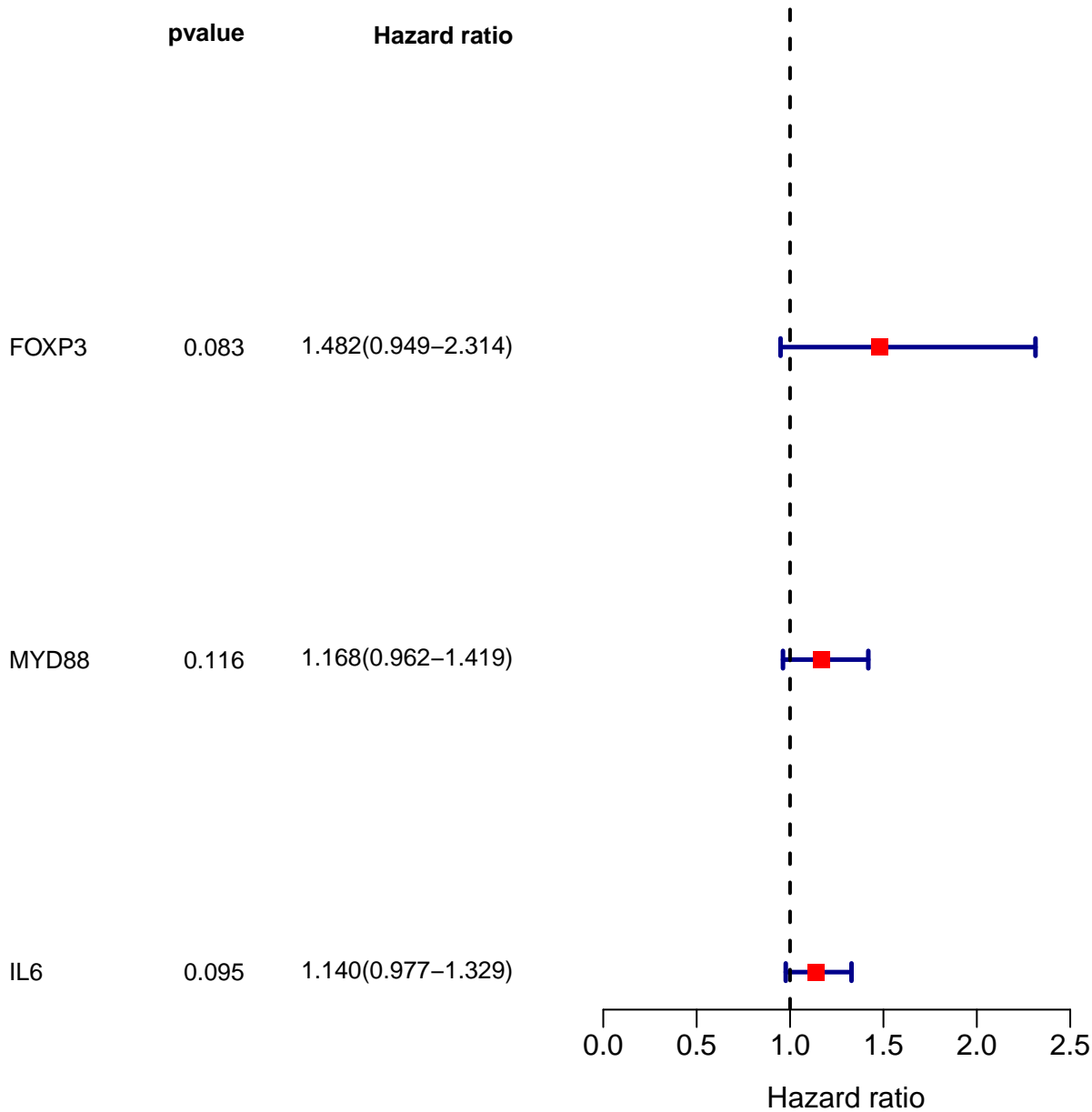

Supplement: Supplemental Information 9 [file peerj-11-15615-s009.zip › Figure 6/Figure 6B.pdf]

Points

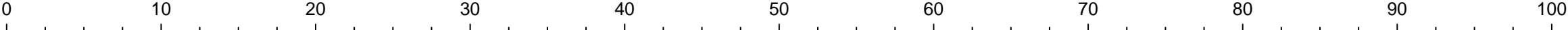

FOXP3

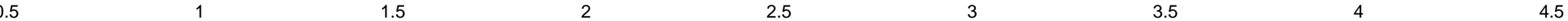

MYD88

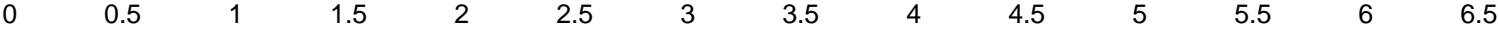

IL6

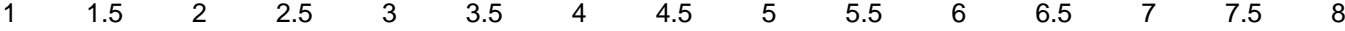

Total Points

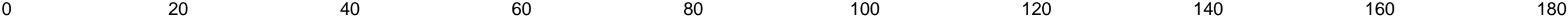

1-year survival

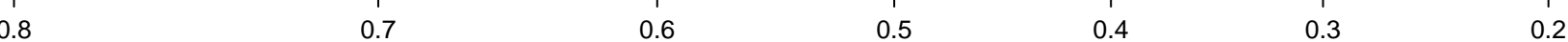

3-year survival

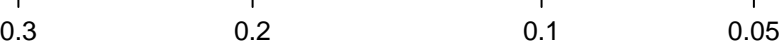

5-year survival

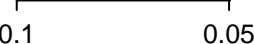

Supplement: Supplemental Information 9 [file peerj-11-15615-s009.zip › Figure 6/Figure 6C.pdf]

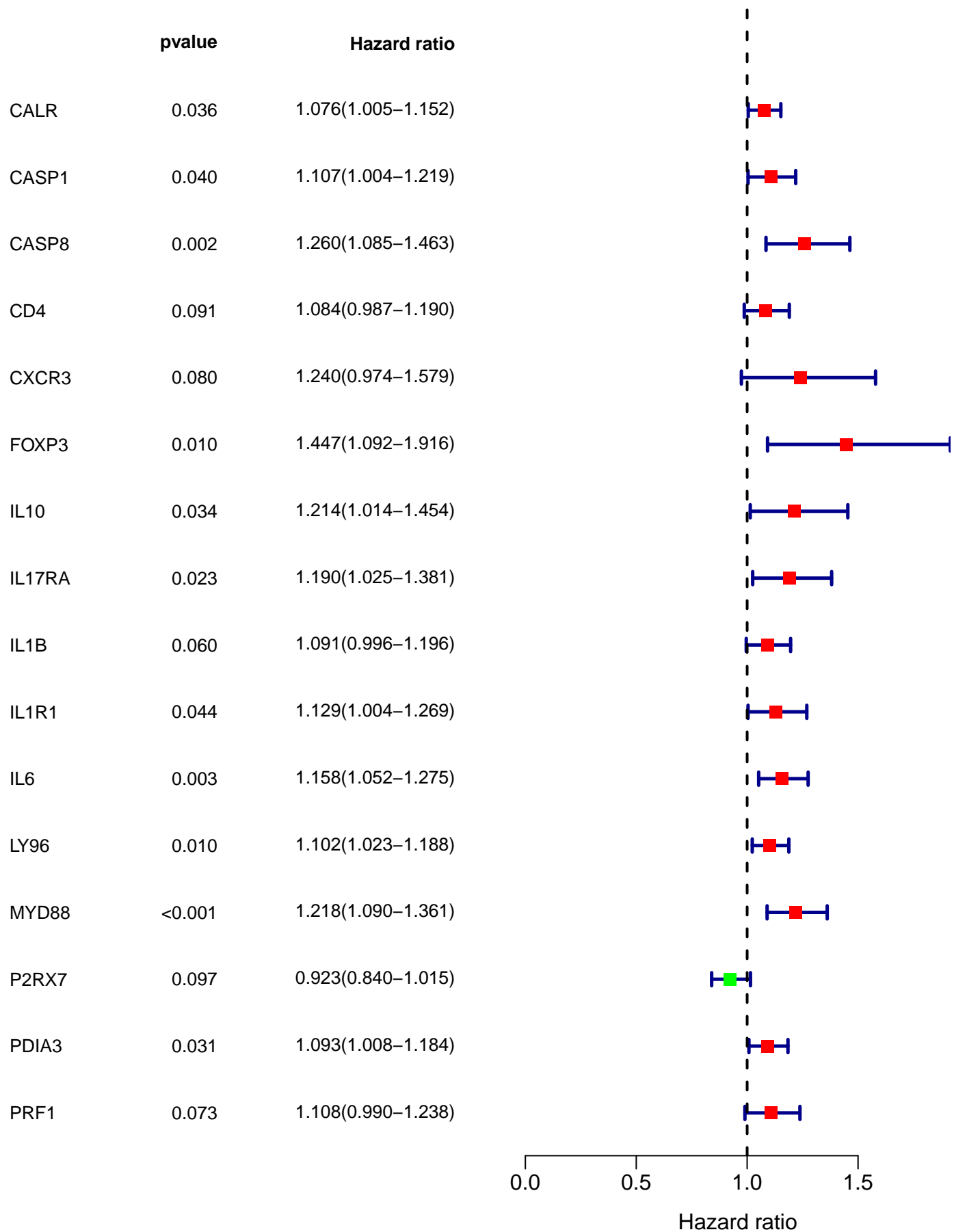

Supplement: Supplemental Information 9 [file peerj-11-15615-s009.zip › Figure 6/Figure 6A.pdf]

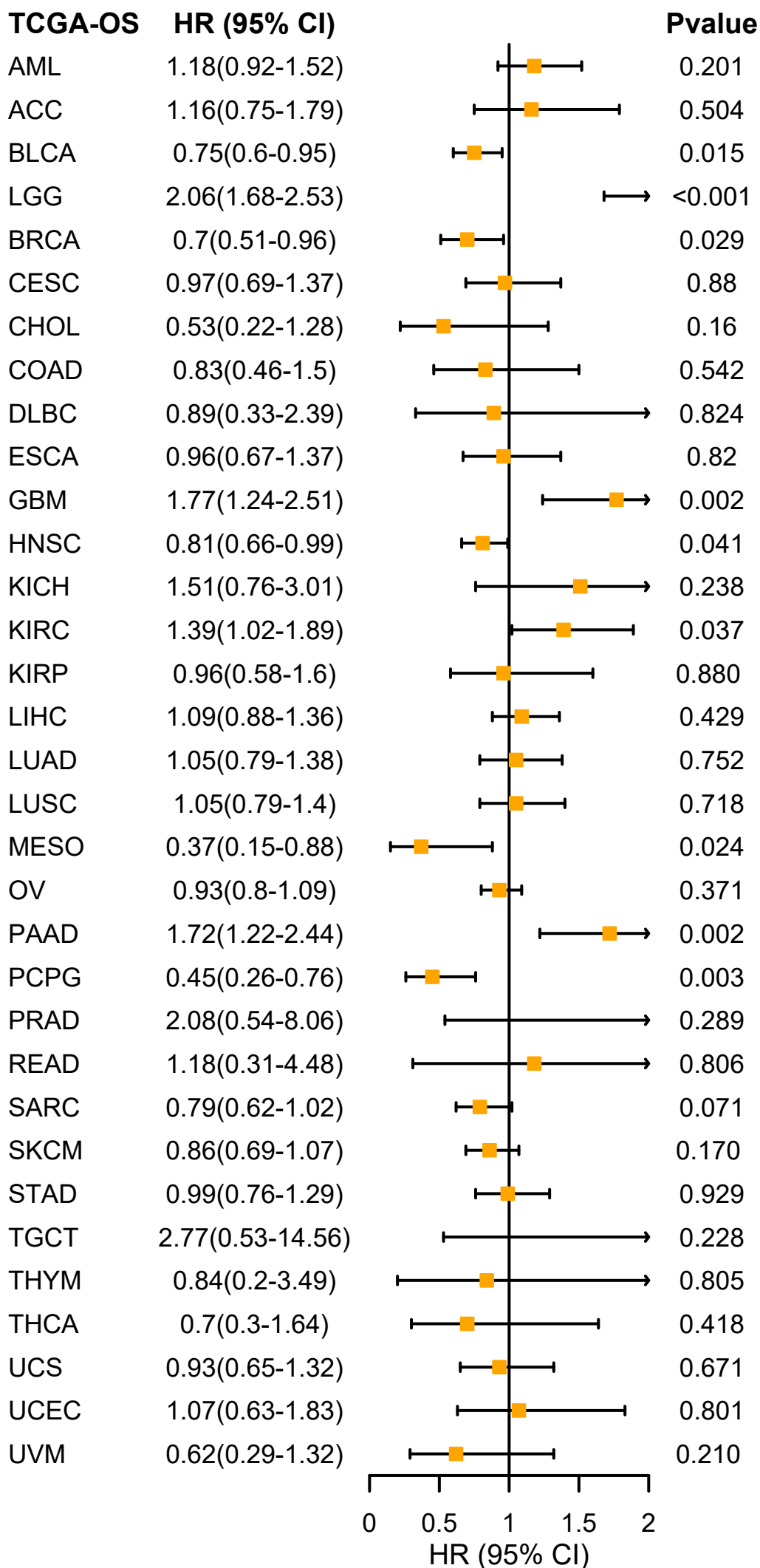

Supplement: Supplemental Information 9 [file peerj-11-15615-s009.zip › Figure 6/Figure 6D.pdf]

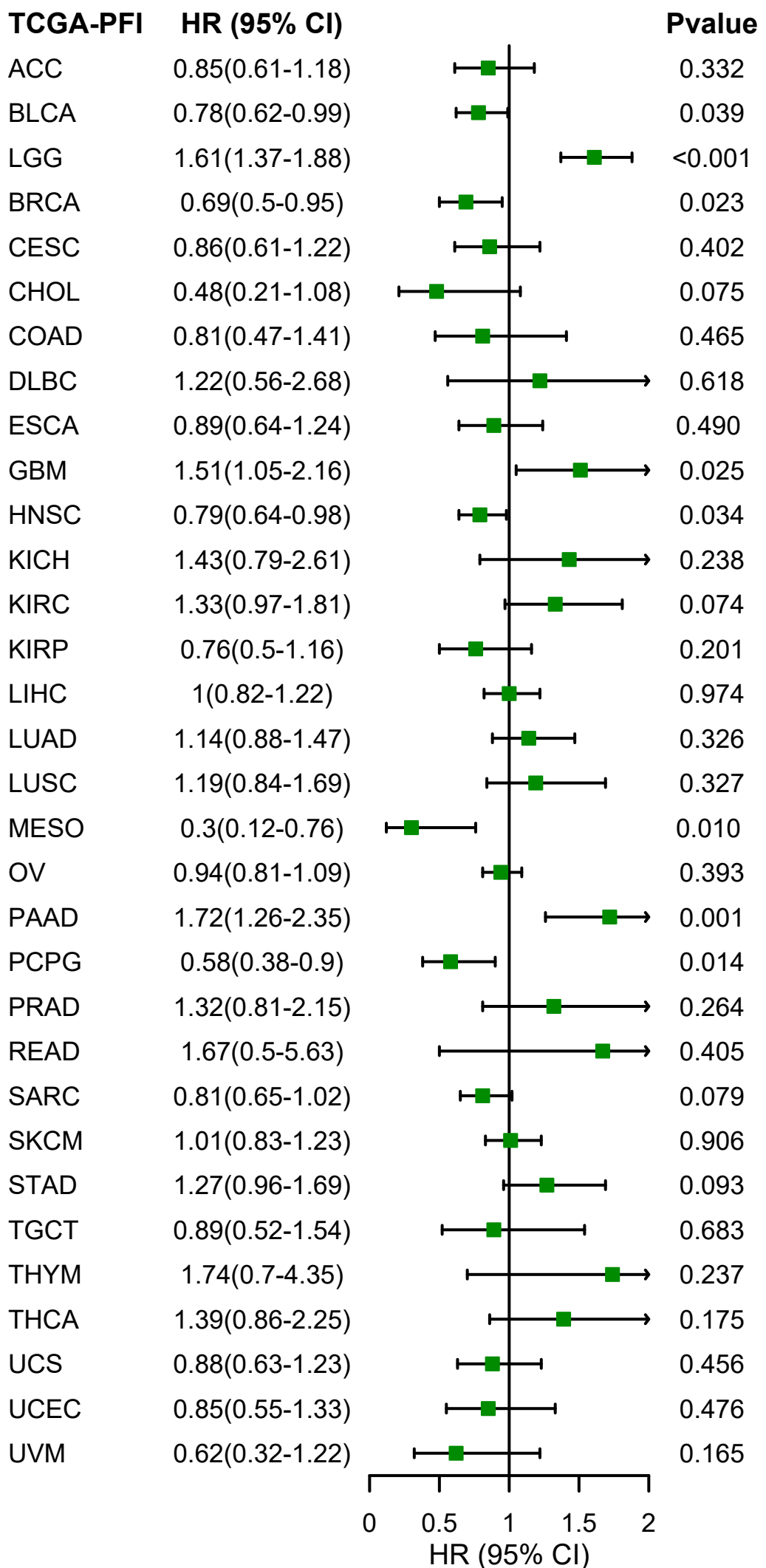

Supplement: Supplemental Information 9 [file peerj-11-15615-s009.zip › Figure 6/Figure 6E.pdf]

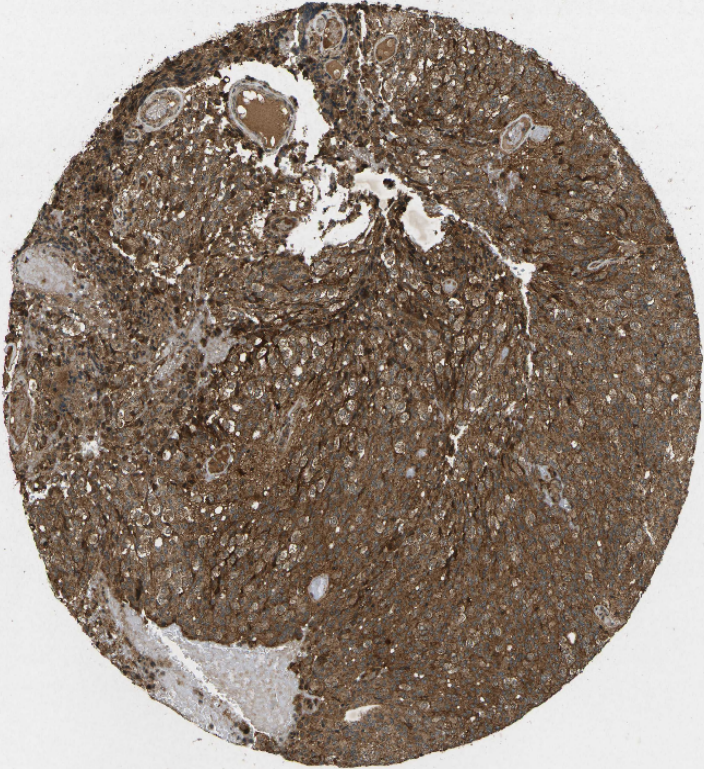

Supplement: Supplemental Information 10 [file peerj-11-15615-s010.zip › Figure 7/Figure 7E-2.pdf]

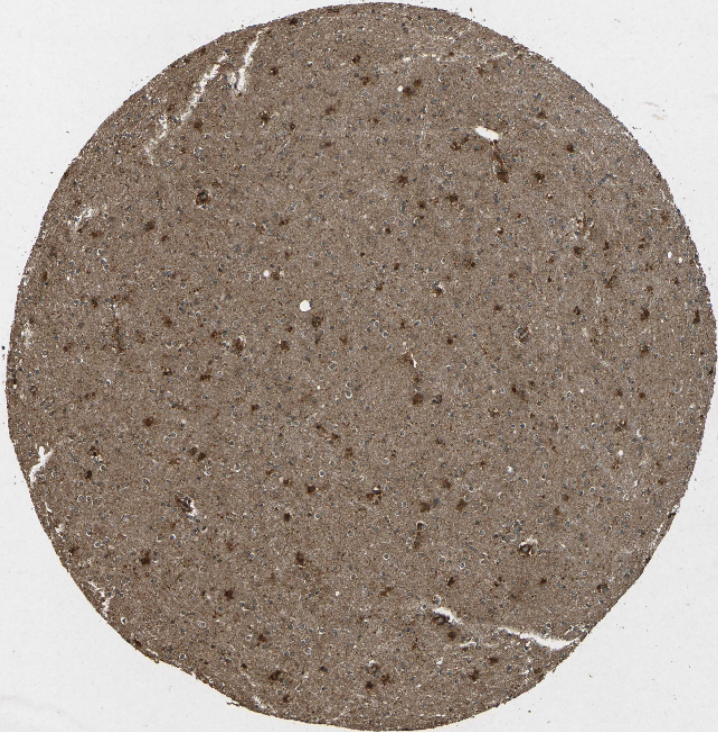

Supplement: Supplemental Information 10 [file peerj-11-15615-s010.zip › Figure 7/Figure 7E-1.pdf]

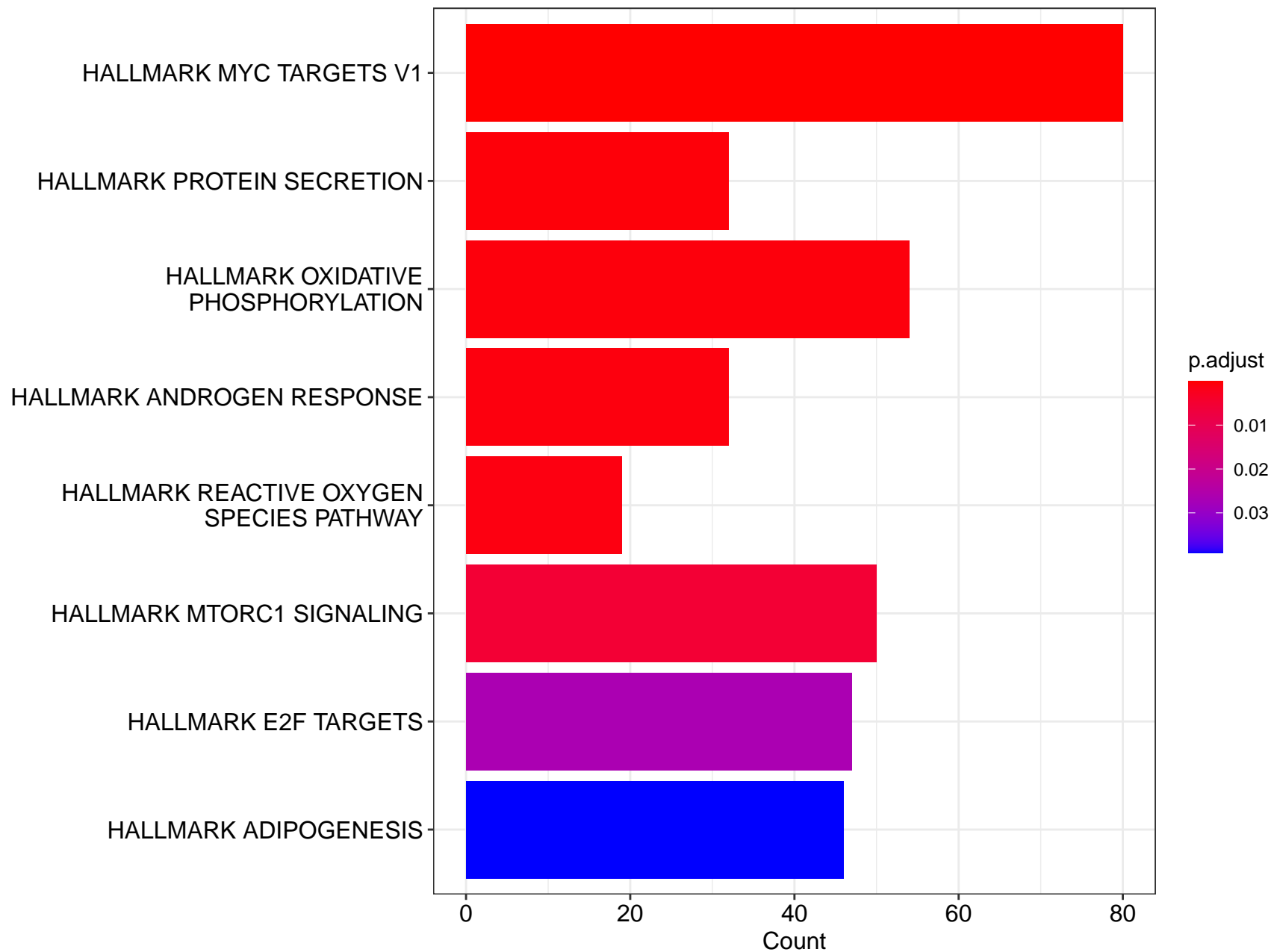

Supplement: Supplemental Information 10 [file peerj-11-15615-s010.zip › Figure 7/Figure 7F.pdf]

# MYD88

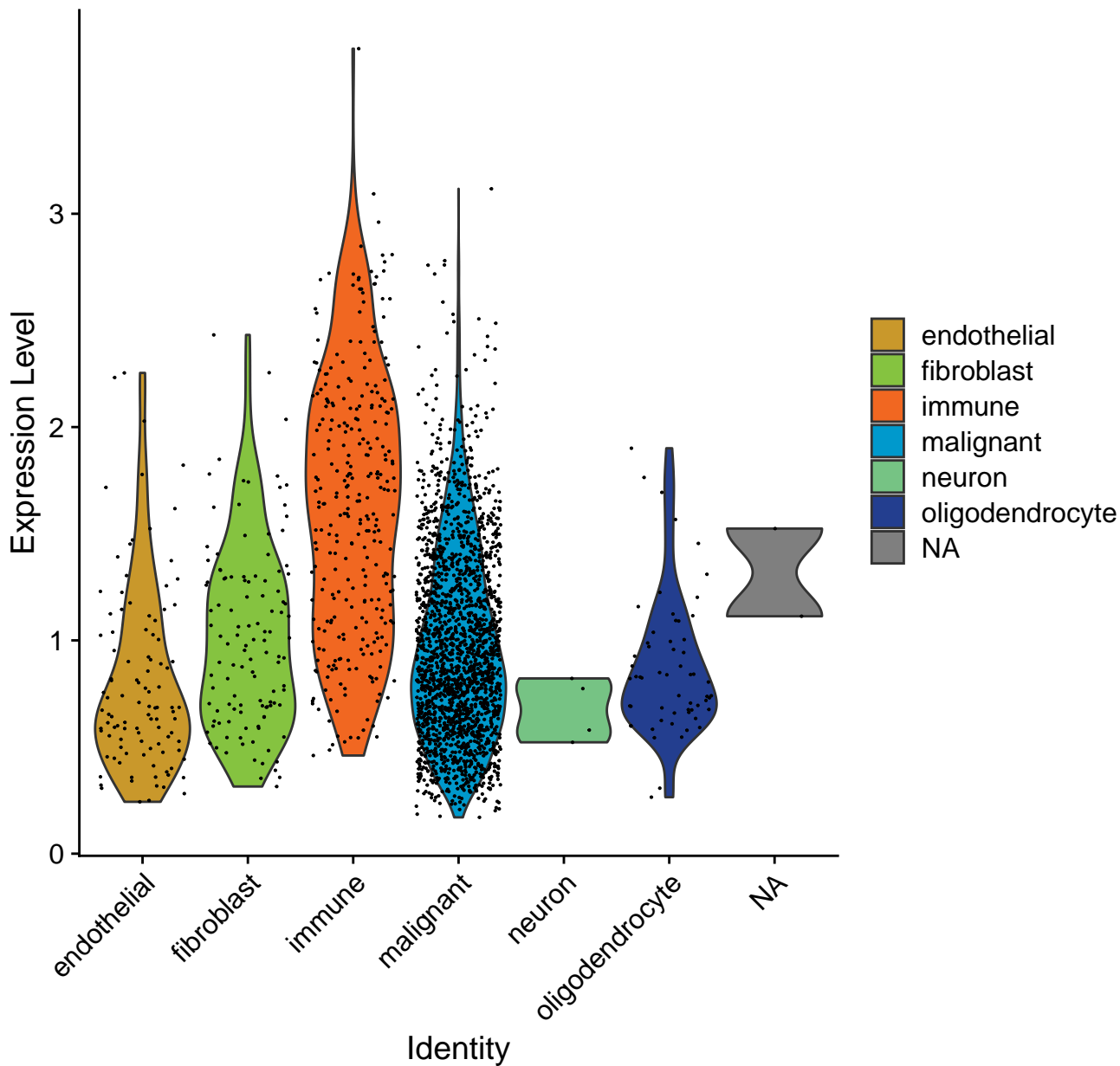

Supplement: Supplemental Information 10 [file peerj-11-15615-s010.zip › Figure 7/Figure 7D.pdf]

The expression of MYD88  
Log<sub>2</sub> (FPKM+1)

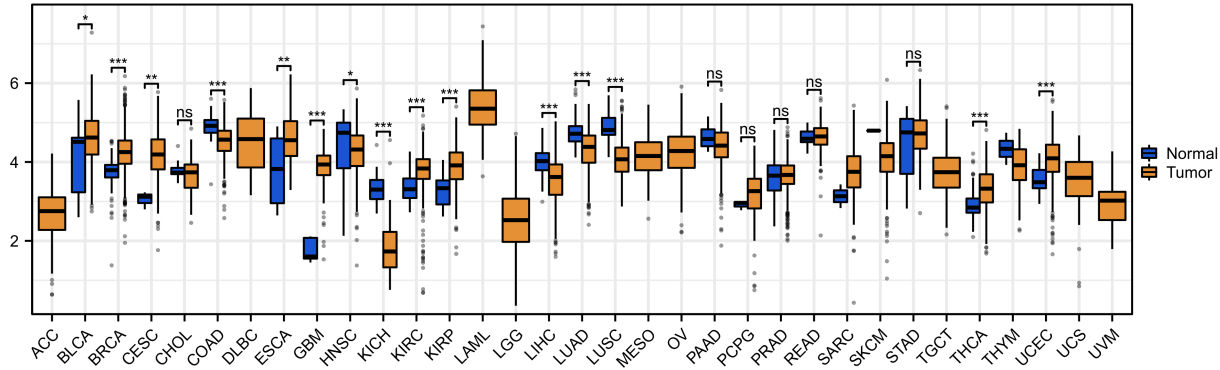

Supplement: Supplemental Information 10 [file peerj-11-15615-s010.zip › Figure 7/Figure 7A.pdf]

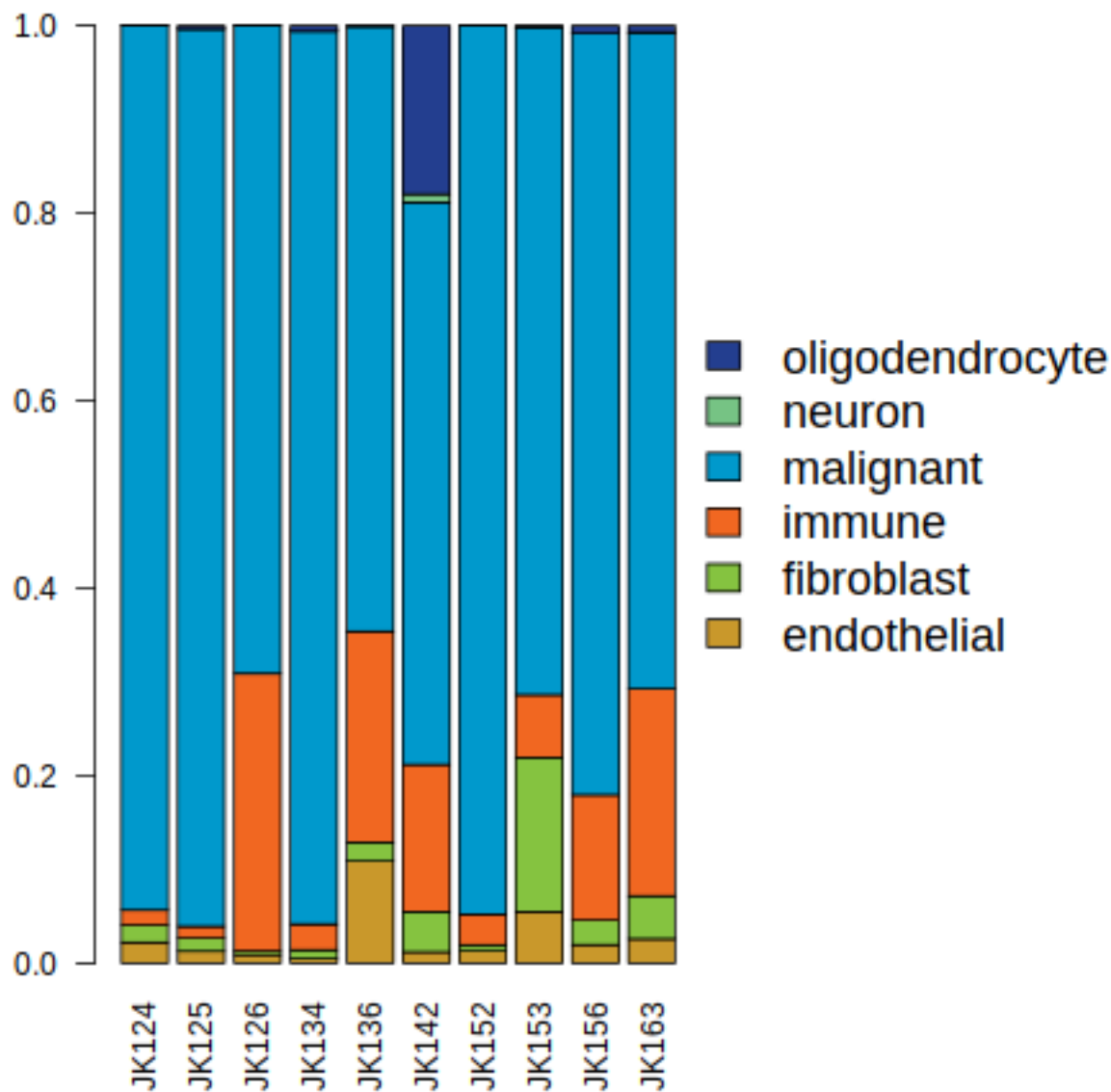

Supplement: Supplemental Information 10 [file peerj-11-15615-s010.zip › Figure 7/Figure 7C.pdf]

cell\_type

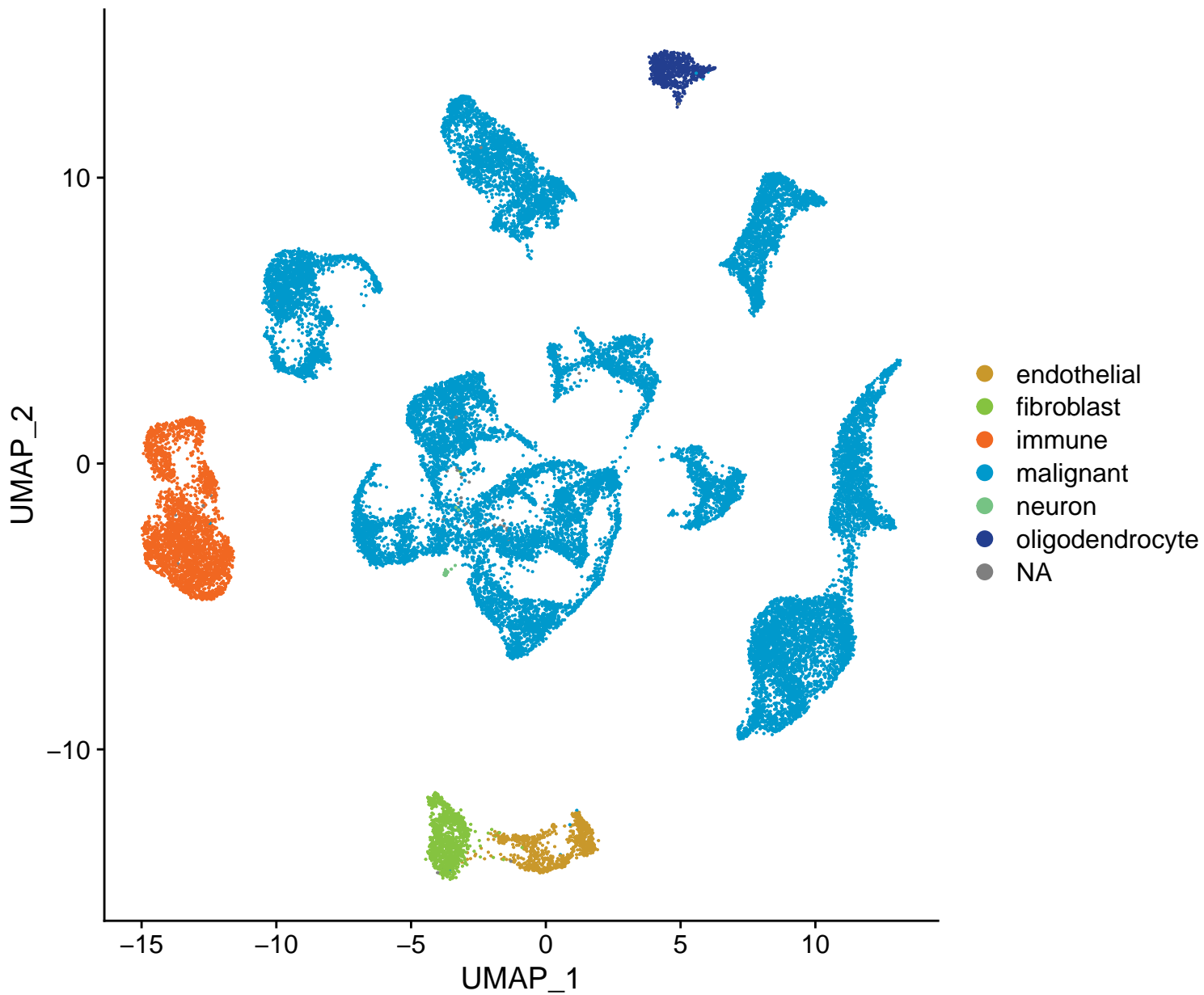

Supplement: Supplemental Information 10 [file peerj-11-15615-s010.zip › Figure 7/Figure 7B.pdf]

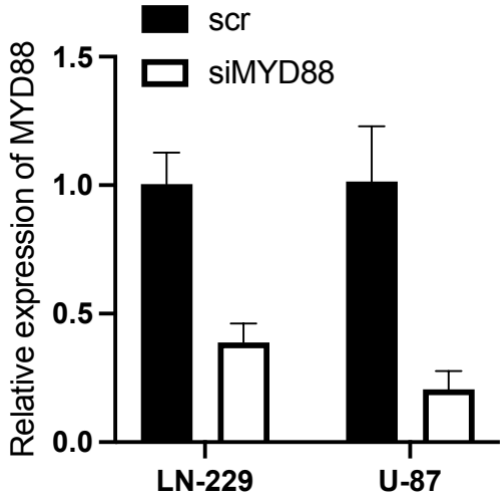

Supplement: Supplemental Information 11 [file peerj-11-15615-s011.zip › Figure 8/Figure 8A.pdf]

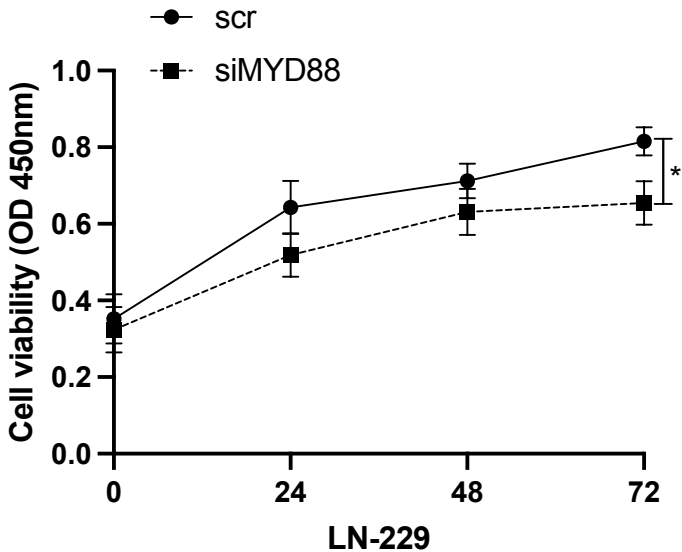

Supplement: Supplemental Information 11 [file peerj-11-15615-s011.zip › Figure 8/Figure 8B-1.pdf]

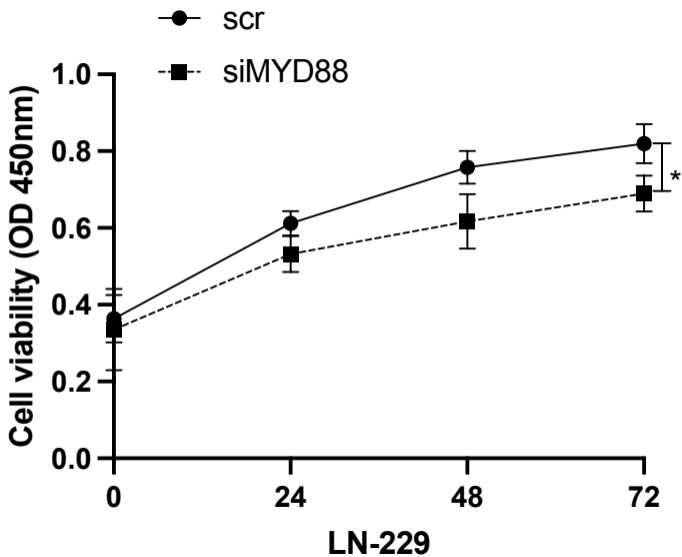

Supplement: Supplemental Information 11 [file peerj-11-15615-s011.zip › Figure 8/Figure 8B-2.pdf]

Number of invaded cells(% of control)

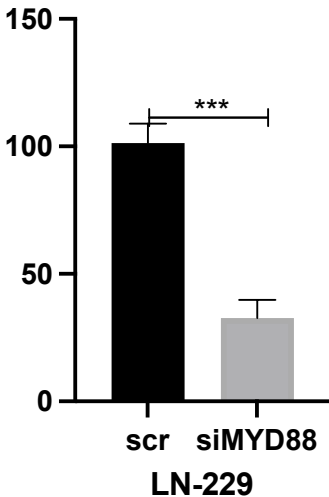

Supplement: Supplemental Information 11 [file peerj-11-15615-s011.zip › Figure 8/Figure 8C-5.pdf]

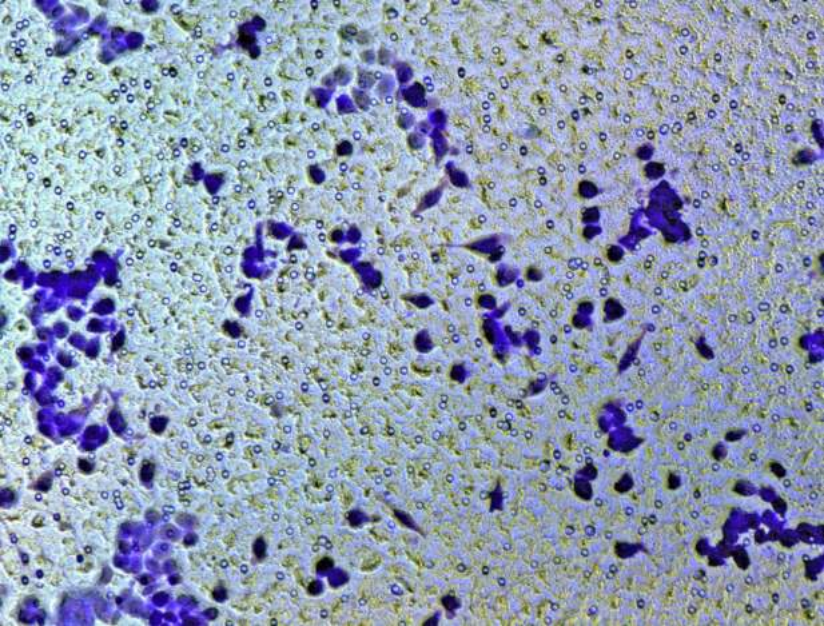

Supplement: Supplemental Information 11 [file peerj-11-15615-s011.zip › Figure 8/Figure 8C-4.pdf]

Number of invaded cells(% of control)

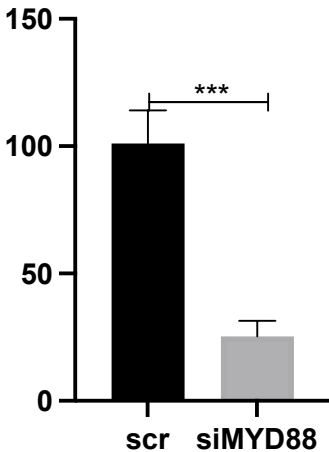

U-87

Supplement: Supplemental Information 11 [file peerj-11-15615-s011.zip › Figure 8/Figure 8C-6.pdf]

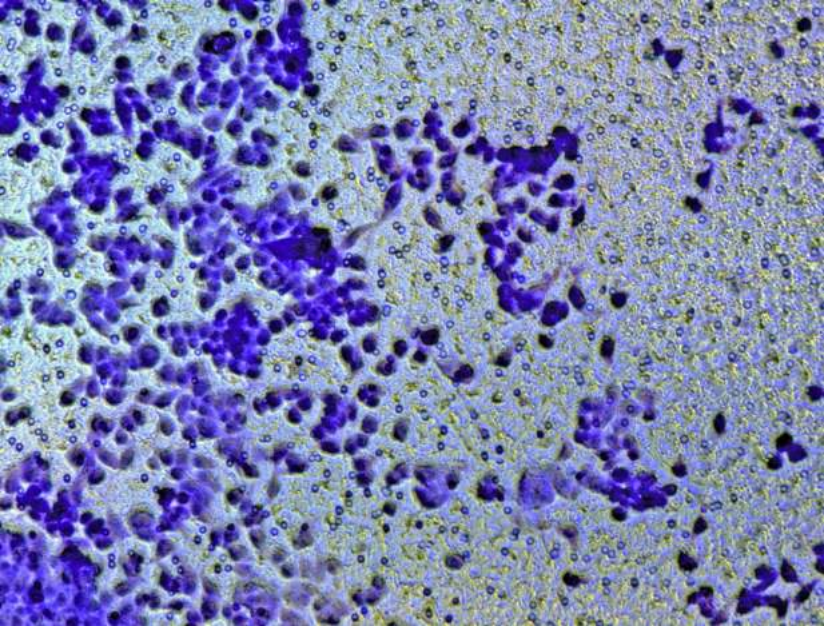

Supplement: Supplemental Information 11 [file peerj-11-15615-s011.zip › Figure 8/Figure 8C-3.pdf]

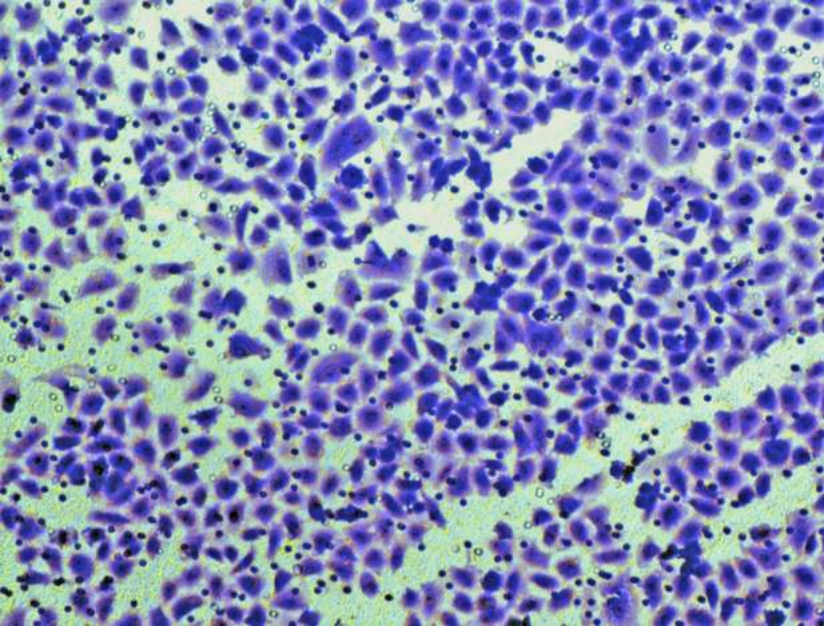

Supplement: Supplemental Information 11 [file peerj-11-15615-s011.zip › Figure 8/Figure 8C-2.pdf]

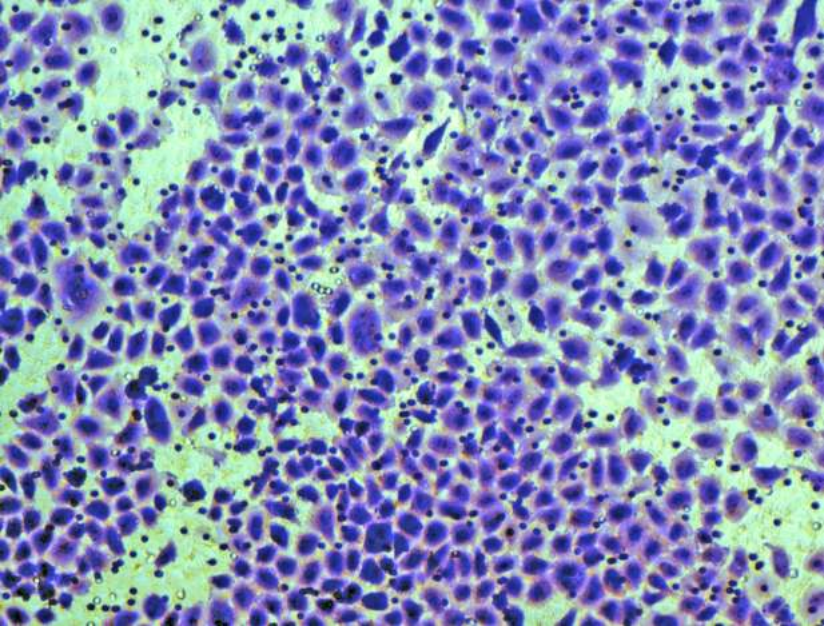

Supplement: Supplemental Information 11 [file peerj-11-15615-s011.zip › Figure 8/Figure 8C-1.pdf]

# LIGHT signaling pathway network

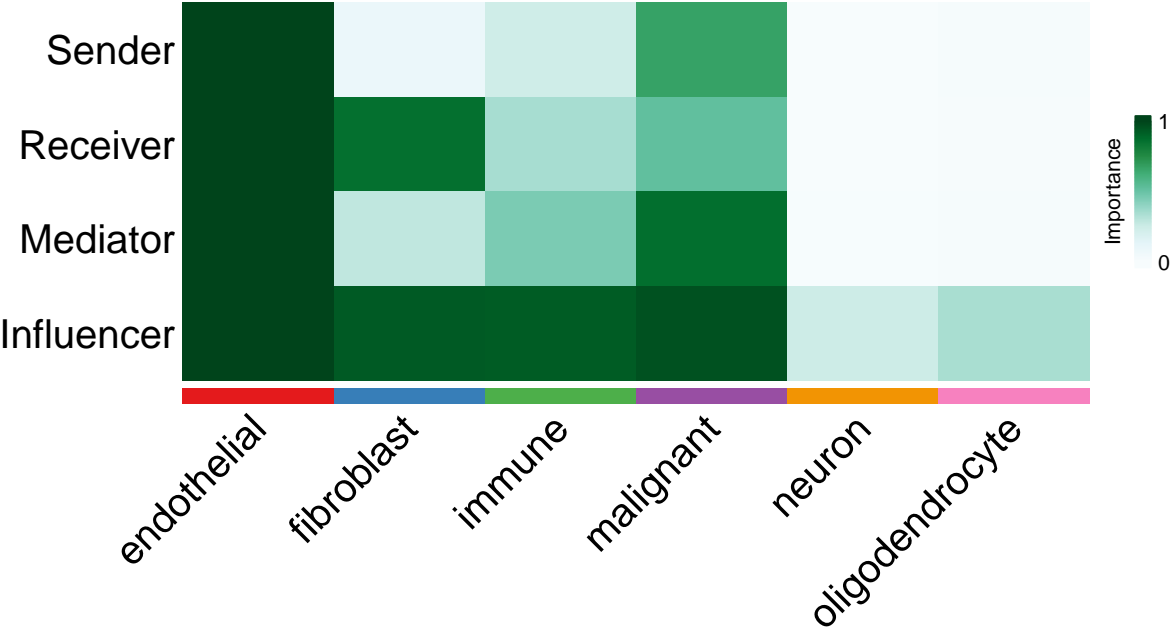

Supplement: Supplemental Information 12 [file peerj-11-15615-s012.zip › Figure 9/Figure 9D-2.pdf]

# PGF – VEGFR1

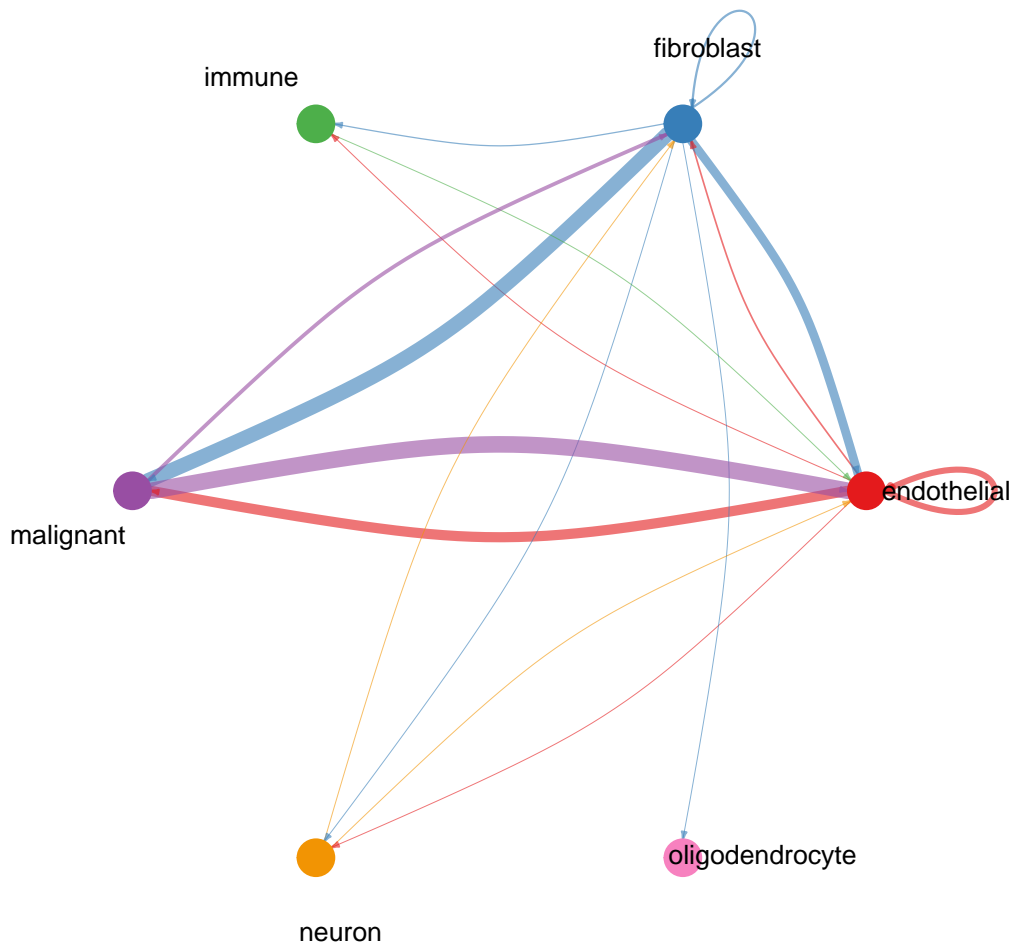

Supplement: Supplemental Information 12 [file peerj-11-15615-s012.zip › Figure 9/Figure 9F-1.pdf]

# TNFSF14 – LTBR

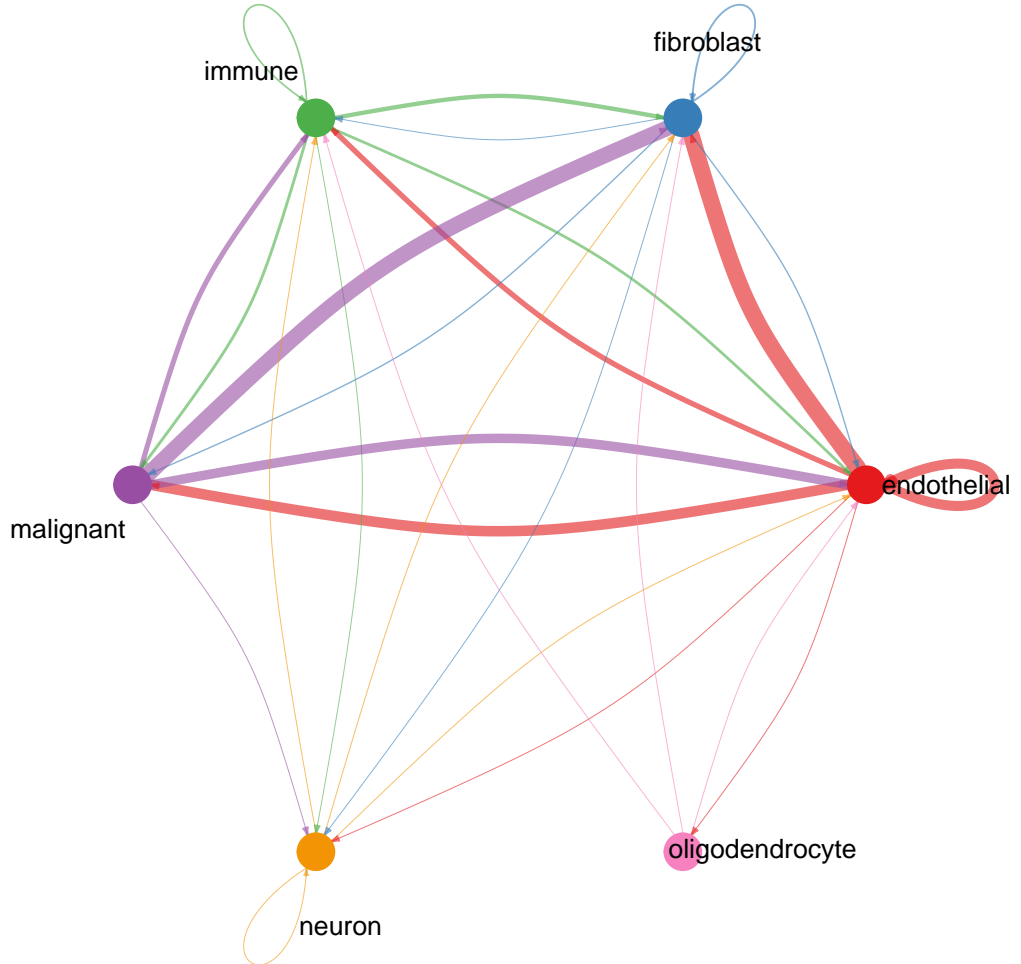

Supplement: Supplemental Information 12 [file peerj-11-15615-s012.zip › Figure 9/Figure 9D-1.pdf]

## VEGF signaling pathway network

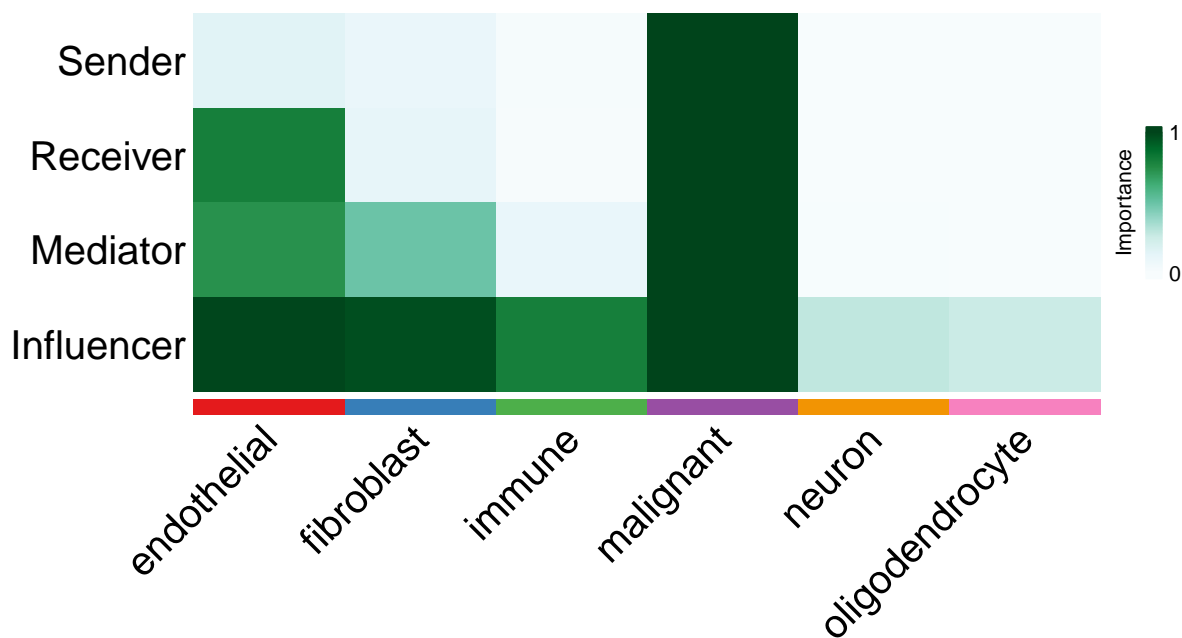

Supplement: Supplemental Information 12 [file peerj-11-15615-s012.zip › Figure 9/Figure 9F-2.pdf]

## GAS signaling pathway network

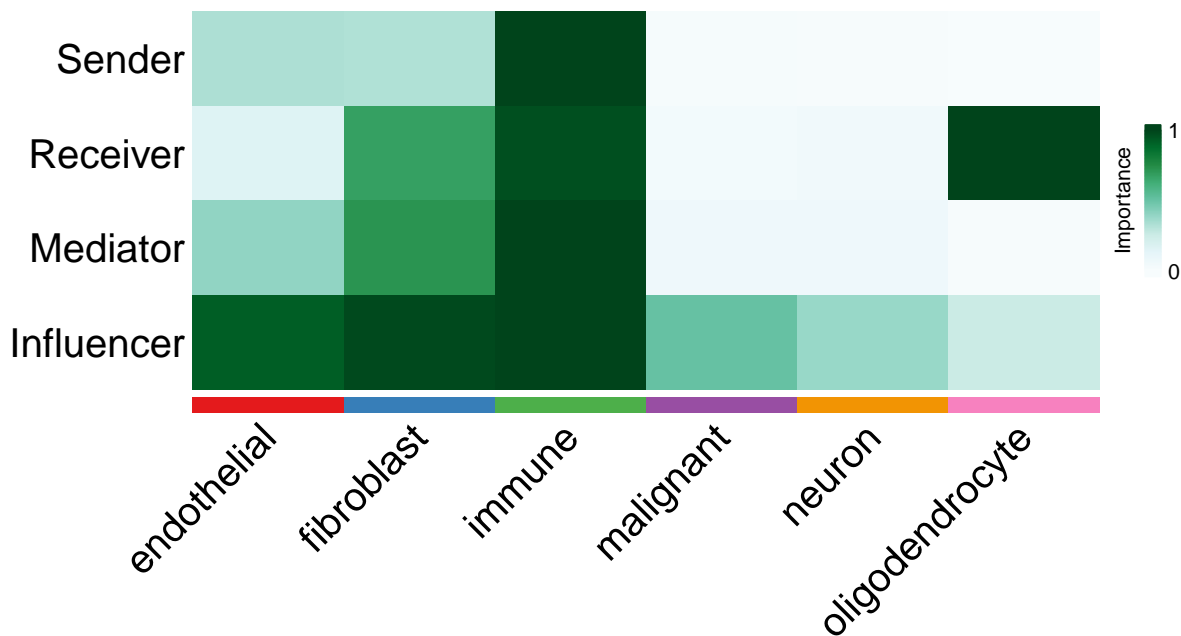

Supplement: Supplemental Information 12 [file peerj-11-15615-s012.zip › Figure 9/Figure 9B-2.pdf]

# GAS6 – AXL

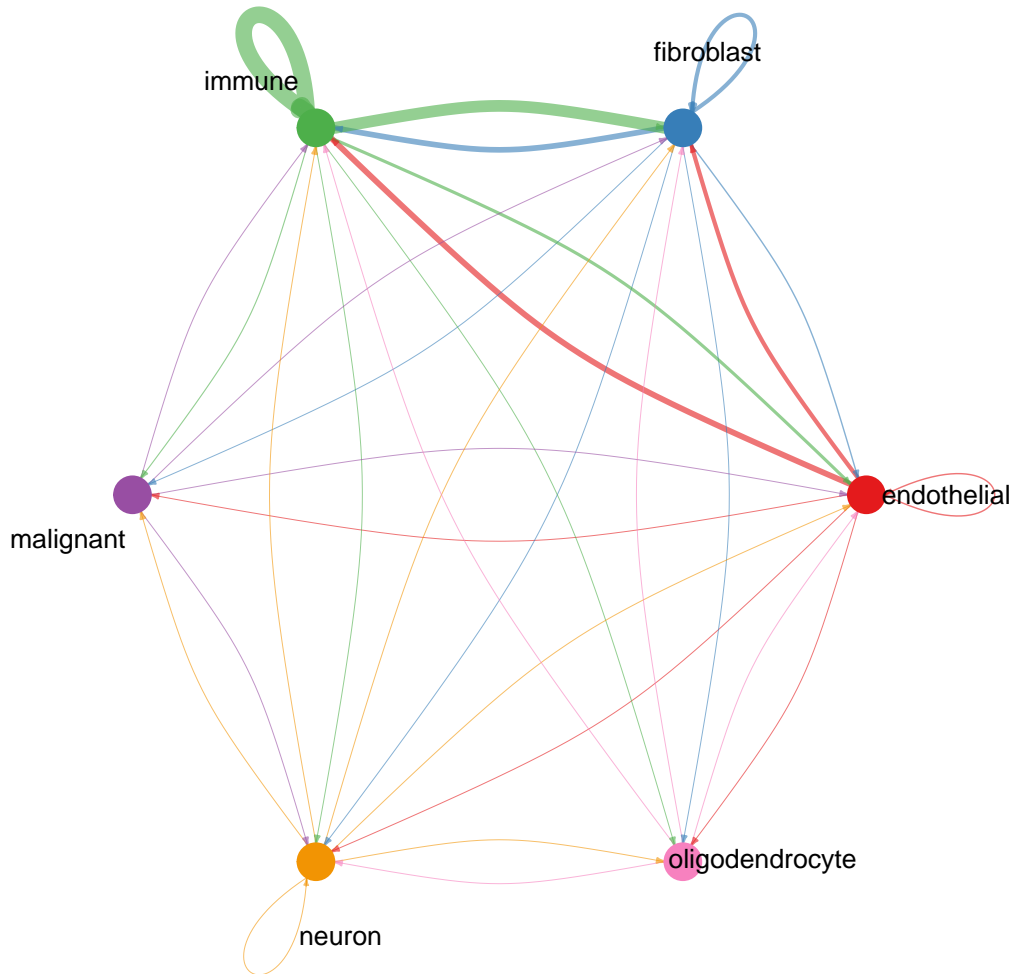

Supplement: Supplemental Information 12 [file peerj-11-15615-s012.zip › Figure 9/Figure 9B-1.pdf]

# LTA – TNFRSF1A

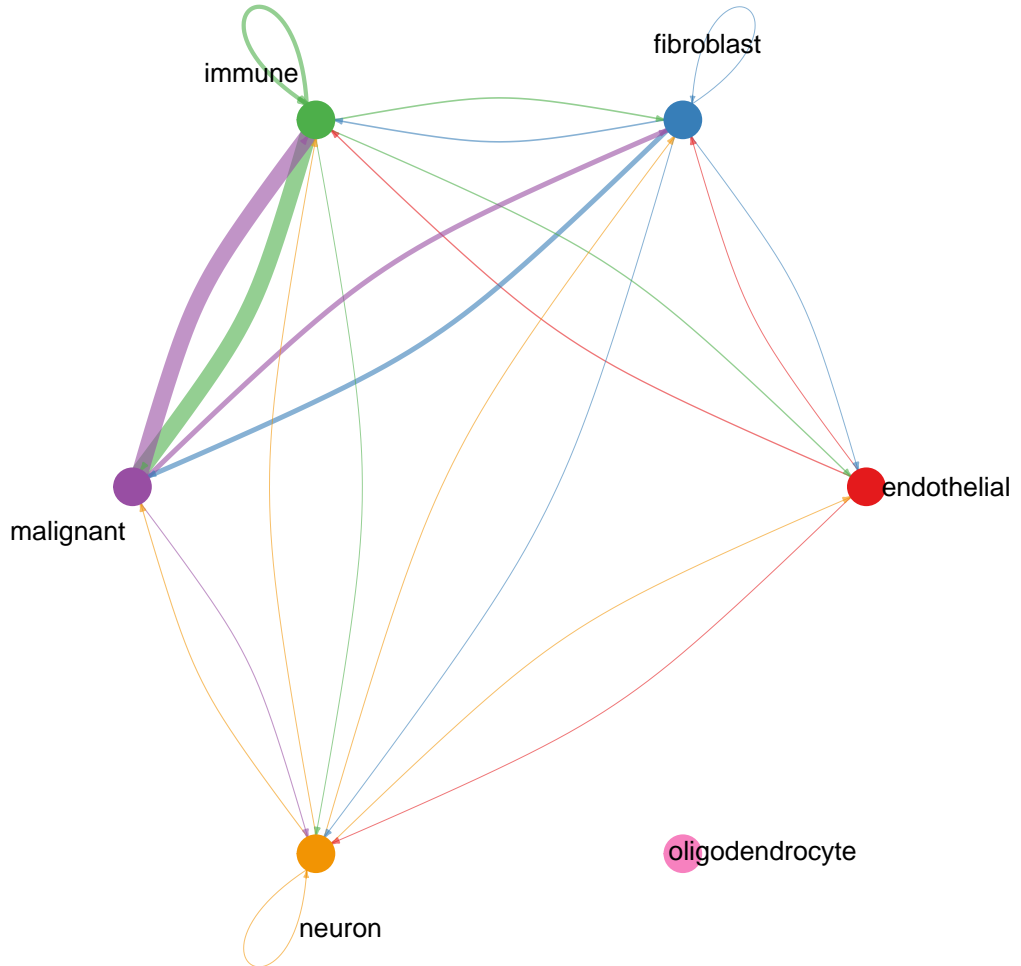

Supplement: Supplemental Information 12 [file peerj-11-15615-s012.zip › Figure 9/Figure 9E-1.pdf]

## LT signaling pathway network

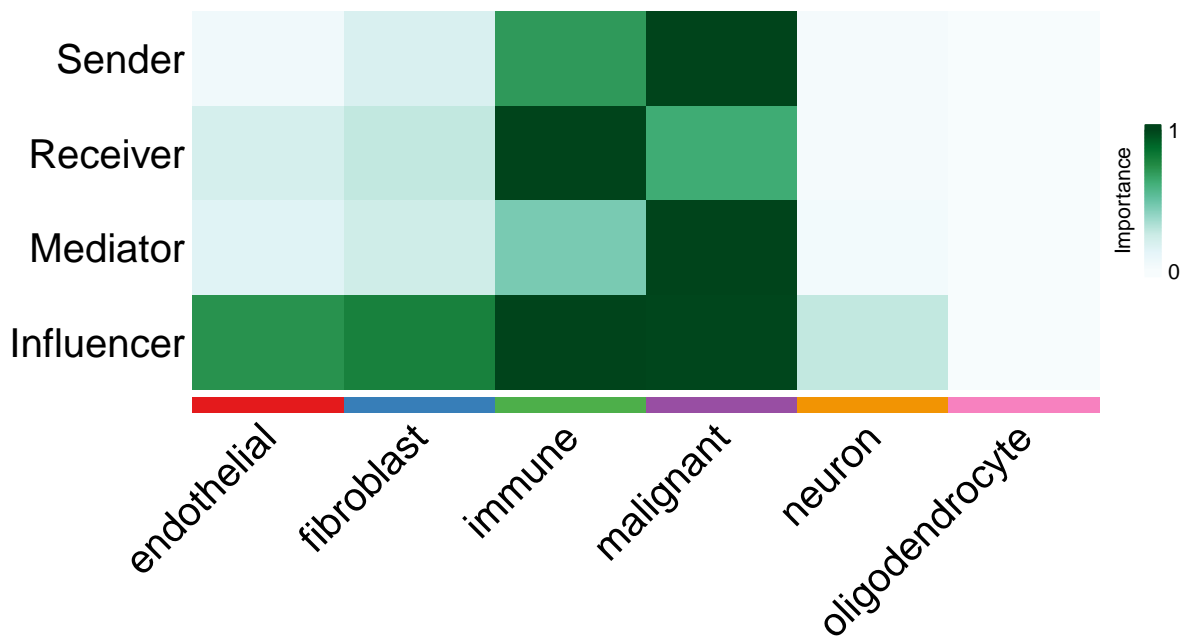

Supplement: Supplemental Information 12 [file peerj-11-15615-s012.zip › Figure 9/Figure 9E-2.pdf]

# GRN – SORT1

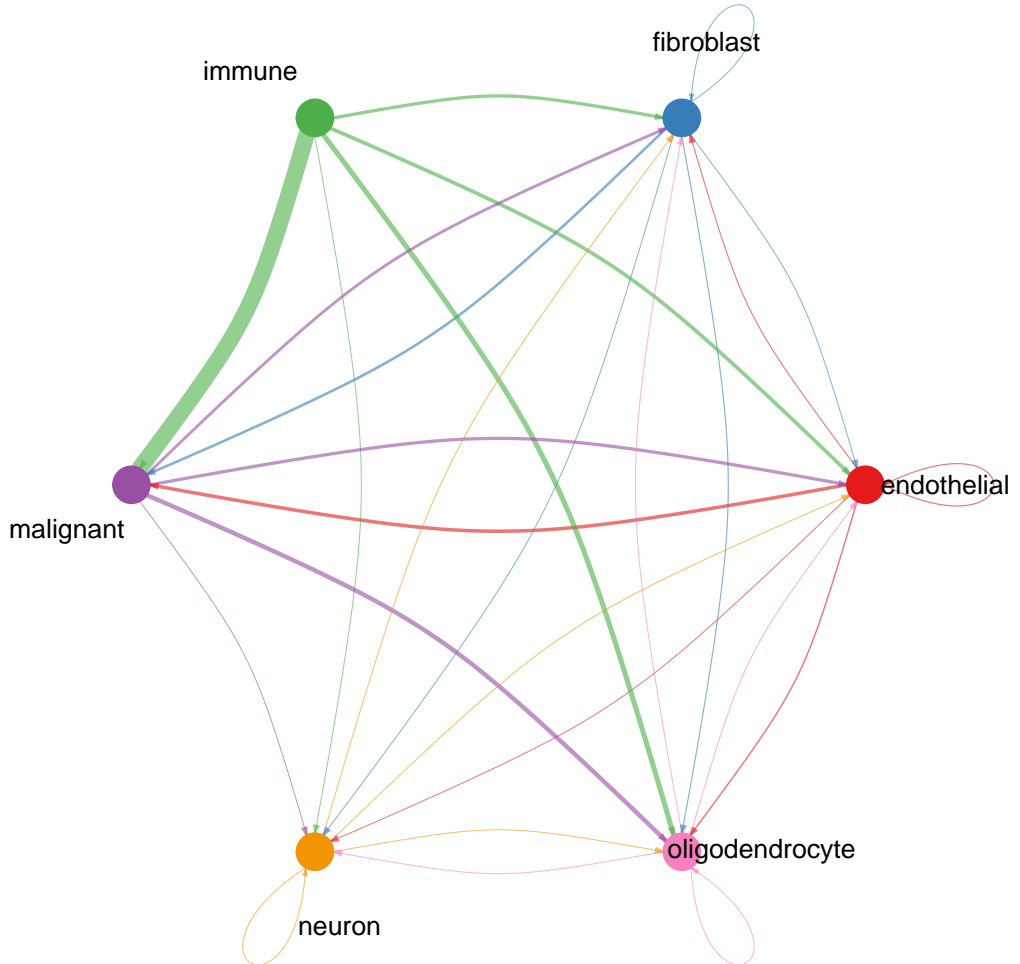

Supplement: Supplemental Information 12 [file peerj-11-15615-s012.zip › Figure 9/Figure 9C-1.pdf]

## ANGPTL signaling pathway network

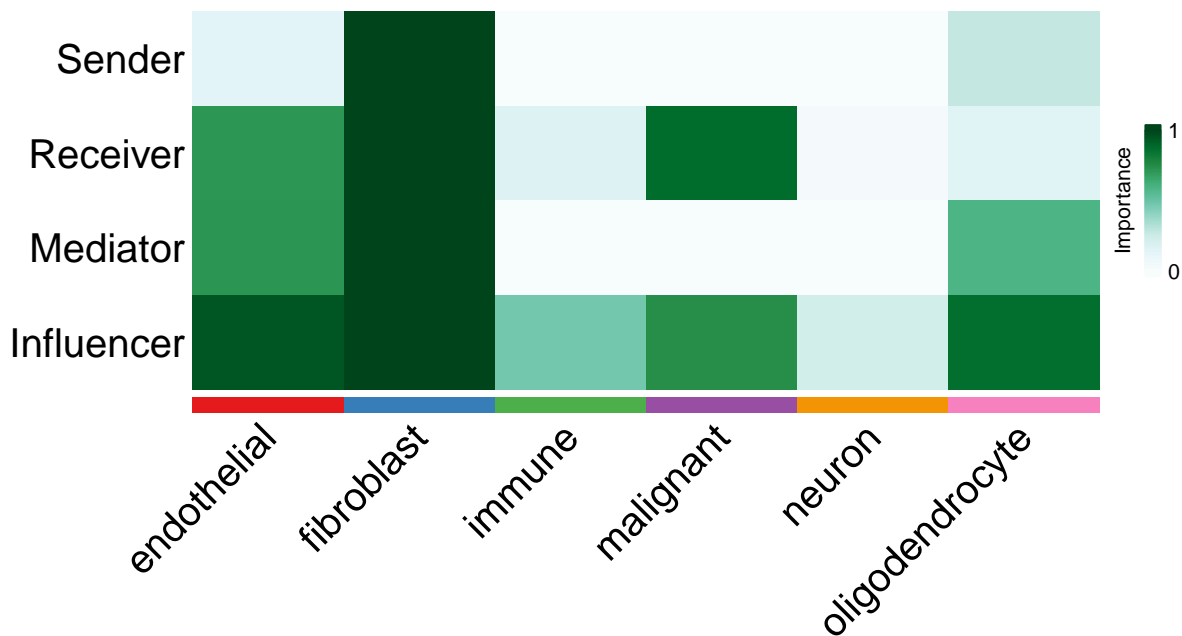

Supplement: Supplemental Information 12 [file peerj-11-15615-s012.zip › Figure 9/Figure 9A-2.pdf]

## GRN signaling pathway network

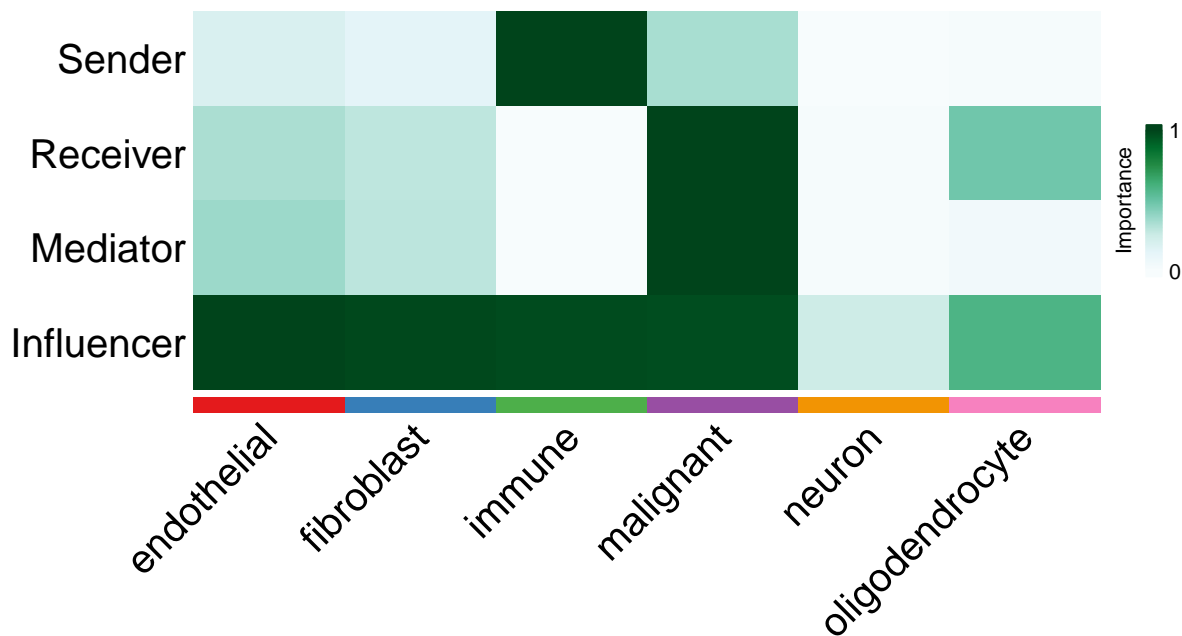

Supplement: Supplemental Information 12 [file peerj-11-15615-s012.zip › Figure 9/Figure 9C-2.pdf]

# ANGPTL2 – TLR4

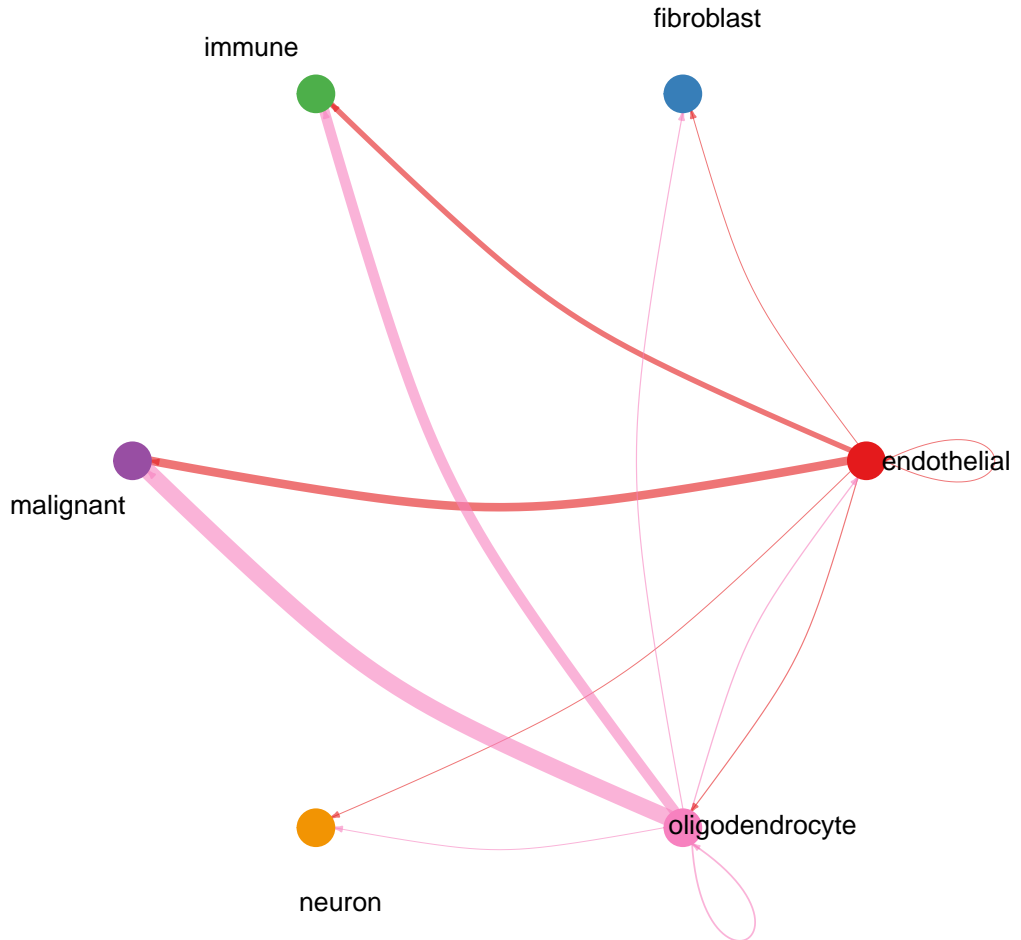

Supplement: Supplemental Information 12 [file peerj-11-15615-s012.zip › Figure 9/Figure 9A-1.pdf]

consensus matrix k=2

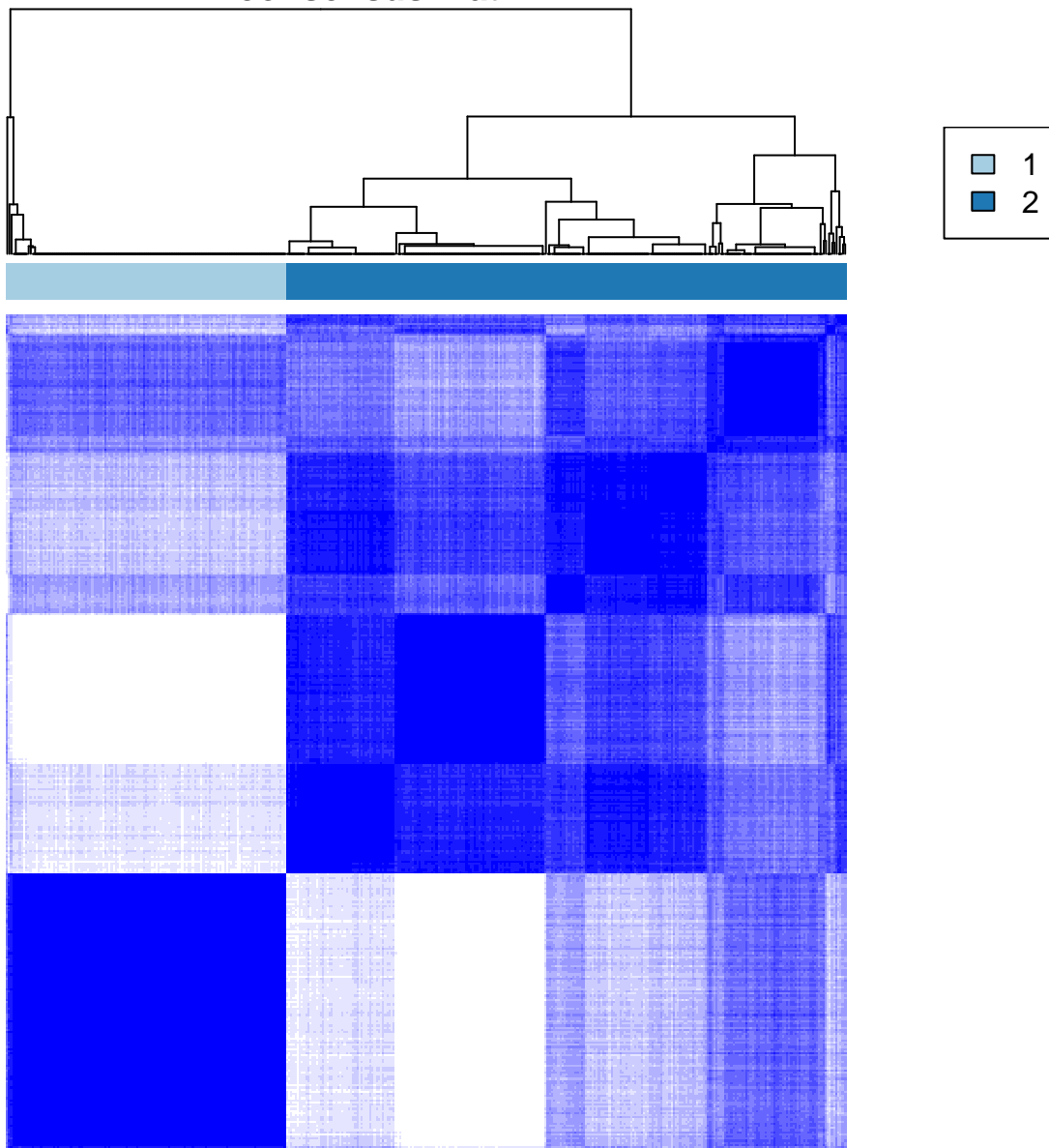

Supplement: Supplemental Information 13 [file peerj-11-15615-s013.zip › Figure S1/k=2.pdf]

consensus matrix k=5

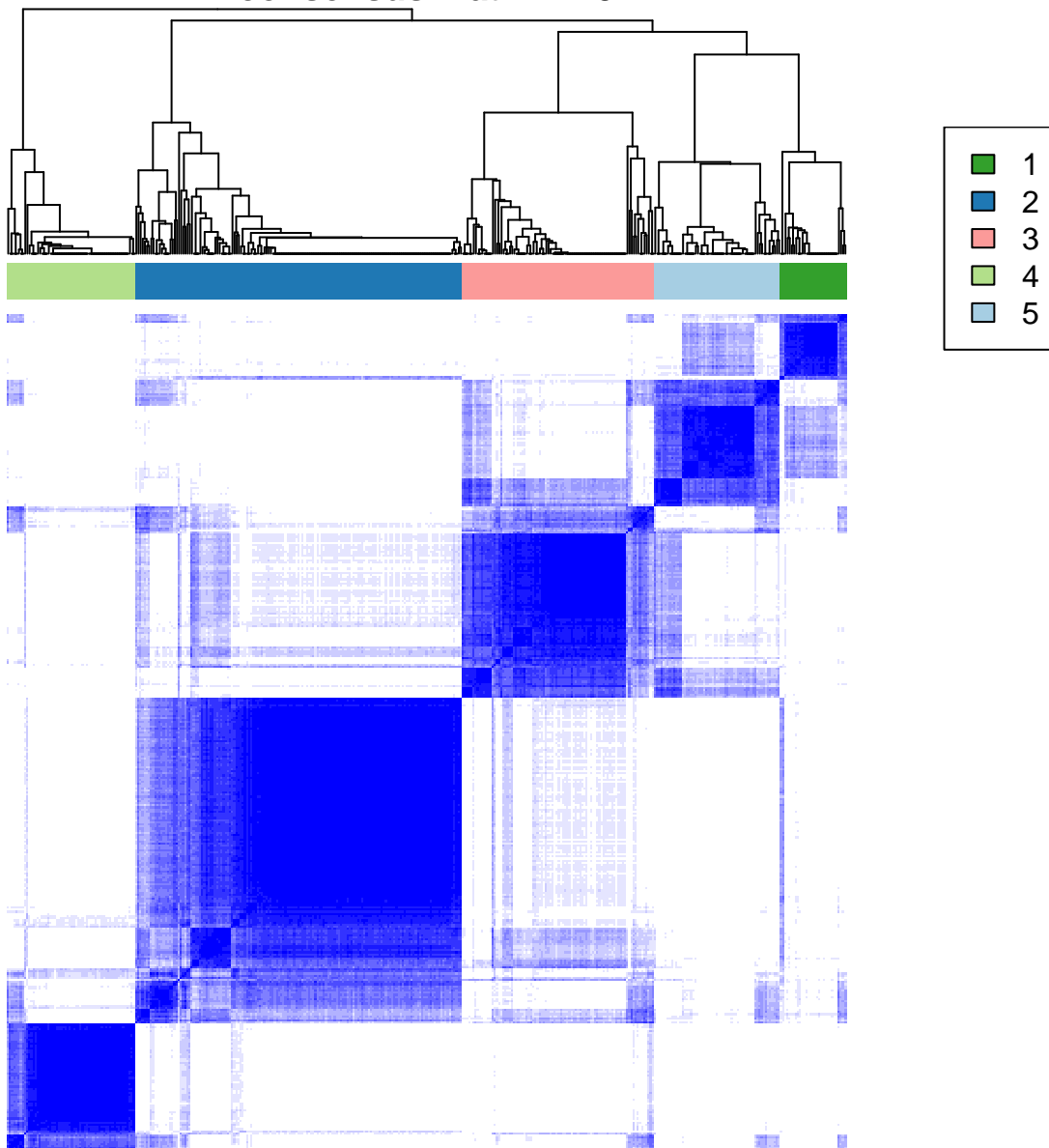

Supplement: Supplemental Information 13 [file peerj-11-15615-s013.zip › Figure S1/k=5.pdf]

consensus matrix k=4

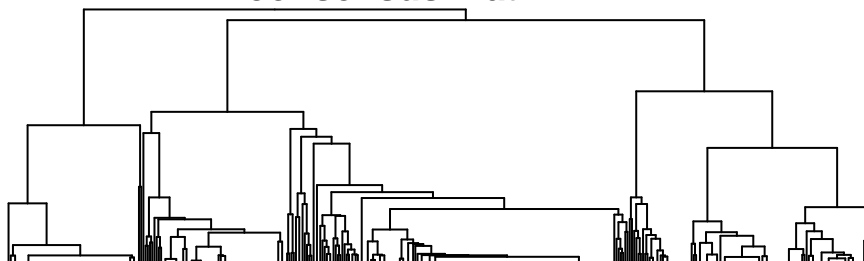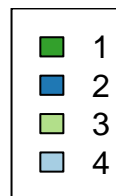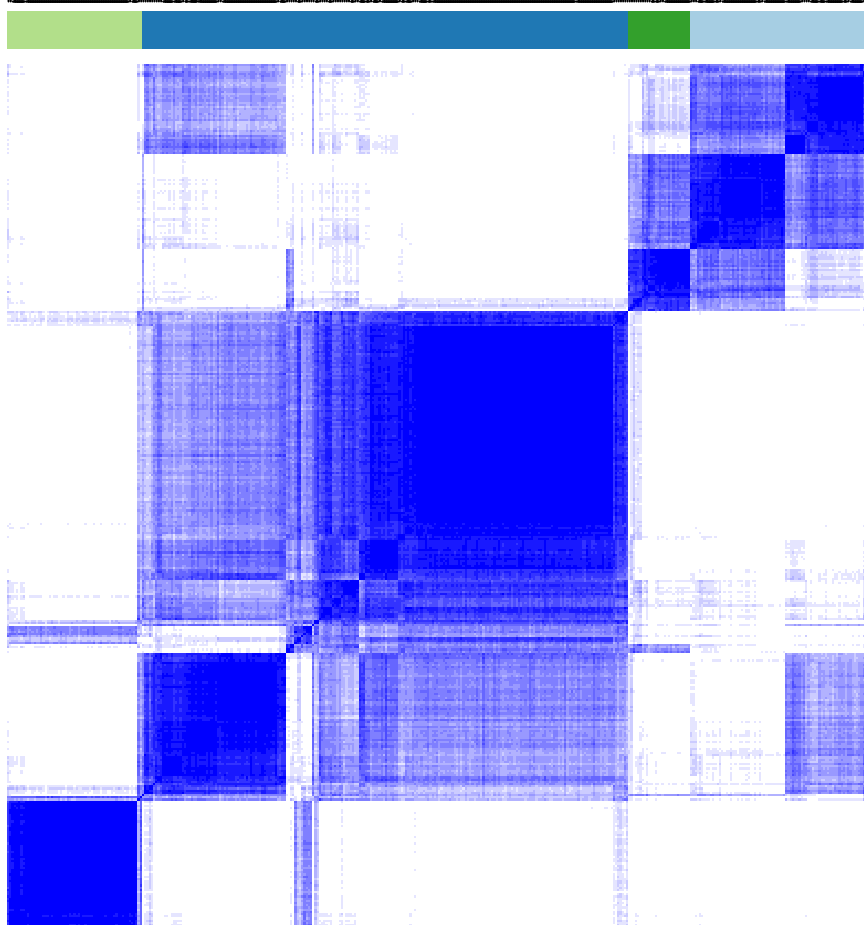

Supplement: Supplemental Information 13 [file peerj-11-15615-s013.zip › Figure S1/k=4.pdf]

consensus matrix k=6

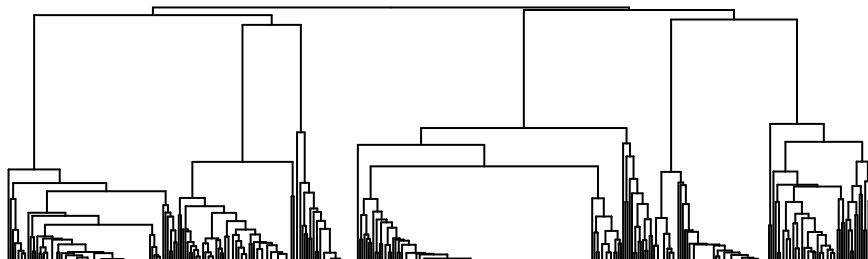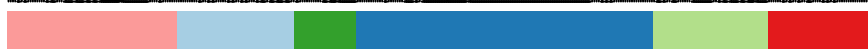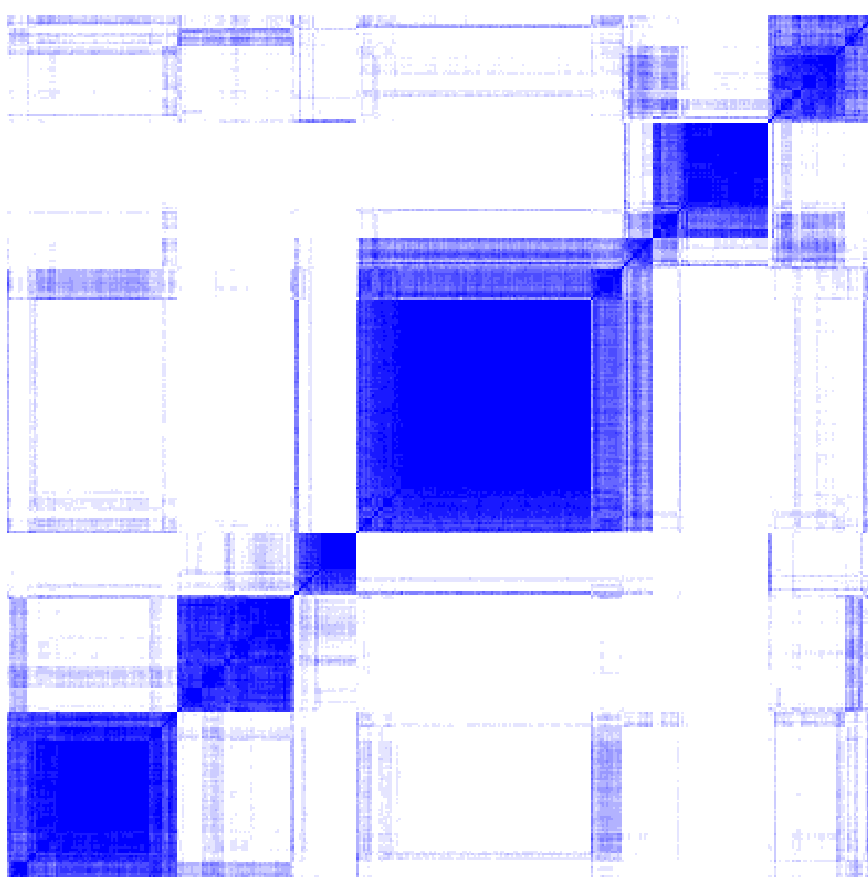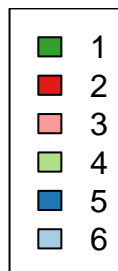

Supplement: Supplemental Information 13 [file peerj-11-15615-s013.zip › Figure S1/k=6.pdf]

consensus matrix k=7

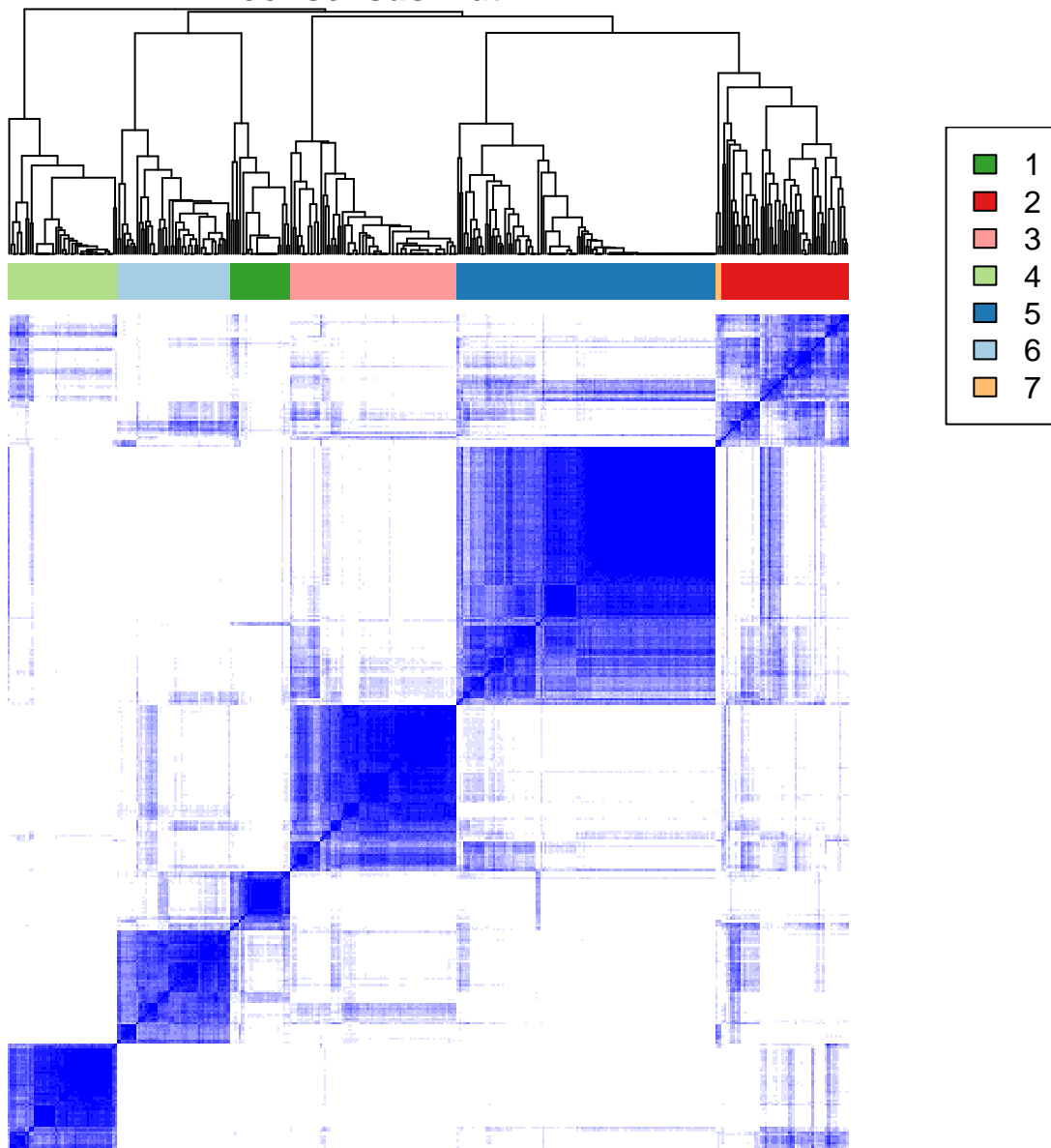

Supplement: Supplemental Information 13 [file peerj-11-15615-s013.zip › Figure S1/k=7.pdf]

consensus matrix k=8

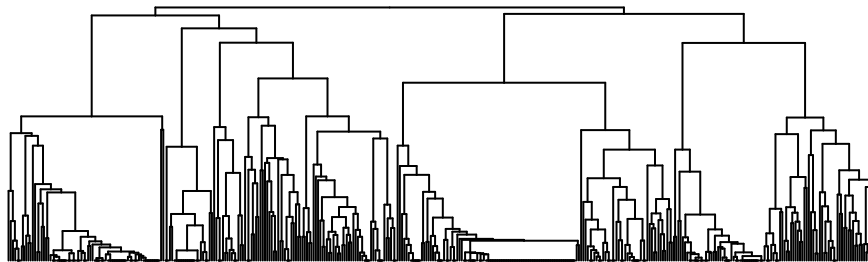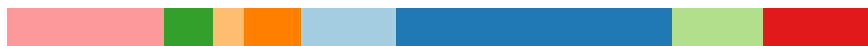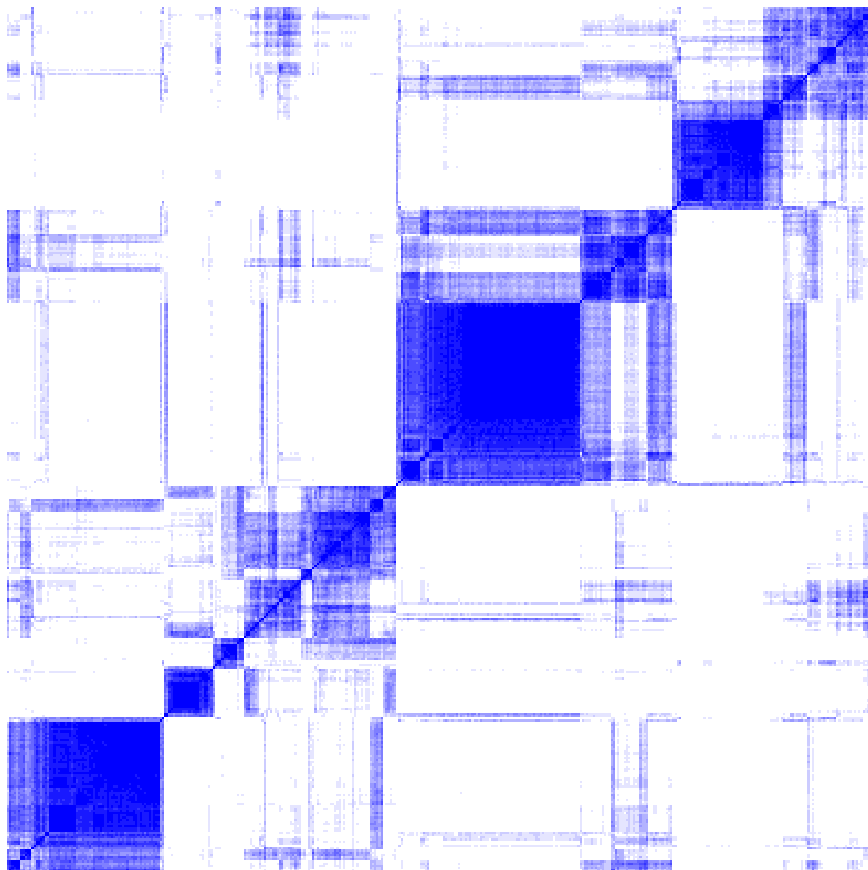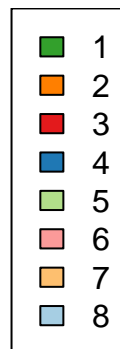

Supplement: Supplemental Information 13 [file peerj-11-15615-s013.zip › Figure S1/k=8.pdf]
