# Supplementary material for: A novel immunogenic cell death signature for the prediction of prognosis and therapies in glioma
Source: PeerJ. 2023 Jul 11;11:e15615. doi: 10.7717/peerj.15615 (PMC10348309; doi:10.7717/peerj.15615)
Supplement: Supplemental Information 4 [file peerj-11-15615-s004.zip › Figure 1/Figure 1A.pdf]

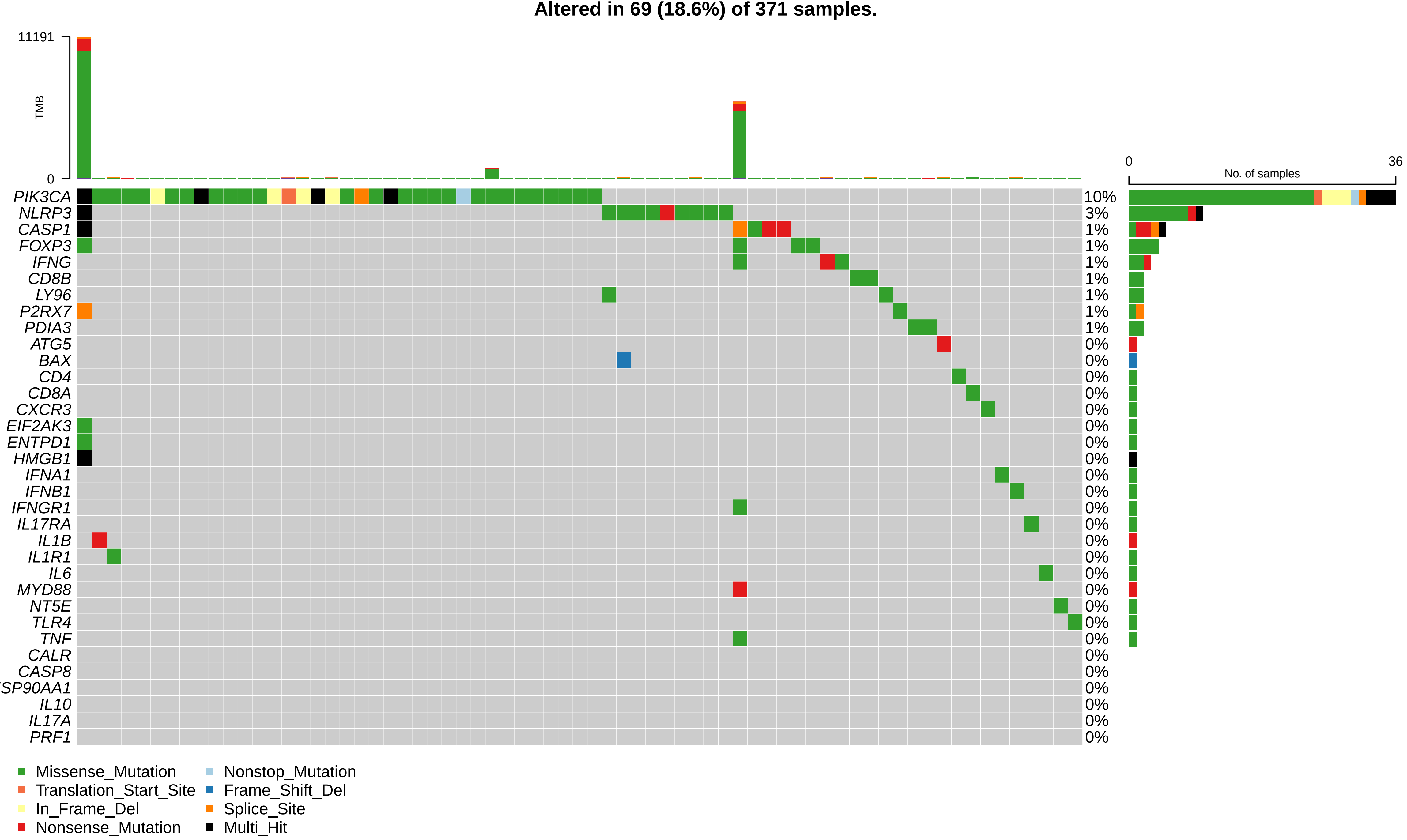

Altered in 69 (18.6%) of 371 samples.
11191
TMB
0
0
No. of samples
36
PIK3CA 10%
NLRP3 3%
CASP1 1%
FOXP3 1%
IFNG 1%
CD8B 1%
LY96 1%
P2RX7 1%
PDIA3 1%
ATG5 0%
BAX 0%
CD4 0%
CD8A 0%
CXCR3 0%
EIF2AK3 0%
ENTPD1 0%
HMGB1 0%
IFNA1 0%
IFNB1 0%
IFNGR1 0%
IL17RA 0%
IL1B 0%
IL1R1 0%
IL6 0%
MYD88 0%
NT5E 0%
TLR4 0%
TNF 0%
CALR 0%
CASP8 0%
SP90AA1 0%
IL10 0%
IL17A 0%
PRF1 0%
Missense_Mutation
Translation_Start_Site
In_Frame_Del
Nonsense_Mutation
Nonstop_Mutation
Frame_Shift_Del
Splice_Site
Multi_Hit
